# Characterisation and Comparison of Lactating Mouse and Bovine Mammary Gland miRNomes

**DOI:** 10.1371/journal.pone.0091938

**Published:** 2014-03-21

**Authors:** Sandrine Le Guillou, Sylvain Marthey, Denis Laloë, Johann Laubier, Lenha Mobuchon, Christine Leroux, Fabienne Le Provost

**Affiliations:** 1 INRA, UMR1313 Génétique Animale et Biologie Intégrative, Jouy-en-Josas, France; 2 INRA, UMR1213 Herbivores, Saint-Genès Champanelle, France; 3 Clermont Université, VetAgro Sup, UMR1213 Herbivores, Clermont-Ferrand, France; French National Center for Scientific Research - Institut de biologie moléculaire et cellulaire, France

## Abstract

**Background:**

The mammary gland is a dynamic organ that undergoes important physiological changes during reproductive cycles. Until now, data regarding the characterisation of miRNA in the mammary gland have been scarce and mainly focused on their abnormal expression in breast cancer. Our goal was to characterise the microRNA (miRNA) involved in mechanisms regulating the mammary function, with particular focus on the lactation stage.

**Methodology/principal findings:**

Using high-throughput sequencing technology, the exhaustive repertoires of miRNA expressed (miRNome) in mouse and bovine mammary glands during established lactation were identified, characterized and compared. Furthermore, in order to obtain more information on miRNA loading in the RNA-induced silencing complex (RISC), the miRNome was compared with that obtained from RNA associated with the AGO2 protein (AGO2-miRNome) in mouse lactating mammary gland. This study enabled the identification of 164 and 167 miRNA in mouse and bovine, respectively. Among the 30 miRNA most highly expressed in each species, 24 were common to both species and six of them were preferentially highly expressed in lactating than non-lactating mammary gland. The potential functional roles of these 24 miRNA were deduced using DIANA-miRPath software, based on miRNA/mRNA interactions. Moreover, seven putative novel miRNA were identified. Using DAVID analysis, it was concluded that the predicted targets of two of these putative novel miRNA are involved in mammary gland morphogenesis.

**Conclusion/significance:**

Our study provides an overview of the characteristics of lactating mouse and bovine mammary gland miRNA expression profiles. Moreover, species-conserved miRNA involved in this fundamental biological function were identified. These miRNomes will now be used as references for further studies during which the impact of animal breeding on the miRNA expression will be analysed.

## Introduction

MicroRNA (miRNA), or endogenous small RNA (18–22 nt), are negative regulators of gene expression, capable of exerting pronounced effects on the translation and stability of mRNA [1]–[3]. It is estimated that miRNA genes may account for 2% to 5% of all mammalian genes and collectively regulate the expression of up to 60% of protein-coding genes [4], [5]. miRNA-mediated gene regulation is crucial to all biological processes such as cellular growth, cell differentiation or death [6]–[8], as well as metabolism [9]–[12]. Moreover, most of miRNA are expressed in a spatio-temporal pattern [13], suggesting that they play specific functions [8]. Despite considerable growth in their number in the miRBase database (http://microrna.sanger.ac.uk/), the field of miRNA research remains uncharted territory.

miRNA-mediated regulation is thought to require a minimally stable interaction between an mRNA and an miRNA and an Argonaute (AGO) protein, comprising a tightly associated miRNA-associated ribonucleoprotein (miRNP), which is the core of the RNA-induced silencing complex (RISC) [14], [15]. In mammals, the AGO subfamily comprises four proteins (AGO1-4). Whilst all AGO proteins are translation repressors, only AGO2 exerts endonuclease activity (reviewed in [16]). This protein family is used to identify mRNA targets using the immunopurification techniques which have recently been developed to isolate RISC associated mRNA and miRNA [17]. In two recent studies [18], [19] based on the cloning and deep sequencing of endogenous miRNA associated with AGO1-3, no evidence for major miRNA sorting was found in human cells. Until now, the correlation between the miRNome obtained from complete tissue and that associated with AGO proteins has only been weakly documented [20].

Studies of miRNA in mammary gland have mostly focused on human breast cancer, and functional studies have identified miRNA playing both tumour suppressing and oncogenic roles by targeting the mRNA involved in breast cancer [21]. However, the mammary gland is a dynamic organ that undergoes important physiological changes during the female reproductive cycle. One important stage in this cycle is lactation, when the mammary gland is mainly made up of mammary epithelial cells which are involved in specific biological functions such as milk component biosynthesis and secretion [22]–[24].

Relatively little is known about the role of miRNA during normal mammary gland development or lactation. Ucar and colleagues [25] showed that *miR-212* and *miR-132* are essential during mammary gland development. A link between miRNA and mammary epithelial progenitor cells has been evidenced using the Comma-Dbeta mouse mammary epithelial cell line [26], [27]. The regulation of both epithelial-mesenchymal transition (EMT) and EMT-associated stem cell properties via the tumour suppressor p53-mediated transcriptional activation of *miR-200c* has also been demonstrated [28]. Le Guillou *et al.* [29] recently showed that the over expression of an miRNA, *miR-30b*, in the mouse mammary gland results in a lactation defect characterised by the presence of acini structures with abnormally small lumen and defective of lipid droplet formation. These studies thus revealed the crucial role of miRNA with respect to mammary gland biology.

miRNA expression patterns have been described in normal mouse and ruminant mammary glands at different stages [30]–[35], but the miRNA profiles obtained were not exhaustive because of limitations to the technologies used. However, high-throughput sequencing technology has now become a powerful tool to describe an exhaustive miRNA repertoire and discover new miRNA [36], [37].

Whilst very recent studies of miRNA repertories in goat and bovine mammary gland tissues during dry period, early, peak and late lactation have been performed using high-throughput sequencing techniques [38]–[41], the description of mammary gland miRNomes is still limited. In particular, no reports on the miRNA repertoire in the mouse mammary gland, or any comparisons between species to characterise conserved miRNA in this biological function, have been presented.

The principal objective of the work reported here was therefore to identify the crucial miRNA involved in lactation. We thus identified and compared the exhaustive repertoires of miRNA, known and novel, expressed in mouse and bovine mammary glands during established lactation using Solexa deep sequencing technology. We characterised a set of miRNA that are conserved and highly expressed during lactation, as well as miRNA related to different species and different physiological stages. To widen the knowledge on miRNA loading in RISC, we also compared the whole miRNome *versus* the miRNome associated with the AGO2 protein, or the AGO2-miRNome, in the lactating mouse mammary gland. The predicted biological processes targeted by the most frequently detected miRNA were then discussed.

## Materials and Methods

### Animals and tissue collection

All experiments involving animals were performed in strict accordance with the guidelines of the Code for Methods and Welfare Considerations in Behavioural Research with Animals (Directive 86/609EC) and the recommendations of the French Commission de Génie Génétique (Permit number 12931 (01.06.2003)) and CEMEAA (Ethics Committee for Animal Experimentation in Auvergne, number 02) which approved this study. Every effort was made to minimise animal suffering.

Left and right abdominal mammary glands (#4) were collected from two primiparous FVB/N mice at mid-lactation (day-12). The day of parturition was designated as day 0 of lactation. No litters containing fewer than five pups were used for any of the experiments. When sampling, the lymph node of the mammary gland was removed, and the tissue was frozen immediately.

Bovine mammary tissue was collected from four multiparous Holstein dairy cows at mid-lactation. Biopsies were performed on the upper one-third of the posterior area of one udder using the method described by Farr *et al.* [42]. Approximately 500 mg of mammary tissue were collected, rinsed in 0.9% sterile saline solution, inspected to verify tissue homogeneity and then snap-frozen.

Both bovine and mouse mammary gland samples were stored at −80°C until RNA extraction.

### Tissue lysates and Argonaute-2 co-immunoprecipitation

Mouse mammary glands were homogenised individually in 1 mL lysis buffer (20 mM Tris HCl pH 7.5, 200 mM NaCl, 2.5 mM MgCl_2_, 0.5% Triton ×100, complete EDTA-free protease inhibitors (Roche) and 100 U/mL RNase OUT (Invitrogen)) per 100 mg tissue and stored in ice. Homogenates were centrifuged at 10,000 g, for 10 min. at 4°C. The supernatants were recovered, and kept at 4°C pending AGO2 co-immunoprecipitation. 50 µl Dynabeads® Protein G (Immunoprecipitation Kit- Dynabeads® Protein G; Cat. No. 100.07D; Invitrogen) per sample were prepared according to the manufacturer's protocol and 5 µg anti-AGO2 monoclonal antibody (Abnova, EIF2C2 monoclonal antibody (M01), clone 2E12-1C9; Cat. No. H00027161-M01), diluted in Ab Binding and Washing Buffers provided with the supplier's kit, were added to each aliquot of beads. Supernatants from mammary lysis were subsequently added to the beads and incubated overnight under constant rotation at 4°C. The beads were washed five times with 200 µL lysis buffer and finally resuspended in 90 µL lysis buffer supplemented with 10 µL 0.1 M DTT.

### RNA isolation

Total RNA were isolated from mouse tissue biopsies or from the samples obtained after AGO2 co-immunoprecipitation using the RNA NOW kit (Ozyme), with overnight precipitation so as to guarantee a maximum yield of small RNA. Bovine total RNA were prepared from about 50 mg mammary tissue using the Nucleospin® miRNA isolation kit (Macherey-Nagel Inc.) according to the manufacturer's instructions. The concentration and purity of total RNA were estimated by spectrophotometry (Nanodrop™, ND-1000) and its integrity was ascertained by migration on 2% agarose gel and analysed by displaying 28S and 18S rRNA. Bovine samples were pooled in equal amounts by pairs to obtain 10 µg RNA from each sample. Both mouse and bovine samples were precipitated using sodium acetate 3M, 96% ethanol and 5 mg/mL glycogen.

### Small library preparation and sequencing

Libraries were prepared simultaneously using the Illumina small RNA kit (Illumina) from GATC Biotech Company, according to the manufacturer's instructions. Briefly, the small RNA (below 500 nt) were isolated from total RNA using the mirVana miRNA isolation kit (Ambion) and then size-fractionated and purified on a denaturing 15% polyacrylamid gel, and stained with SYBR Green II. The small RNA fractions (19–29 nt) were collected by passive elution of the RNA from the gel, then precipitated with ethanol and dissolved in water. The small RNA thus isolated were poly(A)-tailed and an RNA adapter was ligated to the 5′-phosphate of the RNA. First-strand cDNA synthesis was performed using an oligo(dT)-adapter primer and M-MLV reverse transcriptase. The resulting cDNA were PCR-amplified to about 10–20 ng/µL using high fidelity DNA polymerase, over 18 to 26 cycles. The primers (sense: 5′- AATGATACGGCGACCACCGAGATCTACACTCTTTCCCTACACGACGCTCTTCCGATCT-3′ and antisense: 5′-CAAGCAGAAGACGGCATACGAGAT-Barcode-GTGACTGGAGTTCAGACGTGTGCTCTTCCGATCTTTTTTTTTTTTTTTTTTTTTTTTT-3′) used for the PCR amplification were designed for the purpose of TruSeq sequencing according to the instructions provided by Illumina. The barcode sequences attached to the 5′-end of the cDNA are listed in Table S1. The PCR products (total length of 165–175 bp) were analysed by capillary electrophoresis on a Shimadzu MultiNA microchip electrophoresis system and purified using the Agencourt AMPure XP kit (Beckman Coulter Genomics). Libraries were sequenced on an Illumina HiSeq 2000 by GATC Biotech Company, according to Solexa's sequencing method. All RNA sequencing data were subsequently deposited in the Gene Expression Omnibus (GEO): GSE53511.

### Sequencing data analyses and discovery of novel miRNA

After removing the poly-A stretches, data analyses were mainly performed using miRDeep2 software [43] as described in Figure S1. After filtering for their size (17–28 nt), the cleaned sequences were clustered into unique reads and then mapped to the mouse (GRCm38.71) and bovine (UMD3.1.71) reference genomes using the mapper.pl module from miRDeep2. Novel miRNA and precursors were identified using the miRDeep2 core module miRDeep2.pl (including the presence of reads corresponding to typical products of miRNA biogenesis, stability of the putative pre-miRNA hairpin and homology to previously identified miRNA). For both species, novel miRNA datasets were created by adding miRNA predicted with a miRDeep2 score >0 to known miRNA for the species (miRBase v20). The same operation was performed to create a new data set of precursors for both species. The quantifier.pl miRDeep2 module was then used to map the unique reads, new sets of miRNA and all known miRNA (miRBase v20) on the new sets of miRNA precursors. The quantification results generated by this module were then filtered with a custom perl script parse_miRDeep2_outputs.pl (https://mulcyber.toulouse.inra.fr/projects/bioinfoutils/) to eliminate any redundancy between known and putative novel miRNA.

### Analysis of differential expression

A differential expression analysis between mouse and AGO2-miRNomes was performed using R version 3.0.1 (R Development Core Team, 2013, http://www.R-project.org) with the Bioconductor DESeq2 package, version 1.0.17 [44]. DESeq2 utilises a negative binomial distribution to model read counts per miRNA and implements a method to normalise these counts. This normalisation procedure uses the library median of the ratios between the read count and the geometric mean of each gene as a scaling factor for each library. Fold changes were estimated using an empirical Bayes shrinkage procedure. This helps to moderate the large spread in fold changes for genes with low counts, while it has negligible effect on genes with high counts. Since hypothesis tests are performed for gene-by-gene differential analyses, the p-values obtained need to be adjusted to correct for multiple testing. However, procedures to adjust p-values in order to control the number of false positives found often lead to a loss of power to detect truly differentially expressed genes because of the large number of hypothesis tests performed. To reduce the impact of such procedures, the filtering method described by Rau *et al.* [45] was used to remove genes that appeared to generate an uninformative signal. This method identifies a filtering threshold that maximizes so-called filtering similarity among replicates. Tests for differential expression were only applied to genes whose maximal count across all four samples was higher than its threshold. This method was implemented under the Bioconductor HTSFilter package, version 1.0.0. [45]. The threshold value was found to be equal to 175. Applying this filtering reduced the 1,750 miRNA to just 299. The p-values were adjusted for multiple testing using the Benjamini and Hochberg method [46], and those with an adjusted p-value<0.05 were considered to be significant.

### miRNA targeted pathway analysis

miRNA targeted pathway analysis was performed using the computational application DIANA miRPath v2.0 [47], applied to the top 24 most highly expressed miRNA in mouse and bovine lactating mammary glands. miRNA targets were predicted with high accuracy based on DIANA-microT-CDS [48]. Targets of putative novel miRNA were predicted using TargetScanMouse Custom release 5.2 [5] and then their functional analysis was performed using DAVID Bioinformatics Tools [49], [50].

## Results and Discussion

### Characterisation of lactating mouse and bovine mammary gland miRNomes

**Figure 1:**
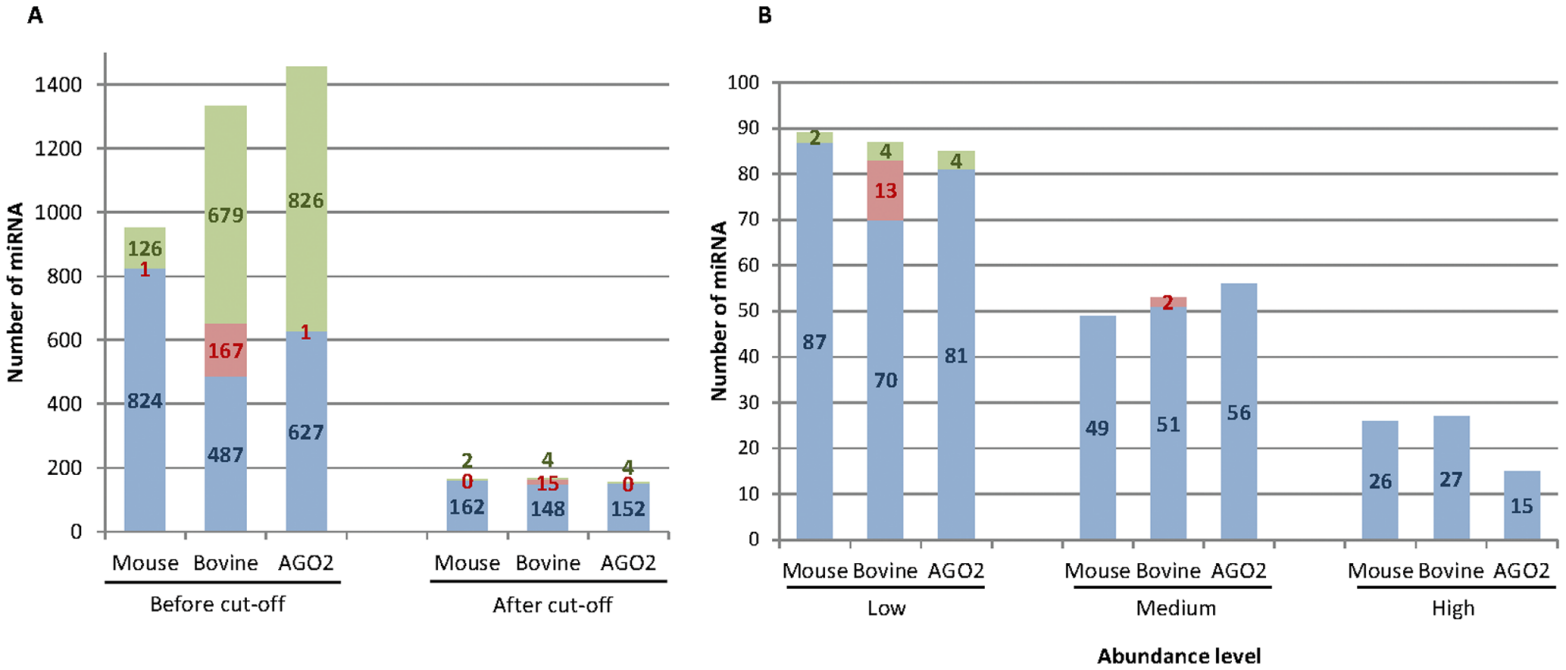
Characterisation of mouse and bovine lactating mammary gland libraries. A. miRNA identification in mouse and bovine lactating mammary glands. Unique sequences in mouse, bovine and AGO2-miRNomes were identified and classified in three categories according to their hits: (i) known miRNA in the species (blue), (ii) miRNA identified in other species (red) and (iii) predicted novel miRNA (green). Numbers of miRNA are presented before and after applying the 100 RPM cut-off point. **B. Distribution of miRNA according to their abundance in mouse and bovine lactating mammary glands.** The miRNA were classified in three categories: low, 100≤miRNA<1,000 RPM; medium, 1,000≤miRNA<10,000 RPM; and high, miRNA≥10,000 RPM.

Four libraries were constructed using small RNA isolated from lactating mouse (2) and bovine (2 pools of 2 cows) mammary glands and sequenced using Illumina/Solexa technology. More than 10 million reads were obtained from each library (Table 1). After removing the poly-A stretches, 9.26 and 10.51 million reads of 17–28 nt on average for the two samples of each species, were obtained from the mice and bovine libraries, respectively (Table 1). These clean and sized reads corresponded to 86.1% and 91.9% of mouse and bovine reads, respectively. They were mapped to the mouse (GRCm38.71) and bovine (UMD3.1.71) genomes and aligned against miRBase (version 20.0). 62,046 and 46,169 unique sequences were identified in mouse and bovine samples, respectively, corresponding to three categories based on their hits: (i) known miRNA in the species, corresponding to 824 and 487 in mouse and bovine, respectively; (ii) novel miRNA in the species, but also identified in other species, corresponding to 1 and 167 miRNA in mouse and bovine, respectively; and (iii) predicted novel miRNA corresponding to 126 and 679 miRNA in mouse and bovine, respectively (Figure 1A, Table S2). The quantity of predicted novel miRNA and of miRNA identified in other species but unknown in the sequenced species, was more important in bovine than in mouse and this could probably be explained by the smaller number of studies performed in bovine than in the mouse. However, the number of predicted novel mouse miRNA identified here (126) remained substantial. Our data also significantly increased knowledge of bovine miRNA and the number of miRBase entries. However, in both species, predicted novel miRNA and known miRNA in other species were represented by a small number of read counts in most of cases, and only a very small percentage of reads (0.9 to 0.1%), confirming that the use of NGS technology allowed results close to the complete miRNA profile. However, deeper sequencing would be able to reveal more low abundant miRNA.

**Table 1:** Summary of sequencing data lactating mouse, bovine and AGO2 mammary gland miRNA libraries.

	Mouse		Bovine		AGO2	
	Sample 1	Sample 2	Sample 1	Sample 2	Sample 1	Sample 2
Raw reads	10,782,945	10,738,221	10,847,425	12,034,673	12,940,967	13,643,425
Clean reads^a^	9,909,832	8,760,102	9,951,849	11,245,052	5,485,346	7,610,033
Sized reads^b^	9,817,286	8,708,817	9,877,580	11,149,432	5,307,791	7,368,804
All unique sequences corresponding to sized reads	128,806	90,569	97,136	111,437	55,616	305,201*
Mapped reads^c^	9,369,334	8,402,247	9,549,490	10,802,108	4,674,418	6,508,282
Unique sequences corresponding to mapped reads	72,653	51,438	40,750	51,587	26,843	230,688*

a poly-A stretches removed.

b 17–28 nt size filtering,reads used by miRDeep2 quantification process.

c reads with at least one and at most five reported alignments, used by the miRDeep2 prediction process.

* In AGO sample 2, many reads below 10 counts were present, subsequently discarded by miRDeep2 processes.

In a recent study, Mullokandov *et al.* [51] showed that miRNA representing less than 100 reads per million (RPM) are unlikely to be functionally relevant and they found that the majority of suppressed sensors correspond to miRNA or miRNA families expressed above 1,000 RPM. Only a small number of miRNA were expressed at a sufficient concentration to mediate sensor regulation.

Reads representing more than 100 RPM in at least one of the four libraries corresponded to 164 and 167 miRNA in mouse and bovine, respectively (Figure 1A). Application of this threshold resulted in the elimination of a significant number of miRNA, which accounted for only 0.5% and 0.6% of the reads in mouse and bovine, respectively. Mullokandov *et al.* [51] precised that as the targets of one miRNA are subject to regulation by all family members, the cumulative concentration of an entire miRNA family could be considered. By pooling miRNA sharing seeds, the number of different seed sequences reaching 1,000 RPM corresponds to 50 in each of the species (Table S3), which probably represent the major part of miRNA with suppressive activity.

In each miRNome, the miRNA could be classified as a function of their abundance (Figure 1B). Twenty six and 27 miRNA were highly expressed with more than 10,000 RPM, which constituted 78.1% and 76.6% of the total reads in mouse and bovine, respectively. Forty nine and 53 miRNA in mouse and bovine, respectively, were expressed at moderate levels of between 1,000 and 10,000 RPM. Eighty nine and 87 miRNA in mouse and bovine, respectively, were expressed at low levels of between 100 and 1,000 RPM. All predicted novel miRNA were expressed with a low abundance.

### Comparison of mouse and bovine lactating mammary gland miRNomes

One purpose of the present study was to evaluate miRNome conservation between mouse and bovine mammary glands during lactation. By comparing the two miRNomes, 123 miRNA were found to be present in both species (Table S2). Moreover, 41 (39 known and 2 predicted novel) miRNA were detected in mouse but not in bovine and 44 (40 known and 4 predicted novel) miRNA were detected in bovine, but not in mouse. The number of miRNA exclusive to one of these two species, in this study, appeared high, but more than 80% of them had a low expression level and represented fewer than 0.6% of the reads.

Deep sequencing can reliably profile miRNA abundance relative to total miRNA, and its normalisation enables comparison of the ranking of miRNA expression between different samples. The mouse and bovine miRNA abundance profiles were strongly conserved with a correlation of ranks of 0.72. However, few exceptions with different ranks of expression between species were observed. For example, *miR-146b-5p* and *miR-378a-3p* were highly expressed in mouse but they were present at a low level of expression in bovine; inversely, *miR-199b-5p*, *miR-423-5p* and *miR-193a-5p* were weakly expressed in mouse but highly in bovine (Table S2).

We can hypothesized that the miRNome differences observed between mouse and bovine could be the existence of inherent species differences [52]; for example differences in the relative proportion of secretory epithelium and adipocytes in cow *versus* mouse. To attenuate this point, the characterisation was performed on mammary gland from lactating animals, at which time the organ is mainly composed of mammary epithelial cells in both species. Moreover, cattle have been selected for sustained milk production, and this could explain some of the miRNome differences observed *versus* the mouse data. Although the data were obtained using the same procedure and the same bioinformatics and biostatistical analyses during this study, we could not exclude the possibility that such differences might be due to technical biases [53], [54], [55] or to the RNA isolation methods used.

**Figure 2:**
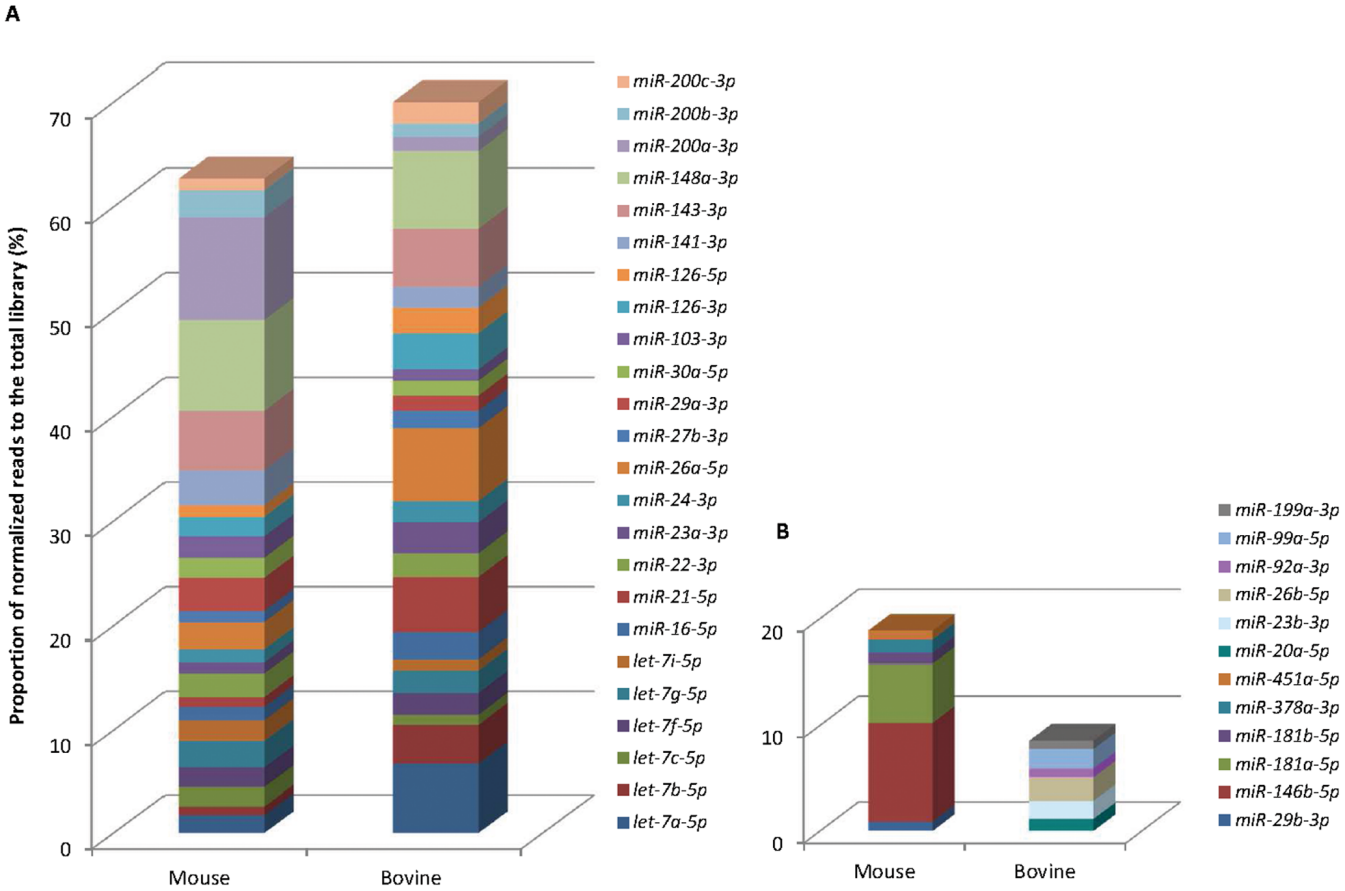
The top 30 most highly expressed miRNA in mouse and bovine lactating mammary glands. Representation of the percentage of normalised reads in the total library of the 24 miRNA common to the mouse and bovine species (**A**) and of the six miRNA present in only one species (**B**).

Amongst the 30 most expressed miRNA in each miRNome, six miRNA were present only in the mouse top 30, and six others in the bovine top 30 (Figure 2). Among them, four miRNA (*miR-29b-3p*, *miR-181a-5p*, *miR-181b-5* and *miR-451a-5p*) and five miRNA (*miR-20a-5p*, *miR-23b-3p*, *miR-26b-5p*, *miR-99a-5p* and *miR-199a-3p*) in the mouse and bovine, respectively, were expressed in the other species with moderate (over 10,000 reads) to high abundance (Table S2). 24 miRNA were common to both species (Figure 2) and, in order to estimate their impact to the lactation process, their presence was compared with the 30 miRNA most highly expressed from different organs, from published data obtained with the high-throughput sequencing technology (Table S4, Figure S2). Fifteen of them were present in the top 30 of several tissues such as brain, muscle, liver, lung or endometrium (in bold in Table S4). Most of these 15 had already been defined as abundant and ubiquitously expressed miRNA by Landgraf *et al.* [56] in their foundation work on the mammalian miRNA expression atlas. These 15 miRNA may be important regulators of fundamental and common biological processes and we can hypothesise that they are involved in basic processes of mammary gland biology but not necessarily in its specific functions, such as milk component synthesis or secretion. For example, six are members of the *let-7* gene family known to be ubiquitously expressed and involved in regulating cell proliferation and differentiation (reviewed in [57]). Among the 24 common miRNA, seven other miRNA (*miR-16-5p*, *miR-23a-3p*, *miR-126-5p*, *miR-126-3p*, and three members of the *miR-200* family (*miR-200a-3p*, *miR-200b-3p*, *miR-200c-3p*)) were mainly detected in the top 30 of different epithelial tissues, such as kidney, lung or endometrium (Table S4, Figure S2), suggesting that they could be involved in physiological processes linked to epithelial cell functions. Indeed, the importance of the *miR-200* family to normal mammary gland development was recently reported by Chang *et al.* [28] and Nagaoka *et al.* [58]. Moreover two miRNA (*miR-22-3p* and *miR-141-3p)* were only detected among the 24 common miRNA of our studies on the mammary gland, thus suggesting the value of further investigating their role. The expression of the seven miRNA detected in the top 30 of epithelial tissues and the two miRNA expressed only in mammary gland (*miR-22-3p* and *miR-141-3p*) were compared with those on published non-lactating mammary gland miRNomes generated from several species (bovine [40], caprine [38], [39], [41] and human [59]) using the same NGS technology. Six of them (*miR-126-5p*, *miR-16-5p*, and members of the *miR-200* family (*miR-141-3p*, *miR-200a-3p*, *miR-200b-3p*, *miR-200c-3p*)) were not present amongst the 30 highly abundant miRNA in non-lactating miRNomes. It can therefore be suggested that these six miRNA may have functions linked to the lactation process. It would be interesting to investigate their function further in order to confirm this hypothesis.

### Characterisation of AGO2-miRNome in lactating mouse mammary gland

In mouse lactating mammary gland, miRNA associated with AGO2 protein (AGO2-miRNome) were exhaustive described on small RNA isolated by co-immunoprecipitation using anti-AGO2 antibody (Figure S3) and deep sequencing. Two libraries from two animals were produced and an average of more than 13 million reads was obtained (Table 1). The data were analysed as described previously. After cleaning and sizing, 6.34 million reads of 17–28 nt were obtained. Around 50% of these reads were eliminated. This percentage was high, and in line with the results obtained by immunoprecipitation using the anti-AGO protein approach [60]. After normalisation and applying a cut-off point at 100 RPM, 152 known miRNA and four predicted novel miRNA were identified (Figure 1A). The miRNA were distributed between different levels of abundance (Figure 1B), which showed that 15 of them were highly expressed.

The mouse miRNome from the total RNA was then compared with the AGO2-miRNome. Before the 100 RPM cut-off point was applied, the lists of the miRNA were not identical. In fact, some miRNA identified in the mouse mammary gland miRNome were not found in the AGO2-miRNome and conversely (Table S2). The difference occurred on miRNA with a low reads: all miRNA identified after application of the cut-off point in one miRNome were present in the other miRNome but some of them were expressed below 100 RPM (Table S5). Some of this difference could be explained by a lower concentration of total RNA in the immunoprecipitated samples than that obtained from the organ, which impacted the composition of the libraries.

**Figure 3:**
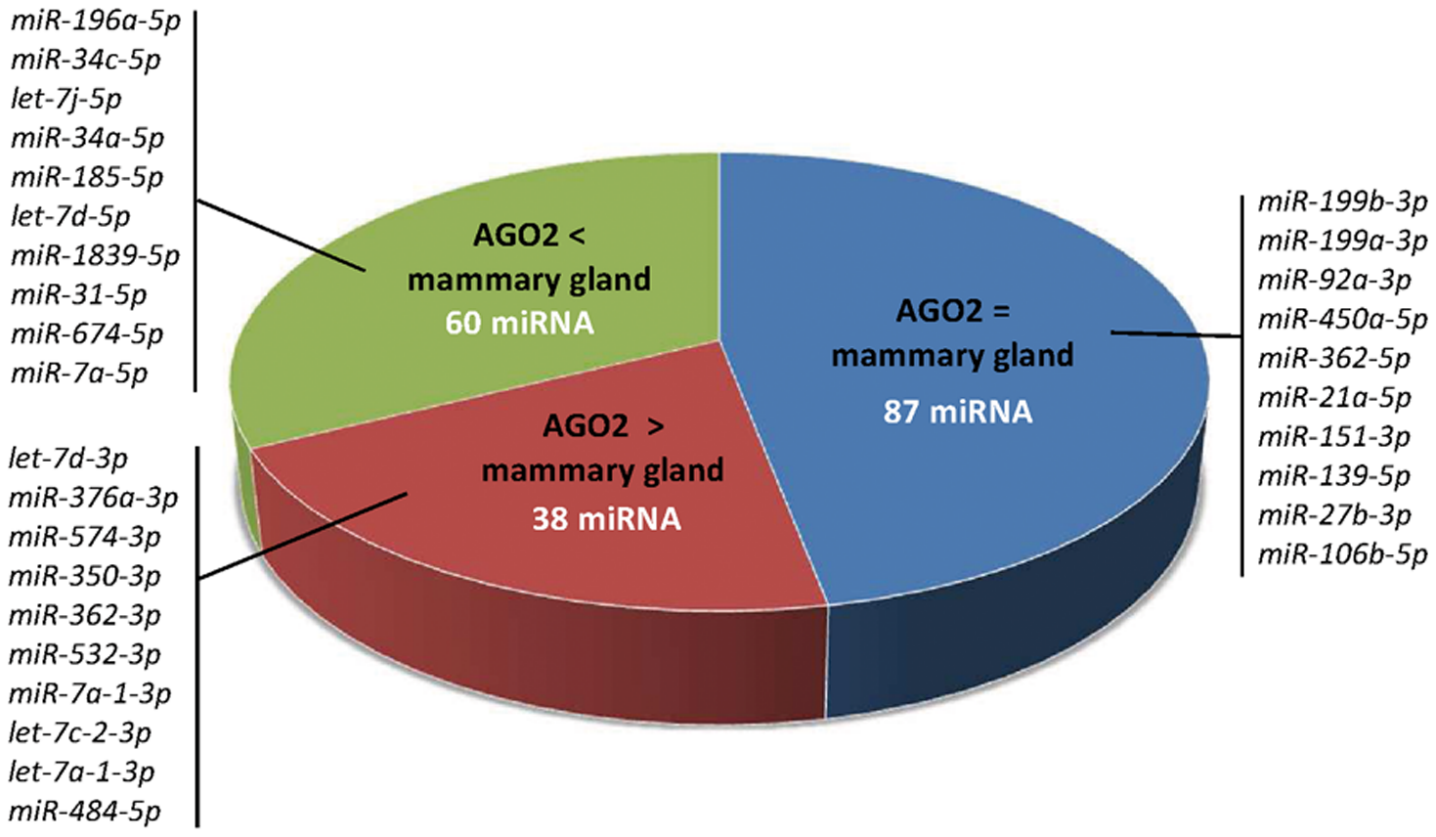
Characterisation of mouse AGO2-RISC loading. Proportion of miRNA expressed with an equivalent ( = ) or a different (> or <) abundance in AGO2-RISC and mammary gland miRNomes. A few representative examples are presented for each category and the complete data are listed in Table S5.

For each miRNA, AGO2-RISC loading was characterised by the comparing of the read counts after normalisation of the whole and AGO2-miRNomes. For example, miRNA expressed with the same number of counts in the two miRNomes were considered to be fully loaded in AGO2-RISC. The miRNA were classified according to their percentage loading in AGO2-RISC (Table S5). Eighty seven miRNA were expressed at the same levels in AGO2- and whole miRNomes (Figure 3). Sixty miRNA were significantly less loaded (or depleted) in AGO2-RISC (p-value<0.05), including seven that were loaded at less than 10% (*miR-674-5p*, *miR-34c-5p*, *miR-196a-5p*, *let-7j-5p*, *miR-34a-5p*, *let-7d-5p* and *miR-185-5p*). Thirty eight were present with more counts in the AGO2- compared with the whole miRNomes. As observed by Burroughs *et al.* [18], our data suggests that each miRNA would not be loaded in an equivalent way in the AGO-RISC. In our study, miRNA enriched or depleted in AGO2-RISC were not the same as those seen in human THP-1 cells [18], showing that the enrichment and depletion processes are dependent on the biological context.

### Identification of putative novel microRNA

**Figure 4:**
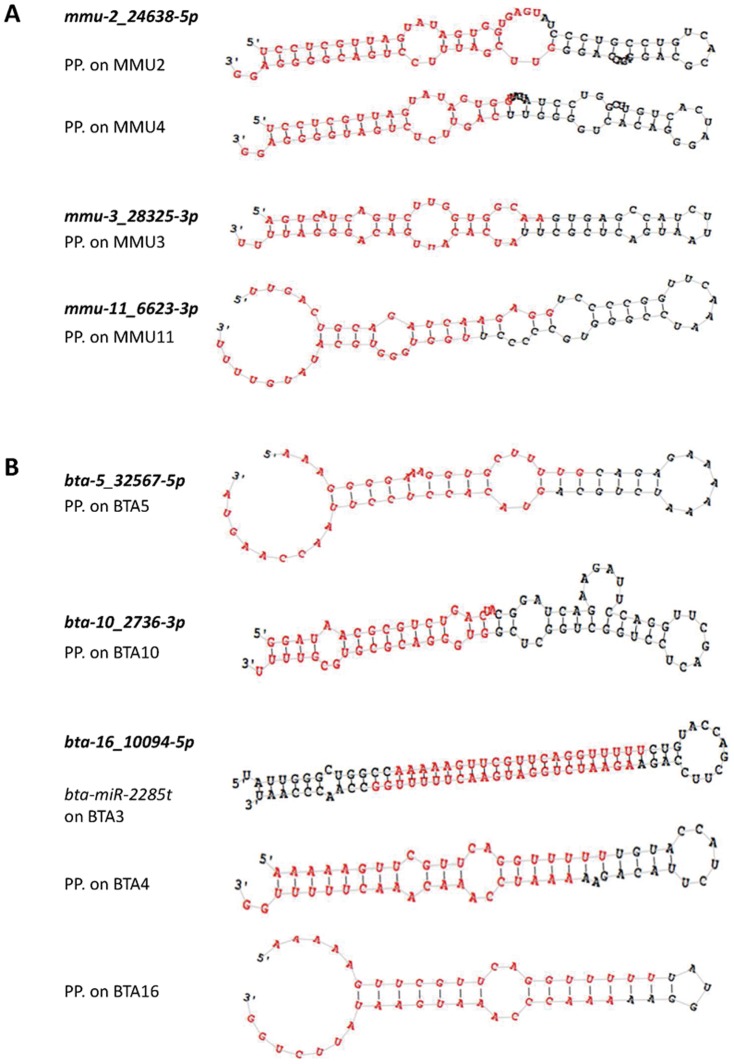
Predicted precursor structures of mouse (A) and bovine (B) predicted novel miRNA. Secondary structures of predicted precursor (PP) hairpins corresponding to seven predicted novel miRNA identified in this study using miRDeep2 software. The predicted miRNA mature sequences -5p and -3p are highlighted in red. Several putative precursors were located for three of them.

The NGS technology also constitutes a powerful tool to identify novel miRNA. Using the miRDeep2 package, 126 and 679 novel miRNA were predicted in the mouse and bovine miRNomes, respectively. However, after a cut-off point was applied at 100 RPM, only two and four predicted novel miRNA (Table 2, Figure 4) were retained in the mouse and bovine, respectively. They were expressed at low levels which could explain why they had not been described previously. Moreover, four predicted novel miRNA (after the cut-off at 100 RPM) were detected in the AGO2-miRNome. As mentioned by Burroughs *et al.* [18], the presence of mRNA and small RNA other than miRNA, in immunoprecipited samples, is important. For this reason, filters for different contaminants following the miRDeep2 analysis were added. Two of the four novel miRNA initially predicted from AGO2 samples corresponded to an mRNA and an snRNA. The other two predicted novel miRNA were present in the mouse miRNome, but only one was retained because the other was expressed below 100 RPM.

**Table 2:** Predicted novel mouse and bovine miRNA.

miRNome	Name	Sequence with predicted seed	Read counts	miRDeep2 score	Example miRBase miRNA with the same seed
Mouse	*mmu-3_28325-3p*	A**UCACAUU**GACAGGGAUUUU	1,165	3.5	*mmu-miR-23a-3p*
	*mmu-11_6623-3p*	U**GGUGGGU**GCAUAUGUUUU	3,204	5.1	*aly-miR294a-3p*
Bovine	*bta-5_32567-5p*	A**AAGGGGA**AAGGUGCUUUUG	7,442	3.5	*cin-miR-4005b-3p*
	*bta-10_2736-3p*	G**UGGGACG**CGUGCGUUUU	7,790	1.2	*gga-miR-1670*
	*bta-16_10094-5p*	A**AAAAGUU**CGUUCAGGUUUUU	2,047	4.2	*bta-miR-2284j*
	*bta-16_10667-5p*	C**AUUGGUG**GUUCAGUGGU	1,628	4.8	*zma-miR171c-5p*
AGO2	*mmu-2_24638-5p*	U**CCUCGUU**AGUAUAGUGGUGAGU	727	1.1	*nve-miR-2033-3p*
	*mmu-3_28325-3p*	A**UCACAUU**GACAGGGAUUUU	2,054	3.5	*mmu-miR-23a-3p*

In bold type: seed.

If we considered the predicted novel miRNA present in at least one miRNome after application of the cut-off point at 100 RPM, we were able to detect three and four different predicted novel miRNA in the mouse and bovine, respectively. Moreover, three of them (*mmu-2_24638-5p*, *bta-16_10094-5p*, *bta-16_10667-5p*) were localised in several genomic positions, thus corresponding to several potential new precursors (Figure 4).

The evolution of novel miRNA families is intimately linked to that of novel cell types associated with highly specialised biological functions [61]. They may provide an important substrate for the emergence of new regulatory activities, with a higher degree of tissue specificity and lower level of expression [62]. Because the mammary gland is an evolutionarily a recent organ, and lactation is one of the most remarkable products of evolution, the predicted novel miRNA described in this study could correspond to tissue-specific miRNA of importance to the functioning of this organ.

Among the seven novel miRNA predicted in this study, five contained a seed that already exists in known miRNA. Two novel miRNA displayed significant homology to those known in the species (*mmu-3_28325-3p* to *mmu-miR-23a-3p* and *bta-16_10094-5p* to *bta-miR-2284j*). miRNA *mmu-3_28325-3p* was also present in the AGO2-miRNome, which is a further argument to support the idea that this miRNA is not a false positive miRNA. Moreover, *miR-23a* has been described as being involved in the processes of EMT [63] or apoptosis [64]. As these processes are important in the mammary gland biology, the role of *mmu-3_28325-3p*, it would be interesting to investigate this feature further. Furthermore, the miRDeep2 data results specified that miRNA *bta-16_10094-5p* miRNA is perfectly aligned to the 5′ position of the known precursor *bta-miR-2285t* (miRBase reference MI0022348; data not shown), for which only the mature 3p is currently described. Thereby, this predicted novel bovine miRNA appears to be a member of the *bta-miR-2284/2285* bovine-specific family, which has more than 40 members spanning the entire bovine genome [65], [66].

### miRNA target gene predictions and KEGG pathway analysis

**Figure 5:**
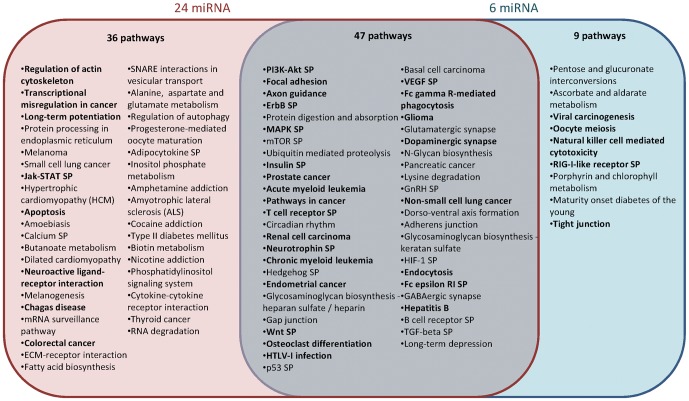
Pathways targeted by the miRNA highly expressed in mouse and bovine mammary glands. KEGG function annotations, ordered by ascending p-values (<0.05, Table S6), for the 24 miRNA in the top 30 common to mouse and bovine and the six miRNA highly expressed in lactating but not non-lactating mammary glands. Biological processes targeted by 23 or 24/24 miRNA and 6/6 miRNA are indicated in bold type. SP, signaling pathway.

To investigate the functional role of the 24 common miRNA highly expressed in bovine and mouse mammary glands during lactation, target gene predictions were performed based on miRNA/mRNA interactions using Diana-microT v3.0. The predicted target genes were classified according to KEGG function annotations which allowed the identification of 83 biological processes with a p-value<0.05 (Figure 5 and Table S6). The most enriched signalling pathways were found to be the PI3K-Akt, MAPK, insulin, Wnt and calcium pathways. Focal adhesion, axon guidance and ubiquitin mediated proteolysis were identified as being some of the most targeted functions by these 24 miRNA (Figure 5). Genes involved in cellular structure (gap junction, focal adhesion and adherens junction) also seemed to be preferentially targeted by these miRNA.

As for the miRNA highly expressed during lactation but not during non-lactating stages, the same types of analysis were performed on the six miRNA identified (*miR-126-5p*, *miR-16-5p*, *miR-141-3p*, *miR-200a-3p*, *miR-200b-3p*, *miR-200c-3p*). KEGG annotation identified 56 biological processes with a p-value<0.05, including 47 pathways common to the previous analysis. Some pathways, involved in important functions in the mammary gland (protein processing in the endoplasmic reticulum, the Jak-STAT signalling pathway, apoptosis, the calcium signaling pathway, ECM-receptor interaction or cytokine-cytokine receptor interaction) were predicted as targeted by the 24 miRNA, but they were not detected using the six miRNA found to be highly expressed during lactation but not during non-lactation stages. Moreover, five pathways significantly targeted by these six miRNA only were identified and included the tight junction pathway involved in cellular structure. Some genes in this pathway important to mammary gland biology, such as *Pten*, *Ocln* and *Gnai*, were identified as being targeted by more than one of the six miRNA.

Highly expressed miRNA seemed to be involved in pathways important to mammary gland biology, and better characterisation of their expression profiles in terms of physiological status will enable a refinement of their function.

Regarding the seven predicted novel miRNA, their gene targets and functions were also predicted. In all cases, some important pathways (Table S7) in mammary gland function, such as the Wnt signalling pathway or apoptosis, were identified. Several genes involved in “mammary gland development” or “lactation” (*Chuk*, *Ccnd1*, *Met*, *Prlr*, *Stat5b*, *Elf5*, *Tgfbr2*) were targeted by *mmu-3_28325-3p*. Moreover, other genes involved in “mammary gland morphogenesis” (*Notch1*, *Sox9*, *Areg*, *Btrc*, *Esr1*, *Igf1*, *Igf1r*, *Nr3c1*, *Pax6*, *Trp63*) or “mammary gland epithelial cell proliferation” (*Sox9*, *Areg*, *Btrc*, *Col8a1*, *Esr1*) were targeted by *bta-16_10094-5p*. These results suggest that the predicted novel miRNA *mmu-3_28325-3* and *bta-16_10094-5p* are likely to play a key role in the biology of the mammary gland.

## Conclusion

This study constitutes the first report of multispecies (mouse and bovine) mammary miRNomes in the context of lactation and the identification of some species-specific miRNA involved in this fundamental biological function. The miRNomes were completed by characterisation of an exhaustive list of miRNA present in mouse AGO2-RISC. The miRNomes generated will be used as a reference for further studies during which the impact of breeding on these miRNomes will be evaluated. The data obtained here are also important and essential to the next stage of this research, which will focus on the functional characterization of miRNA in the mammary gland during lactation.

## Supporting Information

Figure S1 **Flow charts of data processing steps.** Flow charts for (**A**) the creation of a new miRNA reference dataset, (**B**) the quantification of all sample and (**C**) the filtering and cleaning of raw outputs. For each step (in dotted borders) the input, work flow and output are shown. Files are presented in rectangular boxes; processes are presented in rounded boxes. MirDeep2 internal processes and output files are in orange. Original reference files are in blue, while new files produced by our process (in black) are in green. The file formats are: .fa, fasta; .arf, arf mapping format; .mrd, miRDeep2 text output; .csv, csv spread-sheet.
(TIF)

Figure S2 **Expression of 3 miRNA of the top 30 most highly expressed miRNA in epithelial and non-epithelial tissues confirming NGS data.** Relative expression of *miR-200a-3p* (TaqMan® ID 000502, Applied Biosystems), *miR-26a-5p* (TaqMan® ID 000405) and *let-7c-5p* (TaqMan® ID 000379) were determined by RT-qPCR, on Mastercycler RealPlex 4 (Eppendorf®), in epithelial (mammary gland at lactation day-12 (MG) and kidney (K)) and non-epithelial (brain (B) and liver (L)) mouse tissue samples. miRNA expression were normalized to U6 expression (TaqMan ® ID 001973). Values are means ± S.E. (n = 3 technical repetitions on 3 different individuals).
(TIF)

Figure S3 **AGO2 enrichment after immunoprecipitation (IP).** Anti-Ago2 Western-blot on an 10% Bis-acrylamide Tris gel (EIF2C2 monoclonal antibody (M01), clone 2E12-1C9, Cat. No. H00027161-M01, Abnova). Endogenous mouse AGO2 protein weight: 97 kDa. 1, 2 and 3: mammary gland lysates of 3 samples (input fractions, 50 µg per lane); 4 and 5: IP fractions of 2 samples (<5 µg per lane).
(TIF)

Table S1 **Barcode sequences attached to the 5′-end of the cDNA used for the libraries preparation and sequencing.**
(DOCX)

Table S2 **Quantification (normalized sequencing reads) of known miRNA in the species (A) or in other species (B) and predicted novel miRNA (C) in mouse, AGO2 and bovine miRNomes of lactating mammary gland.**
(DOCX)

Table S3 **miRNA sharing identical seeds reaching cumulative 1,000 RPM threshold in lactating mouse and bovine mammary miRNomes.**
(DOCX)

Table S4 **Tissues expression of the 24 common miRNA of the top 30 most expressed miRNA in bovine and mouse mammary glands.** All the data considered for this comparison have been obtained by NGS technology on organs. **References** : Brain [67]; Endometrium [68]; Lung [69]; Muscle [70]; Liver [71]; Kidney [72]; Bladder [73]; Stomach [74]; Heart [75].
(DOCX)

Table S5 **AGO2-RISC loading (p-value<0.05) of the 185 miRNA expressed in the mouse miRNome and AGO2-miRNome**: (**A**) miRNA showing statistical equivalent abundances into both miRNomes, (**B**) miRNA significantly enriched in AGO2-RISC and (**C**) miRNA significantly less loaded in AGO2-RISC.
(DOCX)

Table S6 **Pathways targeted by miRNA highly expressed in mouse and bovine mammary gland.**
(DOCX)

Table S7 **Major pathways targeted by the predicted novel miRNA highly expressed in mouse and bovine mammary gland.**
(DOCX)
