# Supplementary figures and images for: Characterisation and Comparison of Lactating Mouse and Bovine Mammary Gland miRNomes
Source: PLoS One. 2014 Mar 21;9(3):e91938. doi: 10.1371/journal.pone.0091938 (PMC3962357; doi:10.1371/journal.pone.0091938)

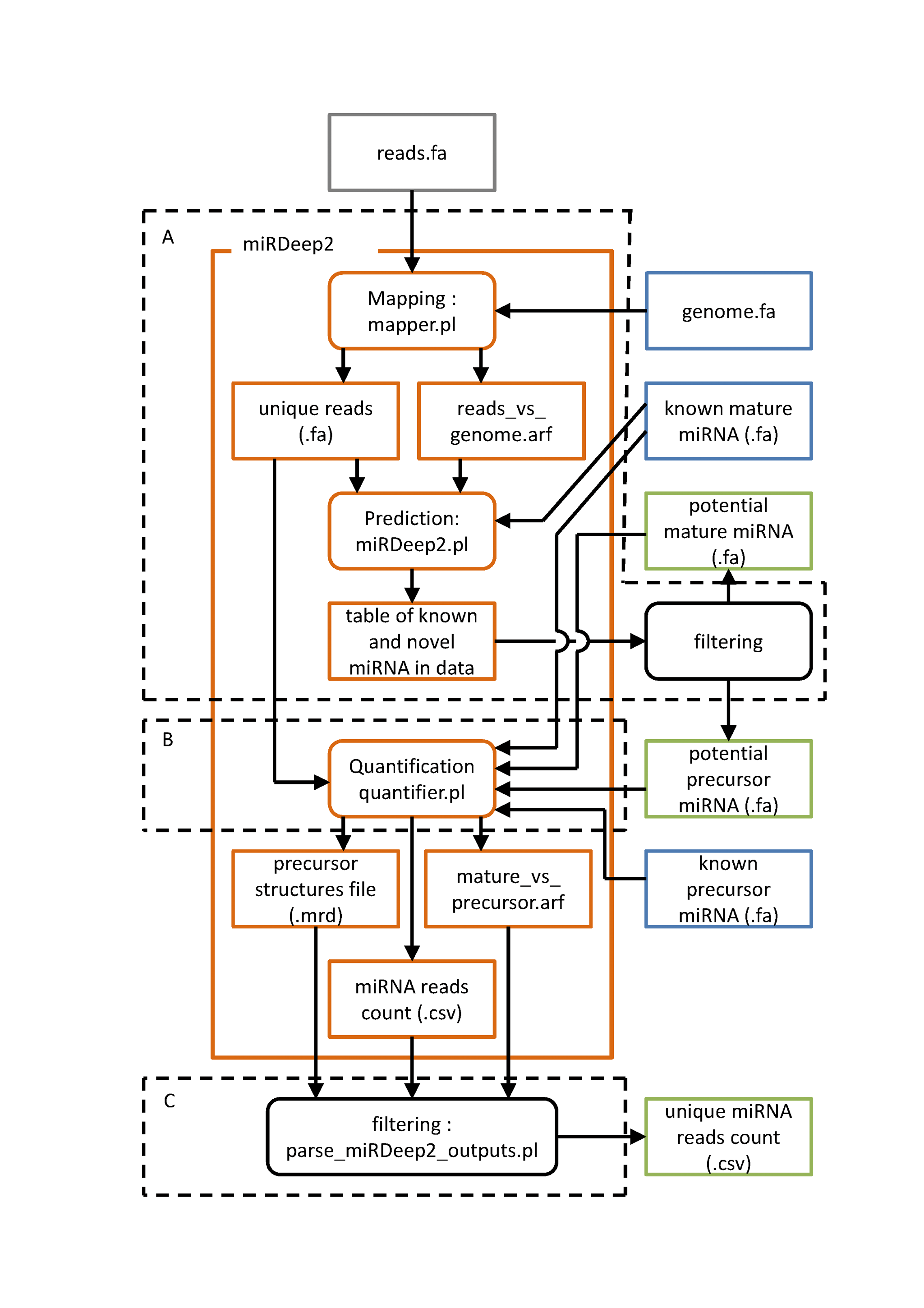

Supplement: Figure S1 — Flow charts of data processing steps. Flow charts for (A) the creation of a new miRNA reference dataset, (B) the quantification of all sample and (C) the filtering and cleaning of raw outputs. For each step (in dotted borders) the input, work flow and output are shown. Files are presented in rectangular boxes; processes are presented in rounded boxes. MirDeep2 internal processes and output files are in orange. Original reference files are in blue, while new files produced by our process (in black) are in green. The file formats are: .fa, fasta; .arf, arf mapping format; .mrd, miRDeep2 text output; .csv, csv spread-sheet. (TIF) [file pone.0091938.s001.tif]

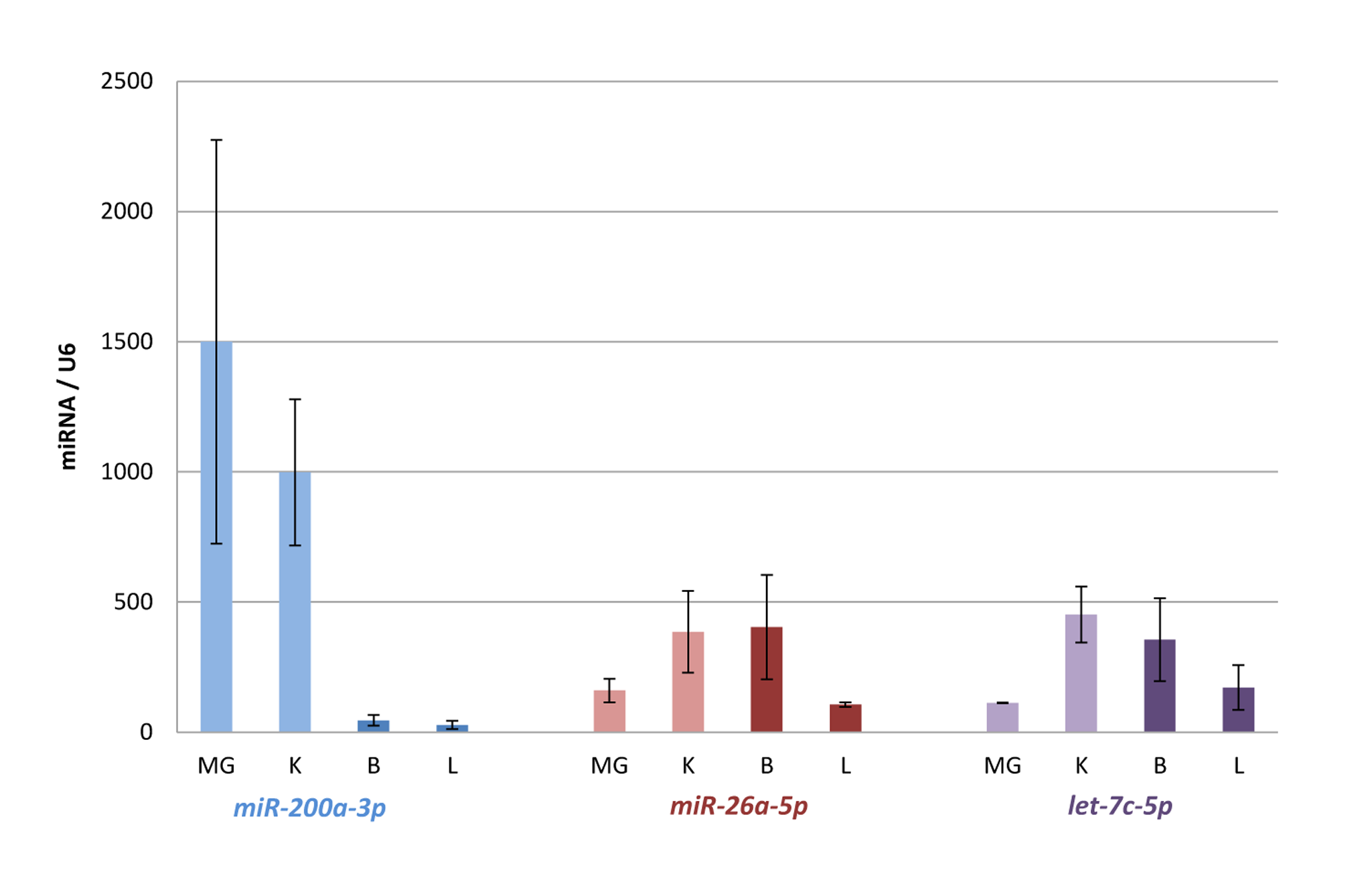

Supplement: Figure S2 — Expression of 3 miRNA of the top 30 most highly expressed miRNA in epithelial and non-epithelial tissues confirming NGS data. Relative expression of miR-200a-3p (TaqMan® ID 000502, Applied Biosystems), miR-26a-5p (TaqMan® ID 000405) and let-7c-5p (TaqMan® ID 000379) were determined by RT-qPCR, on Mastercycler RealPlex 4 (Eppendorf®), in epithelial (mammary gland at lactation day-12 (MG) and kidney (K)) and non-epithelial (brain (B) and liver (L)) mouse tissue samples. miRNA expression were normalized to U6 expression (TaqMan ® ID 001973). Values are means ± S.E. (n = 3 technical repetitions on 3 different individuals). (TIF) [file pone.0091938.s002.tif]

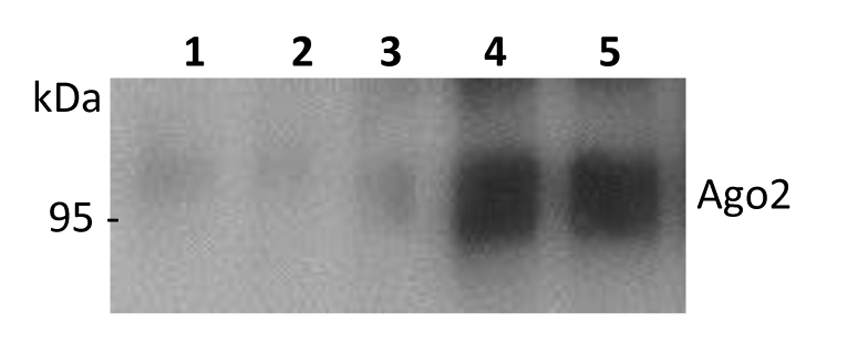

Supplement: Figure S3 — AGO2 enrichment after immunoprecipitation (IP). Anti-Ago2 Western-blot on an 10% Bis-acrylamide Tris gel (EIF2C2 monoclonal antibody (M01), clone 2E12-1C9, Cat. No. H00027161-M01, Abnova). Endogenous mouse AGO2 protein weight: 97 kDa. 1, 2 and 3: mammary gland lysates of 3 samples (input fractions, 50 µg per lane); 4 and 5: IP fractions of 2 samples (<5 µg per lane). (TIF) [file pone.0091938.s003.tif]
