# Supplementary material for: Characterisation and Comparison of Lactating Mouse and Bovine Mammary Gland miRNomes
Source: PLoS One. 2014 Mar 21;9(3):e91938. doi: 10.1371/journal.pone.0091938 (PMC3962357; doi:10.1371/journal.pone.0091938)
Supplement: Table S1 — Barcode sequences attached to the 5′-end of the cDNA used for the libraries preparation and sequencing. (DOCX) [file pone.0091938.s004.docx]

**Table S1. Barcode sequences attached to the 5’-end of the cDNA used for the libraries preparation and sequencing.**

| **Sample** | **Barcode sequence** |
| --- | --- |
| Mouse mammary sample 1 | ACAGTG |
| Mouse mammary sample 2 | GCCAAT |
| Mouse mammary AGO2 co-immunoprecipitation sample 1 | CAGATC |
| Mouse mammary AGO2 co-immunoprecipitation sample 2 | ACTTGA |
| Bovine mammary sample 1 | CGATGT |
| Bovine mammary sample 2 | TGACCA |
