# Supplementary material for: Characterisation and Comparison of Lactating Mouse and Bovine Mammary Gland miRNomes
Source: PLoS One. 2014 Mar 21;9(3):e91938. doi: 10.1371/journal.pone.0091938 (PMC3962357; doi:10.1371/journal.pone.0091938)
Supplement: Table S2 — Quantification (normalized sequencing reads) of known miRNA in the species (A) or in other species (B) and predicted novel miRNA (C) in mouse, AGO2 and bovine miRNomes of lactating mammary gland. (DOCX) [file pone.0091938.s005.docx]

**Supplementary table S2. Quantification (normalized sequencing reads) of known miRNA in the species (A) or in other species (B) and predicted novel miRNA (C) in mouse, AGO2 and bovine miRNomes of lactating mammary gland.**

**A.**

| **miRNA** | **Mouse** | **AGO2** | **miRNA** | **Bovine** |
| --- | --- | --- | --- | --- |
| *mmu-let-7a-1-3p* | 349.1 | 2335.6 | *bta-let-7a-3p* | 451.5 |
| ***mmu-let-7a-5p*** | 103889.8 | 19303.9 | ***bta-let-7a-5p*** | 674346.4 |
| *mmu-let-7b-3p* | 10.6 | 198.5 |  |  |
| ***mmu-let-7b-5p*** | 50453.5 | 13756.2 | ***bta-let-7b-5p*** | 376636.6 |
| *mmu-let-7c-1-3p* | 2.5 | 7.3 |  |  |
| *mmu-let-7c-2-3p* | 349.1 | 2335.6 |  |  |
| ***mmu-let-7c-5p*** | 112994.0 | 19028.6 | ***bta-let-7c-5p*** | 96913.8 |
| *mmu-let-7d-3p* | 247.3 | 44619.2 |  |  |
| ***mmu-let-7d-5p*** | 40754.7 | 4115.0 | ***bta-let-7d-5p*** | 31774.8 |
| *mmu-let-7e-3p* | 33.1 | 162.0 |  |  |
| ***mmu-let-7e-5p*** | 5065.8 | 1197.5 | ***bta-let-7e-5p*** | 18569.9 |
| *mmu-let-7f-1-3p* | 56.7 | 335.2 |  |  |
| *mmu-let-7f-2-3p* | 15.0 | 100.1 |  |  |
| ***mmu-let-7f-5p*** | 114475.1 | 25729.5 | ***bta-let-7f-5p*** | 208127.5 |
| *mmu-let-7g-3p* | 4.6 | 10.6 |  |  |
| ***mmu-let-7g-5p*** | 153630.6 | 33222.9 | ***bta-let-7g-5p*** | 211395.7 |
| *mmu-let-7i-3p* | 61.1 | 591.3 |  |  |
| ***mmu-let-7i-5p*** | 120240.8 | 22812.7 | ***bta-let-7i-5p*** | 108548.5 |
| *mmu-let-7j-5p* | 905.7 | 77.1 |  |  |
| *mmu-let-7k-5p* | 7.0 | 1.4 |  |  |
| *mmu-miR-1a-1-5p* | 0.4 | 10.0 |  |  |
| *mmu-miR-1a-2-5p* | 0.0 | 0.7 |  |  |
| ***mmu-miR-1a-3p*** | 1105.0 | 150.6 | ***bta-miR-1-3p*** | 7424.9 |
| *mmu-miR-1b-3p* | 33.5 | 2.7 |  |  |
| *mmu-miR-7a-1-3p* | 157.4 | 1373.4 |  |  |
| ***mmu-miR-7a-5p*** | 3758.3 | 518.2 | ***bta-miR-7-5p*** | 1290.5 |
| *mmu-miR-7b-5p* | 1.9 | 0.0 |  |  |
| *mmu-miR-9-3p* | 13.4 | 4.0 | *bta-miR-9-3p* | 29.0 |
| *mmu-miR-9-5p* | 159.8 | 88.5 | *bta-miR-9-5p* | 194.2 |
| *mmu-miR-10a-3p* | 183.1 | 39.4 |  |  |
| ***mmu-miR-10a-5p*** | 14578.7 | 11650.6 | ***bta-miR-10a-5p*** | 5192.2 |
| *mmu-miR-10b-3p* | 288.0 | 48.9 |  |  |
| ***mmu-miR-10b-5p*** | 30769.4 | 38783.4 | ***bta-miR-10b-5p*** | 71518.0 |
| *mmu-miR-15a-3p* | 0.3 | 0.0 |  |  |
| ***mmu-miR-15a-5p*** | 25185.7 | 12950.3 | ***bta-miR-15a-5p*** | 62838.5 |
| *mmu-miR-15b-3p* | 60.8 | 255.5 |  |  |
| ***mmu-miR-15b-5p*** | 8451.3 | 6454.8 | ***bta-miR-15b-5p*** | 12572.3 |
| *mmu-miR-16-1-3p* | 2.6 | 12.0 |  |  |
| *mmu-miR-16-2-3p* | 47.7 | 244.2 |  |  |
| ***mmu-miR-16-5p*** | 75608.7 | 34978.6 | ***bta-miR-16a-5p*** | 262324.7 |
|  |  |  | *bta-miR-16b-5p* | 59412.8 |
| ***mmu-miR-17-3p*** | 1216.2 | 732.0 | ***bta-miR-17-3p*** | 1713.1 |
| ***mmu-miR-17-5p*** | 21831.3 | 14585.6 | ***bta-miR-17-5p*** | 51918.3 |
| *mmu-miR-18a-3p* | 27.1 | 34.4 |  |  |
| ***mmu-miR-18a-5p*** | 12340.5 | 5268.5 | ***bta-miR-18a-5p*** | 7234.7 |
| *mmu-miR-18b-5p* | 58.1 | 58.8 | *bta-miR-18b-5p* | 1925.0 |
| *mmu-miR-19a-3p* | 176.1 | 320.6 | *bta-miR-19a-3p* | 96.0 |
| *mmu-miR-19b-1-5p* | 23.5 | 49.1 |  |  |
| *mmu-miR-19b-2-5p* | 0.3 | 0.0 |  |  |
| *mmu-miR-19b-3p* | 836.3 | 1162.8 | *bta-miR-19b-3p* | 588.0 |
| *mmu-miR-20a-3p* | 185.2 | 279.1 |  |  |
| ***mmu-miR-20a-5p*** | 46952.5 | 32010.0 | ***bta-miR-20a-5p*** | 113575.3 |
| *mmu-miR-20b-3p* | 2.9 | 29.5 |  |  |
| *mmu-miR-20b-5p* | 146.1 | 109.2 | *bta-miR-20b-5p* | 2938.1 |
| *mmu-miR-21a-3p* | 40.2 | 170.6 | *bta-miR-21-3p* | 44.5 |
| ***mmu-miR-21a-5p*** | 57088.7 | 58307.5 | ***bta-miR-21-5p*** | 526527.6 |
| *mmu-miR-21b-5p* | 0.0 | 1.4 |  |  |
| ***mmu-miR-22-3p*** | 134846.7 | 61592.6 | ***bta-miR-22-3p*** | 226540.0 |
| ***mmu-miR-22-5p*** | 4510.3 | 799.9 | ***bta-miR-22-5p*** | 7886.0 |
| ***mmu-miR-23a-3p*** | 64183.7 | 79217.4 | ***bta-miR-23a-3p*** | 295975.4 |
| *mmu-miR-23a-5p* | 1.1 | 4.0 |  |  |
| ***mmu-miR-23b-3p*** | 31177.6 | 41771.6 | ***bta-miR-23b-3p*** | 170170.7 |
| *mmu-miR-23b-5p* | 9.3 | 1.4 | *bta-miR-23b-5p* | 11.0 |
| *mmu-miR-24-1-5p* | 137.2 | 368.0 | *bta-miR-24-5p* | 409.4 |
| *mmu-miR-24-2-5p* | 211.9 | 170.1 |  |  |
| ***mmu-miR-24-3p*** | 74230.4 | 18652.5 | ***bta-miR-24-3p*** | 201878.2 |
| ***mmu-miR-25-3p*** | 13280.5 | 11115.6 | ***bta-miR-25-3p*** | 50941.5 |
| *mmu-miR-25-5p* | 1.7 | 0.0 |  |  |
| *mmu-miR-26a-1-3p* | 4.4 | 0.7 |  |  |
| *mmu-miR-26a-2-3p* | 8.9 | 12.1 |  |  |
| ***mmu-miR-26a-5p*** | 155545.9 | 96940.3 | ***bta-miR-26a-5p*** | 697260.3 |
| *mmu-miR-26b-3p* | 5.1 | 0.0 |  |  |
| ***mmu-miR-26b-5p*** | 46515.3 | 19881.8 | ***bta-miR-26b-5p*** | 223189.3 |
| ***mmu-miR-27a-3p*** | 43781.8 | 90185.4 | ***bta-miR-27a-3p*** | 72032.3 |
| *mmu-miR-27a-5p* | 52.3 | 13.3 | *bta-miR-27a-5p* | 91.0 |
| ***mmu-miR-27b-3p*** | 63609.1 | 64795.8 | ***bta-miR-27b-3p*** | 166537.5 |
| *mmu-miR-27b-5p* | 109.6 | 73.8 |  |  |
| *mmu-miR-28a-3p* | 1301.1 | 709.4 |  |  |
| ***mmu-miR-28a-5p*** | 3336.8 | 837.6 | ***bta-miR-28-5p*** | 15761.6 |
| *mmu-miR-28b-5p* | 5.2 | 0.0 |  |  |
| *mmu-miR-28c-3p* | 2.7 | 1.3 |  |  |
| ***mmu-miR-29a-3p*** | 191065.6 | 418945.1 | ***bta-miR-29a-3p*** | 144962.9 |
| ***mmu-miR-29a-5p*** | 749.8 | 678.7 |  |  |
| *mmu-miR-29b-1-5p* | 300.8 | 101.2 |  |  |
| *mmu-miR-29b-2-5p* | 102.4 | 8.8 |  |  |
| ***mmu-miR-29b-3p*** | 52210.4 | 85543.1 | ***bta-miR-29b-3p*** | 67593.7 |
| ***mmu-miR-29c-3p*** | 4268.8 | 12636.6 | ***bta-miR-29c-3p*** | 23996.5 |
| *mmu-miR-29c-5p* | 53.1 | 20.2 |  |  |
|  |  |  | *bta-miR-29d-3p* | 100.0 |
|  |  |  | *bta-miR-29d-5p* | 465.2 |
|  |  |  | *bta-miR-29e-3p* | 36.5 |
| ***mmu-miR-30a-3p*** | 7171.6 | 5174.4 |  |  |
| ***mmu-miR-30a-5p*** | 113792.7 | 231486.8 | ***bta-miR-30a-5p*** | 143087.5 |
| *mmu-miR-30b-3p* | 179.5 | 15.3 | *bta-miR-30b-3p* | 454.0 |
| ***mmu-miR-30b-5p*** | 2130.5 | 10075.7 | ***bta-miR-30b-5p*** | 1068.0 |
| *mmu-miR-30c-1-3p* | 53.1 | 10.9 |  |  |
| *mmu-miR-30c-2-3p* | 502.5 | 71.8 |  |  |
| *mmu-miR-30c-5p* | 3148.0 | 10196.1 | *bta-miR-30c-5p* | 508.9 |
| *mmu-miR-30d-3p* | 281.1 | 370.0 |  |  |
| ***mmu-miR-30d-5p*** | 28518.1 | 56538.6 | ***bta-miR-30d-5p*** | 40616.2 |
| ***mmu-miR-30e-3p*** | 2479.3 | 3238.1 |  |  |
| ***mmu-miR-30e-5p*** | 34157.2 | 40470.5 | ***bta-miR-30e-5p*** | 20113.8 |
|  |  |  | *bta-miR-30f-5p* | 129.7 |
| *mmu-miR-31-3p* | 7.6 | 18.6 |  |  |
| ***mmu-miR-31-5p*** | 2875.5 | 383.2 | ***bta-miR-31-5p*** | 6849.0 |
| *mmu-miR-32-3p* | 139.9 | 47.4 |  |  |
| *mmu-miR-32-5p* | 291.0 | 1359.0 | *bta-miR-32-5p* | 1288.1 |
| *mmu-miR-33-3p* | 62.9 | 63.8 |  |  |
| *mmu-miR-33-5p* | 380.5 | 2625.0 | *bta-miR-33a-5p* | 101.1 |
|  |  |  | *bta-miR-33b-5p* | 18.1 |
| *mmu-miR-34a-3p* | 4.2 | 50.0 |  |  |
| ***mmu-miR-34a-5p*** | 10577.5 | 994.3 | ***bta-miR-34a-5p*** | 25629.4 |
| *mmu-miR-34b-3p* | 11.6 | 146.0 | *bta-miR-34b-5p* | 37.0 |
| *mmu-miR-34b-5p* | 755.7 | 359.9 |  |  |
| *mmu-miR-34c-3p* | 17.0 | 66.2 |  |  |
| *mmu-miR-34c-5p* | 651.0 | 31.9 | *bta-miR-34c-5p* | 22.0 |
| *mmu-miR-92a-1-5p* | 1.5 | 0.0 |  |  |
| ***mmu-miR-92a-3p*** | 7549.1 | 7269.6 | ***bta-miR-92a-3p*** | 85082.1 |
| *mmu-miR-92b-3p* | 34.2 | 65.5 | *bta-miR-92b-3p* | 661.7 |
| *mmu-miR-93-3p* | 189.7 | 379.0 |  |  |
| ***mmu-miR-93-5p*** | 14846.0 | 9853.3 | ***bta-miR-93-5p*** | 21862.9 |
|  |  |  | *bta-miR-935-3p* | 4.0 |
|  |  |  | *bta-miR-95-3p* | 471.9 |
| *mmu-miR-96-3p* | 32.9 | 2.7 |  |  |
| *mmu-miR-96-5p* | 4732.1 | 3457.9 | *bta-miR-96-5p* | 926.7 |
| *mmu-miR-98-3p* | 2.5 | 20.0 |  |  |
| ***mmu-miR-98-5p*** | 3443.4 | 444.5 | ***bta-miR-98-5p*** | 10115.9 |
| *mmu-miR-99a-3p* | 111.0 | 429.5 | *bta-miR-99a-3p* | 1359.3 |
| ***mmu-miR-99a-5p*** | 22940.3 | 45648.0 | ***bta-miR-99a-5p*** | 183184.8 |
| *mmu-miR-99b-3p* | 134.2 | 132.1 |  |  |
| ***mmu-miR-99b-5p*** | 1216.7 | 5784.4 | ***bta-miR-99b-5p*** | 3347.7 |
| *mmu-miR-100-3p* | 13.5 | 6.1 |  |  |
| ***mmu-miR-100-5p*** | 1145.1 | 1908.2 | ***bta-miR-100-5p*** | 14491.7 |
| ***mmu-miR-101a-3p*** | 7465.8 | 10308.4 | ***bta-miR-101-3p*** | 7111.8 |
| *mmu-miR-101a-5p* | 24.7 | 24.2 |  |  |
| *mmu-miR-101b-3p* | 17698.5 | 12017.6 |  |  |
| *mmu-miR-101c-3p* | 259.5 | 546.5 |  |  |
| *mmu-miR-103-1-5p* | 3.4 | 0.0 |  |  |
| *mmu-miR-103-2-5p* | 9.1 | 0.0 |  |  |
| ***mmu-miR-103-3p*** | 123506.3 | 26391.1 | ***bta-miR-103-3p*** | 108035.2 |
|  |  |  | *bta-miR-105a-5p* | 2.0 |
| *mmu-miR-106a-5p* | 86.6 | 88.2 | *bta-miR-106a-5p* | 5431.9 |
| *mmu-miR-106b-3p* | 489.5 | 476.0 |  |  |
| ***mmu-miR-106b-5p*** | 17605.9 | 18048.6 | ***bta-miR-106b-5p*** | 38306.8 |
| ***mmu-miR-107-3p*** | 28224.2 | 12995.1 | ***bta-miR-107-3p*** | 15074.6 |
| *mmu-miR-107-5p* | 5.9 | 6.0 |  |  |
| *mmu-miR-122-5p* | 14.0 | 4.0 | *bta-miR-122-5p* | 24.0 |
| *mmu-miR-124-3p* | 244.3 | 70.9 | *bta-miR-124a-3p* | 9.0 |
|  |  |  | *bta-miR-124b-3p* | 9.0 |
| *mmu-miR-125a-3p* | 197.1 | 169.5 |  |  |
| ***mmu-miR-125a-5p*** | 8060.3 | 15629.3 | ***bta-miR-125a-5p*** | 49299.7 |
| *mmu-miR-125b-1-3p* | 11.0 | 2.1 |  |  |
| *mmu-miR-125b-2-3p* | 229.4 | 337.9 |  |  |
| ***mmu-miR-125b-5p*** | 3625.3 | 6453.7 | ***bta-miR-125b-5p*** | 47760.9 |
| ***mmu-miR-126a-3p*** | 110649.8 | 43061.4 | ***bta-miR-126-3p*** | 344477.8 |
| ***mmu-miR-126a-5p*** | 65905.6 | 142400.5 | ***bta-miR-126-5p*** | 245702.9 |
| *mmu-miR-126b-3p* | 10.9 | 2.7 |  |  |
| *mmu-miR-126b-5p* | 12.8 | 22.0 |  |  |
| *mmu-miR-127-3p* | 101.9 | 49.4 | *bta-miR-127-3p* | 439.0 |
| *mmu-miR-127-5p* | 85.0 | 84.1 |  |  |
| *mmu-miR-128-1-5p* | 0.3 | 0.0 |  |  |
| ***mmu-miR-128-3p*** | 788.0 | 489.3 | ***bta-miR-128-3p*** | 3121.0 |
| *mmu-miR-129-5p* | 18.4 | 0.0 | *bta-miR-129-5p* | 12.0 |
| ***mmu-miR-130a-3p*** | 7595.9 | 6093.5 | ***bta-miR-130a-3p*** | 8029.2 |
| *mmu-miR-130a-5p* | 0.4 | 10.0 |  |  |
| *mmu-miR-130b-3p* | 335.1 | 127.0 | *bta-miR-130b-3p* | 269.0 |
| *mmu-miR-130b-5p* | 1.0 | 2.0 |  |  |
| *mmu-miR-132-3p* | 300.5 | 297.4 | *bta-miR-132-3p* | 490.1 |
| *mmu-miR-132-5p* | 64.2 | 168.6 |  |  |
| *mmu-miR-133a-3p* | 27.1 | 168.1 | *bta-miR-133a-3p* | 123.9 |
| *mmu-miR-133a-5p* | 1.8 | 5.4 |  |  |
| *mmu-miR-133b-3p* | 0.0 | 31.4 |  |  |
| *mmu-miR-134-3p* | 0.0 | 0.7 |  |  |
| *mmu-miR-134-5p* | 40.3 | 8.0 | *bta-miR-134-5p* | 20.0 |
| *mmu-miR-135a-5p* | 11.0 | 3.5 | *bta-miR-135a-5p* | 1566.6 |
| *mmu-miR-135b-5p* | 2.1 | 3.3 | *bta-miR-135b-5p* | 0.5 |
| *mmu-miR-136-3p* | 330.9 | 1940.2 |  |  |
| *mmu-miR-136-5p* | 7.7 | 178.3 | *bta-miR-136-5p* | 4.5 |
| *mmu-miR-137-3p* | 21.0 | 3.3 | *bta-miR-137-3p* | 2.0 |
| *mmu-miR-138-1-3p* | 1.1 | 0.0 |  |  |
| *mmu-miR-138-2-3p* | 3.2 | 0.8 |  |  |
| *mmu-miR-138-5p* | 180.7 | 31.9 | *bta-miR-138-5p* | 96.4 |
| *mmu-miR-139-3p* | 11.9 | 2.0 |  |  |
| ***mmu-miR-139-5p*** | 3345.7 | 3441.7 | ***bta-miR-139-5p*** | 29852.6 |
| ***mmu-miR-140-3p*** | 3538.1 | 3819.2 | ***bta-miR-140-3p*** | 6920.3 |
| ***mmu-miR-140-5p*** | 2000.5 | 645.1 |  |  |
| ***mmu-miR-141-3p*** | 200291.1 | 488136.7 | ***bta-miR-141-3p*** | 197906.4 |
| *mmu-miR-141-5p* | 1865.4 | 578.6 |  |  |
| ***mmu-miR-142-3p*** | 4232.0 | 3573.3 | ***bta-miR-142-3p*** | 2862.3 |
| ***mmu-miR-142-5p*** | 2761.6 | 6356.8 | ***bta-miR-142-5p*** | 6492.9 |
| ***mmu-miR-143-3p*** | 341010.8 | 188338.5 | ***bta-miR-143-3p*** | 555650.4 |
| *mmu-miR-143-5p* | 13.2 | 2.7 |  |  |
| *mmu-miR-144-3p* | 6400.3 | 32439.1 | *bta-miR-144-3p* | 142.9 |
| *mmu-miR-144-5p* | 133.3 | 214.9 |  |  |
| ***mmu-miR-145a-3p*** | 2829.2 | 1707.5 |  |  |
| *mmu-miR-145a-5p* | 12089.9 | 14497.0 | *bta-miR-145-5p* | 62417.3 |
| *mmu-miR-145b-5p* | 2.0 | 2.3 |  |  |
| ***mmu-miR-146a-5p*** | 6423.3 | 3127.6 | ***bta-miR-146a-5p*** | 9071.2 |
| *mmu-miR-146b-3p* | 2861.7 | 3854.4 |  |  |
| ***mmu-miR-146b-5p*** | 560809.1 | 351747.6 | ***bta-miR-146b-5p*** | 6309.3 |
| *mmu-miR-147-3p* | 32.8 | 0.7 | *bta-miR-147-3p* | 201.5 |
| ***mmu-miR-148a-3p*** | 520302.1 | 615075.4 | ***bta-miR-148a-3p*** | 743742.9 |
| *mmu-miR-148a-5p* | 49.3 | 20.7 |  |  |
| ***mmu-miR-148b-3p*** | 9114.0 | 6758.3 | ***bta-miR-148b-3p*** | 10890.0 |
| *mmu-miR-148b-5p* | 15.8 | 34.9 |  |  |
| *mmu-miR-149-5p* | 244.5 | 567.8 | *bta-miR-149-5p* | 420.2 |
| *mmu-miR-150-3p* | 8.8 | 0.0 |  |  |
| ***mmu-miR-150-5p*** | 1530.3 | 4514.0 | ***bta-miR-150-5p*** | 16948.9 |
| ***mmu-miR-151-3p*** | 1952.6 | 1991.0 | ***bta-miR-151-3p*** | 17603.5 |
| ***mmu-miR-151-5p*** | 12070.4 | 4084.4 | ***bta-miR-151-5p*** | 69381.4 |
| ***mmu-miR-152-3p*** | 27430.4 | 13349.2 | ***bta-miR-152-3p*** | 19114.4 |
| *mmu-miR-152-5p* | 6.1 | 6.7 |  |  |
| *mmu-miR-153-3p* | 2.9 | 0.0 | *bta-miR-153-3p* | 6.5 |
| *mmu-miR-154-3p* | 32.9 | 33.9 |  |  |
| *mmu-miR-154-5p* | 439.8 | 279.7 | *bta-miR-154a-5p* | 21.5 |
|  |  |  | *bta-miR-154b-5p* | 99.0 |
|  |  |  | *bta-miR-154c-3p* | 151.3 |
| *mmu-miR-155-3p* | 0.0 | 4.0 |  |  |
| *mmu-miR-155-5p* | 231.9 | 72.9 | *bta-miR-155-5p* | 2122.3 |
| *mmu-miR-181a-1-3p* | 3276.1 | 8611.5 |  |  |
| *mmu-miR-181a-2-3p* | 0.3 | 0.7 |  |  |
| ***mmu-miR-181a-5p*** | 330854.1 | 233629.4 | ***bta-miR-181a-5p*** | 66374.6 |
| *mmu-miR-181b-1-3p* | 33.1 | 114.0 |  |  |
| *mmu-miR-181b-2-3p* | 36.6 | 278.9 |  |  |
| ***mmu-miR-181b-5p*** | 61253.1 | 24340.5 | ***bta-miR-181b-5p*** | 12224.1 |
| *mmu-miR-181c-3p* | 81.6 | 250.5 |  |  |
| *mmu-miR-181c-5p* | 3680.7 | 5627.2 | *bta-miR-181c-5p* | 491.6 |
| *mmu-miR-181d-3p* | 1.9 | 2.0 |  |  |
| *mmu-miR-181d-5p* | 1258.6 | 712.6 | *bta-miR-181d-5p* | 295.9 |
| *mmu-miR-182-3p* | 1.1 | 7.3 |  |  |
| ***mmu-miR-182-5p*** | 11461.5 | 4078.6 | ***bta-miR-182-5p*** | 5247.4 |
| *mmu-miR-183-3p* | 402.3 | 404.9 |  |  |
| ***mmu-miR-183-5p*** | 4409.2 | 1493.4 | ***bta-miR-183-5p*** | 1368.4 |
| *mmu-miR-184-3p* | 0.7 | 0.0 |  |  |
| *mmu-miR-185-3p* | 2.8 | 2.7 |  |  |
| ***mmu-miR-185-5p*** | 2294.8 | 235.7 | ***bta-miR-185-5p*** | 8359.9 |
| *mmu-miR-186-3p* | 83.5 | 47.9 |  |  |
| ***mmu-miR-186-5p*** | 5460.7 | 13230.6 | ***bta-miR-186-5p*** | 73141.1 |
| *mmu-miR-187-3p* | 1087.9 | 364.3 | *bta-miR-187-3p* | 13.4 |
| *mmu-miR-187-5p* | 4.6 | 0.0 |  |  |
| *mmu-miR-188-3p* | 46.9 | 125.1 |  |  |
| *mmu-miR-188-5p* | 196.9 | 541.6 | *bta-miR-188-5p* | 75.1 |
| *mmu-miR-190a-3p* | 7.8 | 318.7 |  |  |
| ***mmu-miR-190a-5p*** | 16519.3 | 2770.4 | ***bta-miR-190a-5p*** | 13930.7 |
| *mmu-miR-190b-3p* | 0.0 | 2.0 |  |  |
| *mmu-miR-190b-5p* | 190.3 | 37.1 | *bta-miR-190b-5p* | 139.5 |
| *mmu-miR-191-3p* | 51.1 | 72.8 |  |  |
| ***mmu-miR-191-5p*** | 11352.9 | 4597.6 | ***bta-miR-191-5p*** | 48477.7 |
| *mmu-miR-192-3p* | 3.0 | 2.0 |  |  |
| *mmu-miR-192-5p* | 260.0 | 367.6 | *bta-miR-192-5p* | 408.8 |
| *mmu-miR-193a-3p* | 37.6 | 262.1 | *bta-miR-193a-3p* | 10.5 |
| ***mmu-miR-193a-5p*** | 619.6 | 92.3 | ***bta-miR-193a-5p*** | 9614.2 |
| *mmu-miR-193b-3p* | 24.8 | 196.3 | *bta-miR-193b-3p* | 7.0 |
| *mmu-miR-193b-5p* | 13.9 | 0.0 |  |  |
| *mmu-miR-194-1-3p* | 0.7 | 0.0 |  |  |
| *mmu-miR-194-2-3p* | 7.6 | 0.0 |  |  |
| ***mmu-miR-194-5p*** | 5068.0 | 6582.4 | ***bta-miR-194-5p*** | 7063.4 |
| *mmu-miR-195a-3p* | 37.0 | 47.0 |  |  |
| ***mmu-miR-195a-5p*** | 7952.9 | 2900.8 | ***bta-miR-195-5p*** | 69528.2 |
| *mmu-miR-196a-2-3p* | 0.0 | 0.7 |  |  |
| ***mmu-miR-196a-5p*** | 749.5 | 38.9 | ***bta-miR-196a-5p*** | 2066.7 |
| *mmu-miR-196b-3p* | 0.0 | 4.0 |  |  |
| ***mmu-miR-196b-5p*** | 801.5 | 246.5 | ***bta-miR-196b-5p*** | 2033.6 |
|  |  |  | *bta-miR-197-3p* | 3.5 |
| ***mmu-miR-199a-3p*** | 22758.5 | 22630.9 | ***bta-miR-199a-3p*** | 79918.3 |
| ***mmu-miR-199a-5p*** | 1706.8 | 4838.5 | ***bta-miR-199a-5p*** | 2720.2 |
| *mmu-miR-199b-3p* | 22758.5 | 22630.9 |  |  |
| ***mmu-miR-199b-5p*** | 783.4 | 2310.2 | ***bta-miR-199b-5p*** | 22812.7 |
|  |  |  | *bta-miR-199c-3p* | 8577.0 |
| ***mmu-miR-200a-3p*** | 587080.6 | 1671001.5 | ***bta-miR-200a-3p*** | 135026.2 |
| *mmu-miR-200a-5p* | 876.3 | 1493.7 |  |  |
| ***mmu-miR-200b-3p*** | 154731.7 | 55107.7 | ***bta-miR-200b-3p*** | 127126.2 |
| ***mmu-miR-200b-5p*** | 2150.4 | 1420.8 |  |  |
| ***mmu-miR-200c-3p*** | 67117.7 | 19844.8 | ***bta-miR-200c-3p*** | 274578.7 |
| *mmu-miR-200c-5p* | 91.7 | 33.3 |  |  |
| *mmu-miR-201-5p* | 2.1 | 0.0 |  |  |
| *mmu-miR-202-5p* | 0.0 | 2.0 |  |  |
| *mmu-miR-203-3p* ***^m^*** | 8630.8 | 4610.1 |  |  |
| *mmu-miR-203-5p* | 35.3 | 7.4 |  |  |
| *mmu-miR-204-3p* | 1.5 | 0.8 |  |  |
| *mmu-miR-204-5p* | 18.2 | 47.9 | *bta-miR-204-5p* | 142.7 |
| *mmu-miR-205-3p* | 11.7 | 2.7 |  |  |
| ***mmu-miR-205-5p*** | 4575.1 | 5264.4 | ***bta-miR-205-5p*** | 42717.1 |
| *mmu-miR-206-3p* | 2.1 | 1.4 | *bta-miR-206-3p* | 12.0 |
| *mmu-miR-208a-3p* | 1.1 | 2.0 |  |  |
| *mmu-miR-208b-3p* | 1.6 | 0.7 |  |  |
| ***mmu-miR-210-3p*** | 17432.6 | 4354.8 | ***bta-miR-210-3p*** | 4091.9 |
| *mmu-miR-210-5p* | 0.4 | 11.3 |  |  |
|  |  |  | *bta-miR-211-5p* | 0.5 |
| *mmu-miR-212-3p* | 7.3 | 3.3 |  |  |
| *mmu-miR-212-5p* | 2.3 | 0.0 | *bta-miR-212-5p* | 2.0 |
| *mmu-miR-2137-3p* | 8.3 | 17.8 |  |  |
| ***mmu-miR-214-3p*** | 1154.9 | 442.0 | ***bta-miR-214-3p*** | 4473.7 |
| *mmu-miR-214-5p* | 6.1 | 7.3 |  |  |
| *mmu-miR-215-3p* | 0.7 | 0.0 |  |  |
| *mmu-miR-215-5p* | 27.1 | 12.1 | *bta-miR-215-5p* | 149.3 |
| *mmu-miR-216a-5p* | 0.0 | 2.0 |  |  |
|  |  |  | *bta-miR-216b-5p* | 1.5 |
| *mmu-miR-217-5p* | 0.4 | 0.0 | *bta-miR-217-5p* | 1.5 |
| *mmu-miR-218-2-3p* | 0.7 | 0.0 |  |  |
| ***mmu-miR-218-5p*** | 737.7 | 350.3 | ***bta-miR-218-5p*** | 2887.2 |
| *mmu-miR-219a-1-3p* | 20.5 | 4.0 | *bta-miR-219-3p* | 2.0 |
| *mmu-miR-219a-2-3p* | 0.6 | 0.0 |  |  |
| *mmu-miR-219a-5p* | 5.2 | 79.9 |  |  |
| *mmu-miR-219b-3p* | 0.4 | 2.0 |  |  |
| *mmu-miR-219c-3p* | 0.4 | 0.0 |  |  |
| *mmu-miR-219c-5p* | 0.7 | 0.0 |  |  |
| ***mmu-miR-221-3p*** | 31853.6 | 15461.3 | ***bta-miR-221-3p*** | 14973.0 |
| *mmu-miR-221-5p* | 235.3 | 115.2 |  |  |
| ***mmu-miR-222-3p*** | 3012.7 | 1626.3 | ***bta-miR-222-3p*** | 1518.9 |
| *mmu-miR-222-5p* | 5.8 | 2.7 |  |  |
| *mmu-miR-223-3p* | 1508.4 | 2579.0 | *bta-miR-223-3p* | 961.2 |
| *mmu-miR-223-5p* | 3.7 | 2.7 |  |  |
| *mmu-miR-224-3p* | 2.3 | 0.0 |  |  |
| *mmu-miR-224-5p* | 78.9 | 16.0 | *bta-miR-224-5p* | 2689.2 |
| *mmu-miR-291a-3p* | 0.4 | 5.3 |  |  |
| *mmu-miR-294-3p* | 0.7 | 0.0 |  |  |
| *mmu-miR-296-3p* | 6.1 | 4.7 | *bta-miR-296-3p* | 154.7 |
| *mmu-miR-296-5p* | 0.4 | 0.0 | *bta-miR-296-5p* | 10.0 |
| *mmu-miR-297a-3p* | 1.3 | 10.0 |  |  |
| *mmu-miR-297a-5p* | 65.1 | 15.4 |  |  |
| *mmu-miR-297b-3p* | 1.3 | 10.0 |  |  |
| *mmu-miR-297b-5p* | 13.4 | 2.7 |  |  |
| *mmu-miR-297c-3p* | 1.3 | 10.0 |  |  |
| *mmu-miR-297c-5p* | 23.8 | 12.6 |  |  |
| *mmu-miR-298-3p* | 0.0 | 5.3 |  |  |
| *mmu-miR-298-5p* | 11.6 | 2.7 |  |  |
| *mmu-miR-299a-3p* | 43.7 | 12.6 |  |  |
| *mmu-miR-299a-5p* | 64.1 | 53.7 | *bta-miR-299-5p* | 437.6 |
| *mmu-miR-299b-3p* | 0.3 | 0.0 |  |  |
| *mmu-miR-299b-5p* | 0.3 | 0.0 |  |  |
| *mmu-miR-300-3p* | 208.1 | 332.1 |  |  |
| *mmu-miR-300-5p* | 23.1 | 57.1 |  |  |
| ***mmu-miR-301a-3p*** | 2060.8 | 1487.4 | ***bta-miR-301a-3p*** | 1098.5 |
| *mmu-miR-301a-5p* | 0.0 | 2.7 |  |  |
| *mmu-miR-301b-3p* | 59.9 | 204.8 | *bta-miR-301b-3p* | 3.0 |
|  |  |  | *bta-miR-302a-3p* | 4.5 |
|  |  |  | *bta-miR-302b-3p* | 5.0 |
|  |  |  | *bta-miR-302c-3p* | 0.5 |
| *mmu-miR-302d-3p* | 0.0 | 1.3 | *bta-miR-302d-3p* | 4.5 |
| ***mmu-miR-320-3p*** | 5813.6 | 1611.7 | ***bta-miR-320a-3p*** | 34632.3 |
|  |  |  | *bta-miR-320b-3p* | 4.5 |
| *mmu-miR-322-3p* | 321.9 | 288.8 |  |  |
| *mmu-miR-322-5p* | 11240.4 | 10538.7 |  |  |
| *mmu-miR-323-3p* | 3.4 | 0.7 | *bta-miR-323-3p* | 31.4 |
| *mmu-miR-324-3p* | 41.9 | 41.6 |  |  |
| ***mmu-miR-324-5p*** | 978.2 | 347.0 | ***bta-miR-324-5p*** | 1397.8 |
| *mmu-miR-325-3p* | 0.4 | 0.0 |  |  |
| *mmu-miR-325-5p* | 0.4 | 0.0 |  |  |
| *mmu-miR-326-3p* | 1.2 | 27.3 | *bta-miR-326-3p* | 9.0 |
| *mmu-miR-328-3p* | 3.9 | 155.7 | *bta-miR-328-3p* | 34.0 |
| *mmu-miR-329-3p* | 4.9 | 21.3 | *bta-miR-329a-3p* | 1.0 |
| *mmu-miR-329-5p* | 14.7 | 0.7 |  |  |
| *mmu-miR-330-3p* | 161.6 | 50.1 | *bta-miR-330-3p* | 28.4 |
| *mmu-miR-330-5p* | 205.6 | 69.3 |  |  |
| *mmu-miR-331-3p* | 4.6 | 31.3 | *bta-miR-331-3p* | 64.6 |
| *mmu-miR-331-5p* | 25.4 | 55.7 | *bta-miR-331-5p* | 567.6 |
| *mmu-miR-335-3p* | 6.2 | 1.3 |  |  |
| *mmu-miR-335-5p* | 941.8 | 265.6 | *bta-miR-335-5p* | 203.8 |
| *mmu-miR-337-3p* | 11.5 | 14.0 |  |  |
| *mmu-miR-337-5p* | 97.0 | 10.9 |  |  |
| ***mmu-miR-338-3p*** | 3563.8 | 3032.0 | ***bta-miR-338-3p*** | 8581.7 |
| *mmu-miR-338-5p* | 33.9 | 11.4 |  |  |
| *mmu-miR-339-3p* | 161.7 | 31.3 | *bta-miR-339a-5p* | 1389.1 |
| *mmu-miR-339-5p* | 184.6 | 992.3 | *bta-miR-339b-5p* | 900.9 |
| *mmu-miR-340-3p* | 827.6 | 1292.7 | *bta-miR-340-3p* | 716.3 |
| *mmu-miR-340-5p* | 3965.0 | 5274.6 |  |  |
| *mmu-miR-341-3p* | 3.7 | 3.3 |  |  |
| ***mmu-miR-342-3p*** | 1088.8 | 2145.0 | ***bta-miR-342-3p*** | 6524.8 |
| *mmu-miR-342-5p* | 10.2 | 4.0 |  |  |
| *mmu-miR-344b-3p* | 0.0 | 4.0 |  |  |
| *mmu-miR-344b-5p* | 0.0 | 17.3 |  |  |
| *mmu-miR-345-3p* | 876.2 | 221.1 | *bta-miR-345-3p* | 577.1 |
| *mmu-miR-345-5p* | 284.2 | 962.9 | *bta-miR-345-5p* | 7.0 |
|  |  |  | *bta-miR-346-5p* | 0.5 |
| *mmu-miR-350-3p* | 48.8 | 1759.6 |  |  |
| *mmu-miR-350-5p* | 72.5 | 61.4 |  |  |
| *mmu-miR-351-3p* | 0.3 | 0.0 |  |  |
| *mmu-miR-351-5p* | 540.2 | 1273.1 |  |  |
| *mmu-miR-361-3p* | 140.8 | 156.3 |  |  |
| ***mmu-miR-361-5p*** | 1335.3 | 477.0 | ***bta-miR-361-5p*** | 6960.9 |
| *mmu-miR-362-3p* | 94.1 | 1443.3 | *bta-miR-362-3p* | 20.0 |
| ***mmu-miR-362-5p*** | 2828.8 | 2782.4 | ***bta-miR-362-5p*** | 7274.7 |
| *mmu-miR-363-3p* | 26.0 | 15.3 | *bta-miR-363-3p* | 739.5 |
| *mmu-miR-363-5p* | 0.4 | 0.0 | *bta-miR-365-3p* | 457.4 |
| *mmu-miR-365-1-5p* | 0.6 | 0.0 |  |  |
| *mmu-miR-365-2-5p* | 2.1 | 0.0 |  |  |
| *mmu-miR-365-3p* | 599.3 | 1136.0 |  |  |
| *mmu-miR-369-3p* | 122.2 | 308.9 | *bta-miR-369-3p* | 1538.2 |
| *mmu-miR-369-5p* | 0.9 | 0.0 | *bta-miR-369-5p* | 19.5 |
| *mmu-miR-370-3p* | 1.9 | 3.4 | *bta-miR-370-3p* | 29.1 |
| *mmu-miR-370-5p* | 0.3 | 0.0 |  |  |
|  |  |  | *bta-miR-374a-5p* | 6591.0 |
| ***mmu-miR-374b-5p*** | 1333.5 | 2100.7 | ***bta-miR-374b-5p*** | 14271.4 |
| *mmu-miR-374c-3p* | 2.2 | 0.0 |  |  |
| *mmu-miR-374c-5p* | 6.1 | 0.0 |  |  |
| ***mmu-miR-375-3p*** | 10747.2 | 3163.9 | ***bta-miR-375-3p*** | 19881.5 |
| *mmu-miR-375-5p* | 0.0 | 13.3 |  |  |
| *mmu-miR-376a-3p* | 55.1 | 1423.2 | *bta-miR-376a-3p* | 40.1 |
| *mmu-miR-376a-5p* | 2.6 | 0.7 |  |  |
| *mmu-miR-376b-3p* | 106.0 | 61.5 | *bta-miR-376b-3p* | 683.7 |
| *mmu-miR-376b-5p* | 56.9 | 58.6 |  |  |
| *mmu-miR-376c-3p* | 90.9 | 52.1 |  |  |
| *mmu-miR-376c-5p* | 2.9 | 0.7 | *bta-miR-376c-5p* | 18.1 |
|  |  |  | *bta-miR-376d-3p* | 130.7 |
|  |  |  | *bta-miR-376e-3p* | 1211.2 |
| *mmu-miR-377-3p* | 7.7 | 8.0 | *bta-miR-377-3p* | 4.0 |
| *mmu-miR-377-5p* | 0.6 | 0.0 |  |  |
| ***mmu-miR-378a-3p*** | 74696.4 | 33005.8 | ***bta-miR-378-3p*** | 5686.9 |
| *mmu-miR-378a-5p* | 2389.1 | 6938.9 |  |  |
| *mmu-miR-378b-3p* | 47.4 | 15.1 | *bta-miR-378b-3p* | 49.5 |
| *mmu-miR-378c-5p* | 28717.7 | 19633.4 | *bta-miR-378c-5p* | 751.0 |
| *mmu-miR-378d-5p* | 2364.6 | 727.3 |  |  |
| *mmu-miR-379-3p* | 23.5 | 20.0 | *bta-miR-379-5p* | 1681.5 |
| *mmu-miR-379-5p* | 140.3 | 14.4 |  |  |
| *mmu-miR-380-3p* | 18.0 | 37.9 | *bta-miR-380-3p* | 1101.9 |
| *mmu-miR-380-5p* | 2.0 | 6.7 | *bta-miR-380-5p* | 0.5 |
| *mmu-miR-381-3p* | 88.9 | 386.9 | *bta-miR-381-3p* | 422.7 |
| *mmu-miR-381-5p* | 3.3 | 3.3 | *bta-miR-382-5p* | 406.6 |
| *mmu-miR-382-3p* | 7.9 | 7.3 |  |  |
| *mmu-miR-382-5p* | 65.3 | 28.9 |  |  |
| *mmu-miR-384-3p* | 6.1 | 61.4 |  |  |
| *mmu-miR-384-5p* | 4.2 | 0.0 |  |  |
| *mmu-miR-409-3p* | 12.2 | 8.3 |  |  |
| *mmu-miR-409-5p* | 8.2 | 1.4 | *bta-miR-409a-5p* | 89.0 |
| *mmu-miR-410-3p* | 12.5 | 8.7 | *bta-miR-410-3p* | 39.0 |
| *mmu-miR-410-5p* | 1.1 | 0.0 |  |  |
| *mmu-miR-411-3p* | 15.7 | 92.2 |  |  |
| ***mmu-miR-411-5p*** | 723.0 | 1913.7 | ***bta-miR-411a-5p*** | 5686.0 |
|  |  |  | *bta-miR-411b-5p* | 3.0 |
|  |  |  | *bta-miR-411c-3p* | 34.6 |
|  |  |  | *bta-miR-411c-5p* | 428.3 |
| *mmu-miR-412-3p* | 0.3 | 0.0 |  |  |
| *mmu-miR-421-3p* | 195.3 | 68.1 | *bta-miR-421-3p* | 1222.6 |
| *mmu-miR-421-5p* | 0.6 | 0.0 |  |  |
| *mmu-miR-423-3p* | 90.4 | 127.6 | *bta-miR-423-3p* | 2112.7 |
| ***mmu-miR-423-5p*** | 657.2 | 297.3 | ***bta-miR-423-5p*** | 15647.1 |
|  |  |  | *bta-miR-424-3p* | 277.8 |
|  |  |  | *bta-miR-424-5p* | 15027.8 |
| *mmu-miR-425-3p* | 37.7 | 142.3 | *bta-miR-425-3p* | 108.3 |
| ***mmu-miR-425-5p*** | 1061.5 | 1388.9 | ***bta-miR-425-5p*** | 3212.9 |
| ***mmu-miR-429-3p*** | 34443.6 | 21175.3 | ***bta-miR-429-3p*** | 8697.5 |
| *mmu-miR-429-5p* | 0.4 | 0.0 |  |  |
| *mmu-miR-431-3p* | 2.4 | 0.0 |  |  |
| *mmu-miR-431-5p* | 52.2 | 44.4 | *bta-miR-431-5p* | 1.5 |
|  |  |  | *bta-miR-432-5p* | 318.9 |
| *mmu-miR-433-3p* | 9.2 | 2.0 | *bta-miR-433-3p* | 60.1 |
| *mmu-miR-433-5p* | 1.4 | 0.0 |  |  |
| *mmu-miR-434-3p* | 11.5 | 67.9 |  |  |
| *mmu-miR-434-5p* | 15.8 | 47.3 |  |  |
| *mmu-miR-449a-3p* | 0.7 | 0.0 |  |  |
| *mmu-miR-449a-5p* | 21.2 | 0.0 | *bta-miR-449a-5p* | 18.0 |
| *mmu-miR-450a-1-3p* | 42.6 | 8.0 |  |  |
| *mmu-miR-450a-2-3p* | 80.5 | 20.6 |  |  |
| ***mmu-miR-450a-5p*** | 3824.9 | 3906.1 | ***bta-miR-450a-5p*** | 5040.4 |
| *mmu-miR-450b-3p* | 448.7 | 126.0 |  |  |
| *mmu-miR-450b-5p* | 28.0 | 9.3 | *bta-miR-450b-5p* | 342.4 |
| ***mmu-miR-451a-5p*** | 50329.3 | 113641.6 | ***bta-miR-451-5p*** | 11321.8 |
| *mmu-miR-452-3p* | 0.4 | 0.0 |  |  |
| *mmu-miR-452-5p* | 1.1 | 1.6 | *bta-miR-452-5p* | 383.4 |
|  |  |  | *bta-miR-454-3p* | 359.0 |
| *mmu-miR-455-3p* | 219.6 | 986.0 | *bta-miR-455-3p* | 1617.1 |
| *mmu-miR-455-5p* | 212.7 | 255.8 | *bta-miR-455-5p* | 297.3 |
| *mmu-miR-465c-5p* | 0.4 | 0.0 |  |  |
| *mmu-miR-466a-3p* | 2.5 | 26.6 |  |  |
| *mmu-miR-466a-5p* | 21.1 | 44.6 |  |  |
| *mmu-miR-466b-3p* | 2.5 | 26.6 |  |  |
| *mmu-miR-466b-5p* | 26.3 | 7.3 |  |  |
| *mmu-miR-466c-3p* | 2.5 | 26.6 |  |  |
| *mmu-miR-466c-5p* | 51.2 | 6.7 |  |  |
| *mmu-miR-466d-3p* | 4.6 | 35.3 |  |  |
| *mmu-miR-466d-5p* | 21.0 | 4.7 |  |  |
| *mmu-miR-466e-3p* | 2.5 | 26.6 |  |  |
| *mmu-miR-466e-5p* | 17.9 | 6.7 |  |  |
| *mmu-miR-466f-3p* | 0.7 | 0.0 |  |  |
| *mmu-miR-466f-5p* | 42.8 | 90.1 |  |  |
| *mmu-miR-466g-3p* | 1.7 | 0.0 |  |  |
| *mmu-miR-466h-3p* | 0.0 | 3.3 |  |  |
| *mmu-miR-466h-5p* | 100.6 | 2.7 |  |  |
| *mmu-miR-466i-3p* | 0.4 | 0.0 |  |  |
| *mmu-miR-466i-5p* | 12.4 | 3.3 |  |  |
| *mmu-miR-466j-3p* | 18.9 | 4.0 |  |  |
| *mmu-miR-466k-5p* | 3.3 | 2.0 |  |  |
| *mmu-miR-466m-5p* | 39.5 | 10.6 |  |  |
| *mmu-miR-466n-3p* | 8.4 | 9.9 |  |  |
| *mmu-miR-466n-5p* | 21.0 | 4.7 |  |  |
| *mmu-miR-466o-5p* | 26.3 | 7.3 |  |  |
| *mmu-miR-466p-3p* | 2.5 | 26.6 |  |  |
| *mmu-miR-466p-5p* | 28.7 | 45.3 |  |  |
| *mmu-miR-467a-3p* | 1.4 | 3.3 |  |  |
| *mmu-miR-467a-5p* | 460.1 | 278.7 |  |  |
| *mmu-miR-467b-5p* | 460.1 | 278.7 |  |  |
| *mmu-miR-467c-5p* | 252.3 | 98.6 |  |  |
| *mmu-miR-467d-3p* | 1.4 | 3.3 |  |  |
| *mmu-miR-467d-5p* | 294.0 | 176.9 |  |  |
| *mmu-miR-467e-3p* | 0.0 | 5.3 |  |  |
| *mmu-miR-467e-5p* | 443.2 | 206.5 |  |  |
| *mmu-miR-467g-3p* | 0.0 | 2.7 |  |  |
| *mmu-miR-467h-3p* | 1.5 | 0.0 |  |  |
| *mmu-miR-471-5p* | 0.4 | 0.0 |  |  |
|  |  |  | *bta-miR-483-3p* | 23.5 |
| *mmu-miR-484-5p* | 946.2 | 6883.6 | *bta-miR-484-5p* | 547.4 |
| *mmu-miR-485-3p* | 0.4 | 0.0 |  |  |
| *mmu-miR-485-5p* | 2.6 | 0.7 | *bta-miR-485-5p* | 17.1 |
| *mmu-miR-486-3p* | 10.6 | 0.0 |  |  |
| ***mmu-miR-486-5p*** | 1743.2 | 4449.9 | ***bta-miR-486-5p*** | 2415.4 |
|  |  |  | *bta-miR-487a-3p* | 149.7 |
| *mmu-miR-487b-3p* | 14.9 | 5.0 | *bta-miR-487b-3p* | 652.2 |
| *mmu-miR-488-3p* | 1.6 | 0.0 |  |  |
| *mmu-miR-490-3p* | 0.9 | 0.0 |  |  |
| *mmu-miR-491-3p* | 0.6 | 2.0 |  |  |
| *mmu-miR-491-5p* | 10.5 | 0.7 | *bta-miR-491-5p* | 140.0 |
| *mmu-miR-493-3p* | 0.6 | 1.3 | *bta-miR-493-3p* | 79.6 |
| *mmu-miR-493-5p* | 4.6 | 2.9 |  |  |
| *mmu-miR-494-3p* | 26.1 | 7.5 | *bta-miR-494-3p* | 1183.9 |
| *mmu-miR-495-3p* | 7.7 | 2.0 | *bta-miR-495-3p* | 119.5 |
| *mmu-miR-495-5p* | 0.7 | 0.0 |  |  |
| *mmu-miR-496a-3p* | 2.2 | 7.3 |  |  |
| *mmu-miR-497-3p* | 49.3 | 346.6 |  |  |
| ***mmu-miR-497-5p*** | 22956.3 | 13415.2 | ***bta-miR-497-5p*** | 53234.8 |
| *mmu-miR-497b-5p* | 0.4 | 0.0 |  |  |
| *mmu-miR-499-3p* | 0.0 | 6.0 |  |  |
| *mmu-miR-499-5p* | 56.8 | 40.8 | *bta-miR-499-5p* | 1046.9 |
| *mmu-miR-500-3p* | 1899.6 | 4961.8 |  |  |
| *mmu-miR-500-5p* | 28.7 | 3.5 | *bta-miR-500-5p* | 4620.9 |
| *mmu-miR-501-3p* | 516.6 | 758.8 |  |  |
| *mmu-miR-501-5p* | 204.3 | 116.7 |  |  |
|  |  |  | *bta-miR-502a-3p* | 254.2 |
|  |  |  | *bta-miR-502b-3p* | 8.5 |
| *mmu-miR-503-3p* | 7.0 | 6.0 | *bta-miR-503-3p* | 5.5 |
| *mmu-miR-503-5p* | 622.3 | 342.7 | *bta-miR-503-5p* | 191.9 |
|  |  |  | *bta-miR-504-5p* | 0.5 |
| *mmu-miR-505-3p* | 3.2 | 32.6 | *bta-miR-505-3p* | 64.4 |
| *mmu-miR-511-3p* | 194.5 | 21.4 |  |  |
| *mmu-miR-511-5p* | 1.1 | 0.0 |  |  |
| *mmu-miR-532-3p* | 85.7 | 1437.5 |  |  |
| ***mmu-miR-532-5p*** | 2561.2 | 2263.6 | ***bta-miR-532-5p*** | 5479.4 |
| *mmu-miR-539-3p* | 0.0 | 18.6 |  |  |
| *mmu-miR-539-5p* | 1.7 | 0.0 | *bta-miR-539-5p* | 1.5 |
| *mmu-miR-540-3p* | 3.6 | 0.0 |  |  |
| *mmu-miR-540-5p* | 0.0 | 2.0 |  |  |
| *mmu-miR-541-3p* | 0.3 | 0.7 |  |  |
| *mmu-miR-541-5p* | 186.7 | 8.7 |  |  |
| *mmu-miR-542-3p* | 323.7 | 88.2 |  |  |
| *mmu-miR-542-5p* | 51.9 | 2.0 | *bta-miR-542-5p* | 4.5 |
| *mmu-miR-543-3p* | 20.8 | 46.0 | *bta-miR-543-3p* | 370.0 |
| *mmu-miR-543-5p* | 1.6 | 0.0 |  |  |
| *mmu-miR-544-3p* | 0.0 | 3.3 |  |  |
| *mmu-miR-544-5p* | 0.3 | 2.0 |  |  |
|  |  |  | *bta-miR-545-3p* | 14.0 |
|  |  |  | *bta-miR-545-5p* | 116.5 |
| *mmu-miR-547-3p* | 1.7 | 0.0 |  |  |
| *mmu-miR-574-3p* | 30.3 | 670.7 | *bta-miR-574-3p* | 644.5 |
| *mmu-miR-574-5p* | 239.1 | 62.5 |  |  |
| *mmu-miR-582-3p* | 9.3 | 96.1 |  |  |
| *mmu-miR-582-5p* | 225.5 | 1281.0 | *bta-miR-582-5p* | 25.4 |
| *mmu-miR-592-3p* | 0.4 | 0.0 |  |  |
| *mmu-miR-592-5p* | 9.6 | 0.0 | *bta-miR-592-5p* | 53.5 |
| *mmu-miR-598-3p* | 9.1 | 130.4 |  |  |
| *mmu-miR-615-3p* | 86.8 | 448.9 |  |  |
|  |  |  | *bta-miR-628-5p* | 71.9 |
| ***mmu-miR-652-3p*** | 2796.0 | 1021.2 | ***bta-miR-652-3p*** | 18745.8 |
| *mmu-miR-652-5p* | 17.7 | 10.6 |  |  |
|  |  |  | *bta-miR-654-3p* | 0.5 |
|  |  |  | *bta-miR-655-3p* | 11.5 |
|  |  |  | *bta-miR-656-3p* | 0.5 |
|  |  |  | *bta-miR-660-5p* ***^b^*** | 6890.1 |
| *mmu-miR-664-3p* | 22.5 | 83.2 | *bta-miR-664b-3p* | 499.9 |
| *mmu-miR-664-5p* | 36.1 | 113.4 | *bta-miR-664-5p* | 1.0 |
| *mmu-miR-665-3p* | 14.8 | 43.1 | *bta-miR-665-3p* | 178.4 |
| *mmu-miR-666-3p* | 1.1 | 0.0 |  |  |
| *mmu-miR-666-5p* | 0.9 | 0.0 |  |  |
| *mmu-miR-667-3p* | 0.0 | 0.7 |  |  |
| *mmu-miR-667-5p* | 0.7 | 0.0 |  |  |
| *mmu-miR-668-3p* | 5.9 | 9.3 |  |  |
| *mmu-miR-669a-3p* | 5.0 | 68.6 |  |  |
| *mmu-miR-669a-5p* | 77.8 | 0.7 |  |  |
| *mmu-miR-669b-3p* | 0.3 | 0.0 |  |  |
| *mmu-miR-669b-5p* | 77.8 | 2.7 |  |  |
| *mmu-miR-669c-5p* | 66.2 | 8.8 |  |  |
| *mmu-miR-669d-5p* | 338.0 | 574.1 |  |  |
| *mmu-miR-669e-5p* | 9.1 | 0.0 |  |  |
| *mmu-miR-669f-5p* | 45.6 | 10.6 |  |  |
| *mmu-miR-669h-5p* | 1.8 | 0.0 |  |  |
| *mmu-miR-669k-5p* | 0.4 | 0.0 |  |  |
| *mmu-miR-669l-5p* | 12.2 | 0.0 |  |  |
| *mmu-miR-669m-5p* | 39.2 | 8.7 |  |  |
| *mmu-miR-669o-3p* | 5.0 | 68.6 |  |  |
| *mmu-miR-669o-5p* | 50.3 | 39.6 |  |  |
| *mmu-miR-669p-5p* | 77.8 | 0.7 |  |  |
| *mmu-miR-671-3p* | 15.8 | 29.1 |  |  |
|  |  |  | *bta-miR-671-5p* | 251.5 |
| *mmu-miR-671-5p* | 192.0 | 16.0 |  |  |
| *mmu-miR-672-3p* | 0.3 | 0.7 |  |  |
| *mmu-miR-672-5p* | 99.7 | 50.6 |  |  |
| *mmu-miR-673-5p* | 6.3 | 3.3 |  |  |
| *mmu-miR-674-3p* | 42.8 | 151.7 |  |  |
| *mmu-miR-674-5p* ***^m^*** | 1250.0 | 60.1 |  |  |
| *mmu-miR-676-3p* ***^m^*** | 859.2 | 343.8 |  |  |
| *mmu-miR-676-5p* | 80.4 | 417.3 |  |  |
| *mmu-miR-677-3p* | 0.3 | 0.0 |  |  |
| *mmu-miR-677-5p* | 8.2 | 16.0 | *bta-miR-677-5p* | 4.0 |
| *mmu-miR-679-5p* | 1.4 | 0.0 |  |  |
| *mmu-miR-682-3p* | 0.0 | 6.7 |  |  |
| *mmu-miR-684-3p* | 0.0 | 3.3 |  |  |
| *mmu-miR-690-3p* | 5.5 | 447.6 |  |  |
| *mmu-miR-692-3p* | 0.0 | 65.4 |  |  |
| *mmu-miR-693-3p* | 0.3 | 0.0 |  |  |
| *mmu-miR-693-5p* | 0.6 | 0.0 |  |  |
| *mmu-miR-700-3p* | 33.6 | 89.2 |  |  |
| *mmu-miR-700-5p* | 6.1 | 4.7 |  |  |
| *mmu-miR-701-3p* | 1.1 | 0.0 |  |  |
| *mmu-miR-701-5p* | 14.0 | 91.1 |  |  |
| *mmu-miR-702-3p* | 0.0 | 5.3 |  |  |
| *mmu-miR-708-3p* | 95.2 | 401.9 |  |  |
| *mmu-miR-708-5p* | 1784.3 | 826.8 | *bta-miR-708-5p* | 511.9 |
| *mmu-miR-712-3p* | 0.4 | 0.0 |  |  |
| *mmu-miR-712-5p* | 0.0 | 6.0 |  |  |
| *mmu-miR-741-3p* | 0.4 | 0.0 |  |  |
| *mmu-miR-743a-3p* | 0.3 | 0.0 |  |  |
| *mmu-miR-743a-5p* | 0.7 | 0.0 |  |  |
| *mmu-miR-743b-3p* | 0.4 | 0.0 |  |  |
| *mmu-miR-744-3p* | 1.0 | 1.3 |  |  |
| *mmu-miR-744-5p* | 400.2 | 157.3 | *bta-miR-744-5p* | 326.6 |
|  |  |  | *bta-miR-760-3p* | 5.0 |
|  |  |  | *bta-miR-760-5p* | 1.0 |
|  |  |  | *bta-miR-767-5p* | 1.0 |
|  |  |  | *bta-miR-769-5p* | 124.5 |
| *mmu-miR-770-5p* | 1.2 | 0.0 |  |  |
| *mmu-miR-802-3p* | 3.0 | 0.0 |  |  |
| *mmu-miR-802-5p* | 196.5 | 614.6 |  |  |
| *mmu-miR-871-5p* | 0.4 | 0.0 |  |  |
| *mmu-miR-872-3p* | 37.5 | 37.3 |  |  |
| *mmu-miR-872-5p* | 473.8 | 195.2 |  |  |
| *mmu-miR-873a-5p* | 0.3 | 0.0 |  |  |
| *mmu-miR-874-3p* | 28.5 | 101.1 | *bta-miR-874-3p* | 280.6 |
| *mmu-miR-874-5p* | 0.6 | 2.8 |  |  |
| *mmu-miR-875-5p* | 0.0 | 2.7 |  |  |
| *mmu-miR-877-3p* | 0.3 | 0.7 |  |  |
| *mmu-miR-877-5p* | 6.1 | 2.0 | *bta-miR-877-5p* | 60.6 |
|  |  |  | *bta-miR-885-5p* | 41.0 |
|  |  |  | *bta-miR-1185-5p* | 22.0 |
| *mmu-miR-1187-3p* | 0.3 | 0.0 |  |  |
| *mmu-miR-1190-3p* | 0.9 | 2.7 |  |  |
| *mmu-miR-1191-3p* | 138.6 | 286.2 |  |  |
| *mmu-miR-1191b-5p* | 1.4 | 0.0 |  |  |
| *mmu-miR-1193-3p* | 3.9 | 0.7 |  |  |
| *mmu-miR-1194-3p* | 5.2 | 0.0 |  |  |
| *mmu-miR-1195-5p* | 1.1 | 47.9 |  |  |
| *mmu-miR-1198-3p* | 1.0 | 22.7 |  |  |
| *mmu-miR-1198-5p* | 249.1 | 94.9 |  |  |
| *mmu-miR-1199-5p* | 0.0 | 3.3 |  |  |
|  |  |  | *bta-miR-1224-5p* | 1.5 |
|  |  |  | *bta-miR-1246-5p* | 26.0 |
| *mmu-miR-1247-5p* | 0.0 | 12.0 | *bta-miR-1247-5p* | 36.4 |
|  |  |  | *bta-miR-1248-5p* | 7.5 |
| *mmu-miR-1249-3p* | 0.3 | 37.9 | *bta-miR-1249-3p* | 8.5 |
| *mmu-miR-1258-3p* | 10.3 | 0.0 |  |  |
| *mmu-miR-1258-5p* | 7.0 | 10.0 |  |  |
|  |  |  | *bta-miR-1260b-5p* | 38.9 |
|  |  |  | *bta-miR-1271-5p* | 145.0 |
|  |  |  | *bta-miR-1277-3p* | 97.1 |
| *mmu-miR-1291-5p* | 0.3 | 0.0 |  |  |
|  |  |  | *bta-miR-1296-5p* | 21.4 |
| *mmu-miR-1298-5p* | 1.1 | 0.0 | *bta-miR-1298-5p* | 82.5 |
|  |  |  | *bta-miR-1301-3p* | 5.5 |
| *mmu-miR-1306-3p* | 5.8 | 0.0 |  |  |
| *mmu-miR-1306-5p* | 3.2 | 161.0 | *bta-miR-1306-5p* | 355.9 |
|  |  |  | *bta-miR-1307-3p* | 166.6 |
|  |  |  | *bta-miR-1343-3p* | 447.9 |
|  |  |  | *bta-miR-1343-5p* | 0.5 |
|  |  |  | *bta-miR-1388-3p* ***^b^*** | 1216.3 |
|  |  |  | *bta-miR-1388-5p* | 24.5 |
|  |  |  | *bta-miR-1468-5p* ***^b^*** | 17162.0 |
|  |  |  | *bta-miR-1777a-5p* | 2.0 |
|  |  |  | *bta-miR-1814c-5p* | 2.5 |
| *mmu-miR-1839-3p* | 13.5 | 372.2 |  |  |
| ***mmu-miR-1839-5p*** | 3056.8 | 334.8 | ***bta-miR-1839-5p*** | 3577.5 |
| *mmu-miR-1843a-3p* | 43.4 | 203.3 |  |  |
| *mmu-miR-1843a-5p* | 809.6 | 433.4 |  |  |
| *mmu-miR-1843b-3p* | 9.6 | 178.8 |  |  |
| *mmu-miR-1843b-5p* | 1130.4 | 501.2 |  |  |
| *mmu-miR-1927-3p* | 0.4 | 0.0 |  |  |
| *mmu-miR-1929-5p* | 1.3 | 0.0 |  |  |
| *mmu-miR-1930-5p* | 0.0 | 80.4 |  |  |
| *mmu-miR-1931-3p* | 0.3 | 0.0 |  |  |
| *mmu-miR-1932-3p* | 0.7 | 0.0 |  |  |
| *mmu-miR-1933-3p* | 2.7 | 0.0 |  |  |
| *mmu-miR-1933-5p* | 0.6 | 0.0 |  |  |
| *mmu-miR-1934-5p* | 3.5 | 6.0 |  |  |
|  |  |  | *bta-miR-1940-5p* | 0.5 |
| *mmu-miR-1941-3p* | 5.4 | 0.0 |  |  |
| *mmu-miR-1941-5p* | 1.7 | 0.0 |  |  |
| *mmu-miR-1943-3p* | 0.7 | 9.3 |  |  |
| *mmu-miR-1943-5p* | 1.5 | 0.0 |  |  |
| *mmu-miR-1945-3p* | 3.6 | 0.0 |  |  |
| *mmu-miR-1946a-5p* | 0.0 | 1.3 |  |  |
| *mmu-miR-1948-3p* | 1.4 | 0.0 |  |  |
| *mmu-miR-1948-5p* | 1.4 | 0.0 |  |  |
| *mmu-miR-1949-3p* | 9.0 | 3.4 |  |  |
| *mmu-miR-1950-5p* | 0.9 | 0.0 |  |  |
| *mmu-miR-1954-5p* | 1.7 | 2.0 |  |  |
| *mmu-miR-1955-5p* | 18.2 | 63.7 |  |  |
| *mmu-miR-1956-5p* | 1.5 | 0.0 |  |  |
| *mmu-miR-1957a-5p* | 18.1 | 12.6 |  |  |
| *mmu-miR-1958-3p* | 0.6 | 0.0 |  |  |
| *mmu-miR-1960-5p* | 1.2 | 0.0 |  |  |
| *mmu-miR-1964-3p* | 8.4 | 24.6 |  |  |
| *mmu-miR-1964-5p* | 2.1 | 0.0 |  |  |
| *mmu-miR-1968-3p* | 0.3 | 0.0 |  |  |
| *mmu-miR-1968-5p* | 11.5 | 2.0 |  |  |
| *mmu-miR-1981-3p* | 7.4 | 24.6 |  |  |
| *mmu-miR-1981-5p* | 111.6 | 17.3 |  |  |
| *mmu-miR-1982-3p* | 0.0 | 1.3 |  |  |
| *mmu-miR-1983-3p* | 272.8 | 445.5 |  |  |
|  |  |  | *bta-miR-2284a-5p* | 3.5 |
|  |  |  | *bta-miR-2284aa-5p* | 630.7 |
|  |  |  | *bta-miR-2284ab-5p* ***^b^*** | 1225.7 |
|  |  |  | *bta-miR-2284ac-5p* | 12.5 |
|  |  |  | *bta-miR-2284b-5p* | 19.0 |
|  |  |  | *bta-miR-2284c-5p* | 7.0 |
|  |  |  | *bta-miR-2284d-5p* | 11.0 |
|  |  |  | *bta-miR-2284f-5p* | 0.5 |
|  |  |  | *bta-miR-2284g-5p* | 0.5 |
|  |  |  | *bta-miR-2284h-5p* | 3.0 |
|  |  |  | *bta-miR-2284j-5p* | 44.4 |
|  |  |  | *bta-miR-2284k-5p* | 3.0 |
|  |  |  | *bta-miR-2284l-5p* | 10.5 |
|  |  |  | *bta-miR-2284m-5p* | 11.5 |
|  |  |  | *bta-miR-2284n-5p* | 10.5 |
|  |  |  | *bta-miR-2284o-5p* | 15.0 |
|  |  |  | *bta-miR-2284p-5p* | 5.5 |
|  |  |  | *bta-miR-2284t-3p* | 4.5 |
|  |  |  | *bta-miR-2284u-3p* | 1.0 |
|  |  |  | *bta-miR-2284v-3p* | 70.2 |
|  |  |  | *bta-miR-2284w-5p* | 171.4 |
|  |  |  | *bta-miR-2284x-5p* ***^b^*** | 19662.2 |
|  |  |  | *bta-miR-2284y-5p* | 507.5 |
|  |  |  | *bta-miR-2284z-5p* ***^b^*** | 1966.8 |
|  |  |  | *bta-miR-2285aa-3p* | 85.5 |
|  |  |  | *bta-miR-2285ab-3p* | 0.5 |
|  |  |  | *bta-miR-2285ac-3p* | 3.0 |
|  |  |  | *bta-miR-2285ad-3p* | 2.5 |
|  |  |  | *bta-miR-2285af-5p* | 5.5 |
|  |  |  | *bta-miR-2285b-3p* | 20.0 |
|  |  |  | *bta-miR-2285c-3p* | 0.5 |
|  |  |  | *bta-miR-2285e-3p* | 9.5 |
|  |  |  | *bta-miR-2285f-3p* | 14.9 |
|  |  |  | *bta-miR-2285g-3p* | 3.0 |
|  |  |  | *bta-miR-2285h-3p* | 1.5 |
|  |  |  | *bta-miR-2285i-3p* | 1.5 |
|  |  |  | *bta-miR-2285j-3p* | 2.5 |
|  |  |  | *bta-miR-2285k-3p* | 61.0 |
|  |  |  | *bta-miR-2285l-3p* | 2.5 |
|  |  |  | *bta-miR-2285n-3p* | 2.5 |
|  |  |  | *bta-miR-2285o-3p* | 37.0 |
|  |  |  | *bta-miR-2285p-3p* | 29.0 |
|  |  |  | *bta-miR-2285q-3p* | 3.5 |
|  |  |  | *bta-miR-2285r-3p* | 0.5 |
|  |  |  | *bta-miR-2285t-3p* | 300.4 |
|  |  |  | *bta-miR-2285u-3p* | 5.5 |
|  |  |  | *bta-miR-2290-3p* | 0.5 |
|  |  |  | *bta-miR-2293-5p* | 0.5 |
|  |  |  | *bta-miR-2295-3p* | 1.0 |
|  |  |  | *bta-miR-2299-3p* | 181.0 |
|  |  |  | *bta-miR-2299-5p* | 1.0 |
|  |  |  | *bta-miR-2300a-5p* | 0.5 |
|  |  |  | *bta-miR-2300b-3p* | 1.5 |
|  |  |  | *bta-miR-2308-3p* | 8.0 |
|  |  |  | *bta-miR-2310-5p* | 2.5 |
|  |  |  | *bta-miR-2311-5p* | 8.5 |
|  |  |  | *bta-miR-2312-3p* | 1.0 |
|  |  |  | *bta-miR-2313-3p* | 1.0 |
|  |  |  | *bta-miR-2313-5p* | 3.5 |
|  |  |  | *bta-miR-2314-5p* | 0.5 |
|  |  |  | *bta-miR-2316-5p* | 3.5 |
|  |  |  | *bta-miR-2320-3p* | 10.0 |
|  |  |  | *bta-miR-2320-5p* | 9.0 |
|  |  |  | *bta-miR-2323-5p* | 0.5 |
|  |  |  | *bta-miR-2328-5p* | 0.5 |
|  |  |  | *bta-miR-2331-3p* | 7.0 |
|  |  |  | *bta-miR-2332-3p* | 9.0 |
|  |  |  | *bta-miR-2336-3p* | 70.1 |
|  |  |  | *bta-miR-2339-3p* | 3.0 |
|  |  |  | *bta-miR-2340-3p* | 12.0 |
|  |  |  | *bta-miR-2342-3p* | 0.5 |
|  |  |  | *bta-miR-2344-3p* | 1.0 |
|  |  |  | *bta-miR-2346-5p* | 17.5 |
|  |  |  | *bta-miR-2350-5p* | 2.5 |
|  |  |  | *bta-miR-2354-5p* | 1.0 |
|  |  |  | *bta-miR-2355-3p* | 5.5 |
|  |  |  | *bta-miR-2366-3p* | 0.5 |
|  |  |  | *bta-miR-2367-3p* | 0.5 |
|  |  |  | *bta-miR-2369-3p* | 0.5 |
|  |  |  | *bta-miR-2370-5p* | 1.0 |
|  |  |  | *bta-miR-2376-3p* | 6.5 |
|  |  |  | *bta-miR-2382-5p* | 1.0 |
|  |  |  | *bta-miR-2384-5p* | 1.5 |
|  |  |  | *bta-miR-2387-5p* | 53.9 |
|  |  |  | *bta-miR-2396-3p* | 3.5 |
|  |  |  | *bta-miR-2397-3p* | 10.5 |
|  |  |  | *bta-miR-2397-5p* | 8.9 |
|  |  |  | *bta-miR-2399-3p* | 24.5 |
|  |  |  | *bta-miR-2399-5p* | 10.5 |
|  |  |  | *bta-miR-2403-3p* | 2.0 |
|  |  |  | *bta-miR-2404-5p* | 1.0 |
|  |  |  | *bta-miR-2407-3p* | 0.5 |
|  |  |  | *bta-miR-2408-3p* | 3.0 |
|  |  |  | *bta-miR-2409-3p* | 1.0 |
|  |  |  | *bta-miR-2410-5p* | 1.0 |
|  |  |  | *bta-miR-2411-3p* | 4.0 |
|  |  |  | *bta-miR-2411-5p* | 43.5 |
|  |  |  | *bta-miR-2416-3p* | 1.0 |
|  |  |  | *bta-miR-2419-3p* | 83.0 |
|  |  |  | *bta-miR-2419-5p* | 639.7 |
|  |  |  | *bta-miR-2422-5p* | 3.5 |
|  |  |  | *bta-miR-2424-3p* | 1.0 |
|  |  |  | *bta-miR-2425-3p* | 0.5 |
|  |  |  | *bta-miR-2431-5p* | 19.5 |
|  |  |  | *bta-miR-2432-3p* | 0.5 |
|  |  |  | *bta-miR-2436-3p* | 1.0 |
|  |  |  | *bta-miR-2441-3p* | 0.5 |
|  |  |  | *bta-miR-2443-5p* | 68.9 |
|  |  |  | *bta-miR-2447-5p* | 13.0 |
|  |  |  | *bta-miR-2448-3p* | 35.6 |
|  |  |  | *bta-miR-2448-5p* | 3.5 |
|  |  |  | *bta-miR-2450c-3p* | 1.0 |
|  |  |  | *bta-miR-2451-3p* | 0.5 |
|  |  |  | *bta-miR-2453-5p* | 5.5 |
|  |  |  | *bta-miR-2457-3p* | 4.0 |
|  |  |  | *bta-miR-2460-5p* | 0.5 |
|  |  |  | *bta-miR-2463-5p* | 10.0 |
|  |  |  | *bta-miR-2465-5p* | 2.5 |
|  |  |  | *bta-miR-2468-5p* | 12.0 |
|  |  |  | *bta-miR-2469-5p* | 0.5 |
|  |  |  | *bta-miR-2473-3p* | 0.5 |
|  |  |  | *bta-miR-2474-3p* | 7.0 |
|  |  |  | *bta-miR-2475-3p* | 0.5 |
|  |  |  | *bta-miR-2478-3p* | 17.9 |
|  |  |  | *bta-miR-2481-5p* | 2.0 |
|  |  |  | *bta-miR-2483-3p* | 14.0 |
|  |  |  | *bta-miR-2483-5p* | 6.0 |
|  |  |  | *bta-miR-2484-3p* | 0.5 |
|  |  |  | *bta-miR-2487-3p* | 16.5 |
|  |  |  | *bta-miR-2887-5p* | 11.0 |
|  |  |  | *bta-miR-2904-5p* | 26.0 |
| *mmu-miR-3057-5p* | 6.1 | 0.7 |  |  |
| *mmu-miR-3058-3p* | 15.7 | 8.1 |  |  |
| *mmu-miR-3058-5p* | 17.1 | 49.2 |  |  |
| *mmu-miR-3060-3p* | 2.1 | 4.7 |  |  |
| *mmu-miR-3061-3p* | 0.7 | 2.7 |  |  |
| *mmu-miR-3061-5p* | 5.3 | 0.0 |  |  |
| *mmu-miR-3064-5p* | 12.3 | 2.0 |  |  |
| *mmu-miR-3065-5p* | 1.7 | 2.0 |  |  |
| *mmu-miR-3066-3p* | 0.0 | 4.0 |  |  |
| *mmu-miR-3066-5p* | 5.8 | 0.0 |  |  |
| *mmu-miR-3068-3p* | 39.3 | 55.9 |  |  |
| *mmu-miR-3068-5p* ***^m^*** | 3914.7 | 976.1 |  |  |
| *mmu-miR-3069-5p* | 6.8 | 0.0 |  |  |
| *mmu-miR-3072-3p* | 1.9 | 2.7 |  |  |
| *mmu-miR-3073a-3p* | 2.0 | 0.0 |  |  |
| *mmu-miR-3073b-5p* | 2.4 | 0.0 |  |  |
| *mmu-miR-3074-1-3p* | 3.0 | 0.0 |  |  |
| *mmu-miR-3074-2-3p* | 0.4 | 0.0 |  |  |
| *mmu-miR-3074-5p* | 2.8 | 0.7 |  |  |
| *mmu-miR-3076-5p* | 8.4 | 0.0 |  |  |
| *mmu-miR-3079-3p* | 0.6 | 0.0 |  |  |
| *mmu-miR-3079-5p* | 0.6 | 0.0 |  |  |
| *mmu-miR-3081-3p* | 7.4 | 42.5 |  |  |
| *mmu-miR-3082-3p* | 0.0 | 2.0 |  |  |
| *mmu-miR-3082-5p* | 5.3 | 0.0 |  |  |
| *mmu-miR-3083-5p* | 0.3 | 0.0 |  |  |
| *mmu-miR-3084-3p* | 0.7 | 2.7 |  |  |
| *mmu-miR-3084-5p* | 29.8 | 0.0 |  |  |
| *mmu-miR-3086-5p* | 0.4 | 0.0 |  |  |
| *mmu-miR-3087-3p* | 1.0 | 0.0 |  |  |
| *mmu-miR-3088-3p* | 1.1 | 0.0 |  |  |
| *mmu-miR-3089-3p* | 0.3 | 0.0 |  |  |
| *mmu-miR-3089-5p* | 0.0 | 2.7 |  |  |
| *mmu-miR-3091-5p* | 5.6 | 0.0 |  |  |
| *mmu-miR-3094-3p* | 0.0 | 3.3 |  |  |
| *mmu-miR-3094-5p* | 0.3 | 0.0 |  |  |
| *mmu-miR-3095-3p* | 3.2 | 0.0 |  |  |
| *mmu-miR-3097-5p* | 0.9 | 0.0 |  |  |
| *mmu-miR-3098-3p* | 0.3 | 0.0 |  |  |
| *mmu-miR-3098-5p* | 2.1 | 0.0 |  |  |
| *mmu-miR-3102-3p* | 0.0 | 14.0 |  |  |
| *mmu-miR-3105-3p* | 39.1 | 121.0 |  |  |
| *mmu-miR-3105-5p* ***^m^*** | 814.2 | 291.2 |  |  |
| *mmu-miR-3107-3p* | 0.6 | 0.0 |  |  |
| *mmu-miR-3107-5p* ***^m^*** | 1740.7 | 4455.4 |  |  |
| *mmu-miR-3108-5p* | 2.1 | 2.7 |  |  |
| *mmu-miR-3109-3p* | 1.3 | 6.7 |  |  |
| *mmu-miR-3109-5p* | 1.4 | 0.0 |  |  |
| *mmu-miR-3112-3p* | 0.0 | 3.3 |  |  |
| *mmu-miR-3112-5p* | 0.4 | 0.0 |  |  |
|  |  |  | *bta-miR-3120-3p* | 2.0 |
|  |  |  | *bta-miR-3141-3p* | 1.0 |
|  |  |  | *bta-miR-3431-5p* ***^b^*** | 2196.4 |
|  |  |  | *bta-miR-3432-5p* ***^b^*** | 4377.9 |
| *mmu-miR-3470a-5p* | 6.4 | 509.5 |  |  |
| *mmu-miR-3470b-5p* | 8.8 | 621.4 |  |  |
| *mmu-miR-3471-5p* | 1.4 | 2.7 |  |  |
| *mmu-miR-3473a-3p* | 15.6 | 34.8 |  |  |
| *mmu-miR-3473b-3p* | 6.4 | 117.2 |  |  |
| *mmu-miR-3473d-5p* | 36.3 | 94.5 |  |  |
| *mmu-miR-3473e-5p* | 4.3 | 89.8 |  |  |
| *mmu-miR-3473f-3p* | 0.4 | 0.0 |  |  |
| *mmu-miR-3474-5p* | 1.8 | 0.0 |  |  |
| *mmu-miR-3535-5p* | 6.5 | 9.3 |  |  |
| *mmu-miR-3569-3p* | 0.0 | 0.7 |  |  |
| *mmu-miR-3572-5p* | 1.1 | 0.0 |  |  |
|  |  |  | *bta-miR-3613-5p* | 22.5 |
|  |  |  | *bta-miR-3660-3p* | 4.5 |
|  |  |  | *bta-miR-3956-5p* | 9.5 |
|  |  |  | *bta-miR-3957-5p* | 2.5 |
| *mmu-miR-3962-3p* | 1.3 | 0.0 |  |  |
| *mmu-miR-3963-5p* | 59.4 | 1.3 |  |  |
| *mmu-miR-3966-5p* | 0.6 | 0.0 |  |  |
| *mmu-miR-3967-3p* | 1.5 | 0.0 |  |  |
| *mmu-miR-3968-3p* | 16.7 | 6.0 |  |  |
| *mmu-miR-3970-5p* | 93.8 | 115.1 |  |  |
| *mmu-miR-5046-5p* | 0.0 | 0.8 |  |  |
| *mmu-miR-5098-5p* | 0.0 | 1.3 |  |  |
| *mmu-miR-5099-3p* | 199.7 | 34.8 |  |  |
| *mmu-miR-5100-3p* | 415.0 | 67.8 |  |  |
| *mmu-miR-5101-3p* | 7.8 | 6.0 |  |  |
| *mmu-miR-5103-3p* | 5.4 | 9.3 |  |  |
| *mmu-miR-5104-3p* | 10.8 | 0.7 |  |  |
| *mmu-miR-5106-3p* | 1.2 | 1.3 |  |  |
| *mmu-miR-5112-5p* | 0.6 | 0.0 |  |  |
| *mmu-miR-5113-5p* | 6.8 | 0.0 |  |  |
| *mmu-miR-5114-5p* | 3.3 | 4.7 |  |  |
| *mmu-miR-5116-5p* | 3.0 | 0.0 |  |  |
| *mmu-miR-5119-3p* | 1.6 | 14.4 |  |  |
| *mmu-miR-5121-3p* | 23.0 | 117.5 |  |  |
| *mmu-miR-5122-5p* | 3.3 | 0.0 |  |  |
| *mmu-miR-5123-3p* | 155.7 | 68.7 |  |  |
| *mmu-miR-5124a-5p* | 1.3 | 0.0 |  |  |
| *mmu-miR-5125-3p* | 2.7 | 3.3 |  |  |
| *mmu-miR-5126-3p* | 11.1 | 52.9 |  |  |
| *mmu-miR-5128-3p* | 0.0 | 3.3 |  |  |
| *mmu-miR-5129-5p* | 0.9 | 0.0 |  |  |
| *mmu-miR-5131-3p* | 0.3 | 0.0 |  |  |
| *mmu-miR-5132-5p* | 11.3 | 0.0 |  |  |
| *mmu-miR-5615-3p* | 0.0 | 0.7 |  |  |
| *mmu-miR-5615-5p* | 3.7 | 4.7 |  |  |
| *mmu-miR-5616-3p* | 1.1 | 0.0 |  |  |
| *mmu-miR-5619-3p* | 1.4 | 0.0 |  |  |
| *mmu-miR-5619-5p* | 3.6 | 3.3 |  |  |
| *mmu-miR-5620-5p* | 0.3 | 0.0 |  |  |
| *mmu-miR-5623-5p* | 0.3 | 0.0 |  |  |
| *mmu-miR-5624-3p* | 1.7 | 0.0 |  |  |
| *mmu-miR-5626-3p* | 0.6 | 0.0 |  |  |
| *mmu-miR-5627-5p* | 4.3 | 51.1 |  |  |
| *mmu-miR-5709-3p* | 0.4 | 0.0 |  |  |
| *mmu-miR-5709-5p* | 0.7 | 0.0 |  |  |
|  |  |  | *bta-miR-6119-3p* | 4.5 |
|  |  |  | *bta-miR-6119-5p* | 472.1 |
|  |  |  | *bta-miR-6120-3p* | 363.8 |
|  |  |  | *bta-miR-6120-5p* | 2.5 |
|  |  |  | *bta-miR-6123-5p* | 80.0 |
| *mmu-miR-6236-3p* | 1.7 | 0.0 |  |  |
| *mmu-miR-6239-3p* | 2.2 | 6.7 |  |  |
| *mmu-miR-6240-5p* | 23.6 | 150.1 |  |  |
| *mmu-miR-6380-3p* | 0.0 | 2.7 |  |  |
| *mmu-miR-6392-5p* | 1.3 | 0.0 |  |  |
| *mmu-miR-6481-3p* | 0.0 | 2.0 |  |  |
| *mmu-miR-6516-5p* | 14.8 | 0.0 | *bta-miR-6516-5p* | 135.4 |
|  |  |  | *bta-miR-6517-5p* | 89.5 |
|  |  |  | *bta-miR-6518-3p* | 5.0 |
|  |  |  | *bta-miR-6520-5p* | 25.5 |
|  |  |  | *bta-miR-6522-5p* | 129.9 |
|  |  |  | *bta-miR-6523-5p* | 28.5 |
|  |  |  | *bta-miR-6524-3p* | 784.3 |
|  |  |  | *bta-miR-6525-5p* | 49.9 |
|  |  |  | *bta-miR-6526-3p* | 2.5 |
|  |  |  | *bta-miR-6527-3p* | 1.5 |
|  |  |  | *bta-miR-6528-5p* | 1.0 |
|  |  |  | *bta-miR-6529-5p* | 243.9 |
|  |  |  | *bta-miR-6530-5p* | 0.5 |
|  |  |  | *bta-miR-6531-5p* | 3.0 |
|  |  |  | *bta-miR-6532-3p* | 0.5 |
|  |  |  | *bta-miR-6533-3p* | 2.5 |
|  |  |  | *bta-miR-6534-3p* | 1.0 |
|  |  |  | *bta-miR-6535-5p* | 1.0 |
|  |  |  | *bta-miR-6536-5p* | 0.5 |
| *mmu-miR-6537-3p* | 0.4 | 0.0 |  |  |
| *mmu-miR-6538-5p* | 1.7 | 0.0 |  |  |
| *mmu-miR-6540-5p* | 0.6 | 0.0 |  |  |
| *mmu-miR-6546-3p* | 7.3 | 11.3 |  |  |
| *mmu-miR-6546-5p* | 4.6 | 5.3 |  |  |
| *mmu-miR-6715-3p* | 1.1 | 2.0 |  |  |
| *mmu-miR-6897-5p* | 2.5 | 0.0 |  |  |
| *mmu-miR-6899-3p* | 0.3 | 0.0 |  |  |
| *mmu-miR-6900-5p* | 0.6 | 0.0 |  |  |
| *mmu-miR-6902-3p* | 0.3 | 0.0 |  |  |
| *mmu-miR-6904-5p* | 0.6 | 0.0 |  |  |
| *mmu-miR-6905-5p* | 0.6 | 0.0 |  |  |
| *mmu-miR-6907-3p* | 0.6 | 0.0 |  |  |
| *mmu-miR-6908-3p* | 0.6 | 0.0 |  |  |
| *mmu-miR-6912-5p* | 0.6 | 0.0 |  |  |
| *mmu-miR-6913-3p* | 0.7 | 0.0 |  |  |
| *mmu-miR-6914-5p* | 0.4 | 0.0 |  |  |
| *mmu-miR-6917-5p* | 0.3 | 0.0 |  |  |
| *mmu-miR-6919-5p* | 2.6 | 0.0 |  |  |
| *mmu-miR-6921-3p* | 0.0 | 4.0 |  |  |
| *mmu-miR-6922-5p* | 0.3 | 0.0 |  |  |
| *mmu-miR-6925-5p* | 0.3 | 0.0 |  |  |
| *mmu-miR-6929-3p* | 0.4 | 0.0 |  |  |
| *mmu-miR-6930-5p* | 0.3 | 0.0 |  |  |
| *mmu-miR-6932-3p* | 0.0 | 3.3 |  |  |
| *mmu-miR-6932-5p* | 0.9 | 0.0 |  |  |
| *mmu-miR-6933-5p* | 2.0 | 0.0 |  |  |
| *mmu-miR-6935-5p* | 0.6 | 0.0 |  |  |
| *mmu-miR-6936-3p* | 0.0 | 0.7 |  |  |
| *mmu-miR-6938-5p* | 0.0 | 0.7 |  |  |
| *mmu-miR-6939-3p* | 2.6 | 2.7 |  |  |
| *mmu-miR-6940-3p* | 0.3 | 0.0 |  |  |
| *mmu-miR-6942-5p* | 0.4 | 0.0 |  |  |
| *mmu-miR-6943-5p* | 0.3 | 0.0 |  |  |
| *mmu-miR-6944-3p* | 0.0 | 0.7 |  |  |
| *mmu-miR-6945-3p* | 0.0 | 0.7 |  |  |
| *mmu-miR-6946-3p* | 0.0 | 4.0 |  |  |
| *mmu-miR-6946-5p* | 1.1 | 0.0 |  |  |
| *mmu-miR-6948-3p* | 0.4 | 0.0 |  |  |
| *mmu-miR-6948-5p* | 1.4 | 0.0 |  |  |
| *mmu-miR-6950-5p* | 0.6 | 0.0 |  |  |
| *mmu-miR-6951-5p* | 2.1 | 0.0 |  |  |
| *mmu-miR-6952-5p* | 0.4 | 0.0 |  |  |
| *mmu-miR-6954-5p* | 0.3 | 0.0 |  |  |
| *mmu-miR-6955-5p* | 1.1 | 0.0 |  |  |
| *mmu-miR-6957-5p* | 0.4 | 0.0 |  |  |
| *mmu-miR-696-3p* | 1.1 | 2.0 |  |  |
| *mmu-miR-6960-5p* | 0.3 | 0.0 |  |  |
| *mmu-miR-6966-3p* | 0.4 | 0.0 |  |  |
| *mmu-miR-6967-5p* | 2.1 | 0.0 |  |  |
| *mmu-miR-6976-5p* | 0.3 | 0.0 |  |  |
| *mmu-miR-6979-3p* | 0.0 | 0.8 |  |  |
| *mmu-miR-6980-3p* | 0.0 | 0.7 |  |  |
| *mmu-miR-6981-3p* | 0.3 | 0.0 |  |  |
| *mmu-miR-6985-3p* | 0.3 | 0.0 |  |  |
| *mmu-miR-6990-3p* | 0.0 | 2.7 |  |  |
| *mmu-miR-6990-5p* | 1.7 | 0.0 |  |  |
| *mmu-miR-6992-3p* | 0.6 | 0.0 |  |  |
| *mmu-miR-6992-5p* | 0.0 | 4.0 |  |  |
| *mmu-miR-6993-3p* | 2.8 | 0.0 |  |  |
| *mmu-miR-6993-5p* | 0.6 | 0.0 |  |  |
| *mmu-miR-6994-3p* | 0.0 | 0.7 |  |  |
| *mmu-miR-6994-5p* | 0.6 | 0.0 |  |  |
| *mmu-miR-6996-3p* | 0.9 | 0.0 |  |  |
| *mmu-miR-6996-5p* | 1.4 | 0.0 |  |  |
| *mmu-miR-6998-3p* | 0.3 | 0.0 |  |  |
| *mmu-miR-7007-3p* | 0.4 | 0.0 |  |  |
| *mmu-miR-7009-5p* | 0.4 | 0.0 |  |  |
| *mmu-miR-7010-5p* | 0.3 | 0.0 |  |  |
| *mmu-miR-7013-5p* | 3.4 | 0.0 |  |  |
| *mmu-miR-7015-3p* | 0.0 | 2.0 |  |  |
| *mmu-miR-7021-5p* | 1.8 | 0.0 |  |  |
| *mmu-miR-7024-5p* | 0.9 | 0.0 |  |  |
| *mmu-miR-7026-5p* | 0.4 | 0.0 |  |  |
| *mmu-miR-7029-3p* | 1.0 | 0.0 |  |  |
| *mmu-miR-7030-3p* | 0.0 | 1.3 |  |  |
| *mmu-miR-7031-5p* | 0.9 | 0.0 |  |  |
| *mmu-miR-7032-3p* | 0.9 | 50.9 |  |  |
| *mmu-miR-7032-5p* | 1.5 | 0.0 |  |  |
| *mmu-miR-7033-5p* | 4.2 | 0.0 |  |  |
| *mmu-miR-7036-3p* | 0.0 | 0.7 |  |  |
| *mmu-miR-7037-3p* | 0.0 | 0.7 |  |  |
| *mmu-miR-7037-5p* | 0.3 | 0.0 |  |  |
| *mmu-miR-7041-5p* | 0.3 | 0.0 |  |  |
| *mmu-miR-7042-5p* | 0.7 | 0.0 |  |  |
| *mmu-miR-7043-3p* | 3.2 | 0.0 |  |  |
| *mmu-miR-7043-5p* | 9.3 | 0.0 |  |  |
| *mmu-miR-7049-5p* | 3.8 | 0.0 |  |  |
| *mmu-miR-7051-5p* | 2.5 | 0.0 |  |  |
| *mmu-miR-7057-3p* | 0.3 | 0.0 |  |  |
| *mmu-miR-7059-5p* | 0.9 | 0.0 |  |  |
| *mmu-miR-7063-5p* | 1.0 | 0.7 |  |  |
| *mmu-miR-7064-5p* | 5.9 | 0.0 |  |  |
| *mmu-miR-7065-3p* | 5.1 | 0.0 |  |  |
| *mmu-miR-7071-3p* | 0.6 | 0.7 |  |  |
| *mmu-miR-7083-5p* | 0.9 | 0.0 |  |  |
| *mmu-miR-7084-3p* | 0.0 | 1.3 |  |  |
| *mmu-miR-7084-5p* | 0.6 | 0.0 |  |  |
| *mmu-miR-7085-3p* | 0.0 | 1.3 |  |  |
| *mmu-miR-7085-5p* | 0.6 | 0.0 |  |  |
| *mmu-miR-7092-3p* | 0.4 | 0.0 |  |  |
| *mmu-miR-7093-3p* | 0.0 | 4.0 |  |  |
| *mmu-miR-7116-3p* | 0.3 | 0.0 |  |  |
| *mmu-miR-7212-3p* | 0.3 | 6.7 |  |  |
| *mmu-miR-7212-5p* | 5.7 | 0.0 |  |  |
| *mmu-miR-7219-3p* | 2.7 | 8.7 |  |  |
| *mmu-miR-7224-3p* | 0.3 | 0.0 |  |  |
| *mmu-miR-7235-3p* | 0.6 | 0.0 |  |  |
| *mmu-miR-7236-3p* | 2.0 | 1.3 |  |  |
| *mmu-miR-7237-3p* | 0.3 | 0.0 |  |  |
| *mmu-miR-7647-3p* | 4.8 | 14.7 |  |  |
| *mmu-miR-7647-5p* | 0.0 | 8.0 |  |  |
| *mmu-miR-7649-3p* | 0.7 | 0.0 |  |  |
| *mmu-miR-7651-3p* | 0.0 | 57.4 |  |  |
| *mmu-miR-7652-3p* | 0.3 | 0.0 |  |  |
| *mmu-miR-7655-3p* | 0.3 | 0.0 |  |  |
| *mmu-miR-7655-5p* | 0.3 | 0.0 |  |  |
| *mmu-miR-7662-3p* | 4.7 | 0.0 |  |  |
| *mmu-miR-7664-3p* | 0.4 | 0.0 |  |  |
| *mmu-miR-7667-5p* | 1.1 | 3.3 |  |  |
| *mmu-miR-7669-3p* | 0.4 | 0.0 |  |  |
| *mmu-miR-7669-5p* | 0.3 | 2.7 |  |  |
| *mmu-miR-7670-3p* | 0.4 | 0.0 |  |  |
| *mmu-miR-7672-5p* | 0.3 | 0.0 |  |  |
| *mmu-miR-7673-5p* | 1.9 | 0.0 |  |  |
| *mmu-miR-7676-5p* | 0.9 | 0.0 |  |  |
| *mmu-miR-7680-5p* | 0.4 | 0.0 |  |  |
| *mmu-miR-7687-3p* | 0.0 | 0.7 |  |  |
| *mmu-miR-7689-3p* | 0.7 | 0.0 |  |  |
|  |  |  | *bta-miR-7857-5p* | 22.5 |
|  |  |  | *bta-miR-7858-3p* | 1.0 |
|  |  |  | *bta-miR-7859-3p* | 371.9 |
|  |  |  | *bta-miR-7860-3p* | 10.0 |
|  |  |  | *bta-miR-7861-3p* | 21.5 |
|  |  |  | *bta-miR-7862-3p* | 64.5 |
|  |  |  | *bta-miR-7864-3p* | 3.5 |
| *mmu-miR-8091-5p* | 0.3 | 0.0 |  |  |
| *mmu-miR-8094-5p* | 0.3 | 0.0 |  |  |
| *mmu-miR-8103-3p* | 9.1 | 4.7 |  |  |
| *mmu-miR-8106-3p* | 2.3 | 0.0 |  |  |
| *mmu-miR-8109-5p* | 0.0 | 10.1 |  |  |
| *mmu-miR-8111-3p* | 0.6 | 0.0 |  |  |
| *mmu-miR-8112-3p* | 0.6 | 11.3 |  |  |
| *mmu-miR-8120-5p* | 0.3 | 0.0 |  |  |

In bold: common miRNA to both species with expression above 100 RPM.

**^m^**, **^b^**: miRNA with mouse or bovine-specific expression above 100 RPM, respectively, not described in the other species.

**B.**

| **miRNA** | **Mouse** | **AGO2** | **miRNA** | **Bovine** |
| --- | --- | --- | --- | --- |
| *hsa-miR-374b-3p* | 58.8 | 34.1 | *cgr-miR-191-3p* | 339.9 |
|  |  |  | *eca-miR-450b-3p* | 25.4 |
|  |  |  | *eca-miR-615-3p* | 124.4 |
|  |  |  | *gga-miR-18b-3p* | 1.0 |
|  |  |  | *gga-miR-29b-2-5p* | 1443.1 |
|  |  |  | *hsa-let-7c-3p* | 7.0 |
|  |  |  | *hsa-let-7d-3p* | 991.4 |
|  |  |  | *hsa-let-7e-3p* | 30.0 |
|  |  |  | *hsa-let-7f-1-3p* | 71.3 |
|  |  |  | *hsa-let-7f-2-3p* | 5.0 |
|  |  |  | *hsa-miR-101-5p* | 45.5 |
|  |  |  | *hsa-miR-103a-2-5p* | 24.5 |
|  |  |  | *hsa-miR-10a-3p* | 49.5 |
|  |  |  | *hsa-miR-10b-3p* | 474.5 |
|  |  |  | *hsa-miR-125b-1-3p* | 33.9 |
|  |  |  | *hsa-miR-125b-2-3p* | 1146.2 |
|  |  |  | *hsa-miR-1271-3p* | 13.5 |
|  |  |  | *hsa-miR-1277-5p* | 44.0 |
|  |  |  | *hsa-miR-1306-3p* | 2.0 |
|  |  |  | *hsa-miR-1307-5p* | 0.5 |
|  |  |  | *hsa-miR-139-3p* | 157.5 |
|  |  |  | ***hsa-miR-145-3p*** | 22408.2 |
|  |  |  | *hsa-miR-1468-3p* | 1.0 |
|  |  |  | *hsa-miR-148a-5p* | 15.5 |
|  |  |  | *hsa-miR-153-5p* | 2.0 |
|  |  |  | *hsa-miR-15a-3p* | 0.5 |
|  |  |  | *hsa-miR-16-1-3p* | 0.5 |
|  |  |  | *hsa-miR-181a-3p* | 399.6 |
|  |  |  | *hsa-miR-181c-3p* | 19.0 |
|  |  |  | *hsa-miR-186-3p* | 35.5 |
|  |  |  | *hsa-miR-18a-3p* | 117.6 |
|  |  |  | *hsa-miR-190a-3p* | 2.0 |
|  |  |  | *hsa-miR-192-3p* | 8.5 |
|  |  |  | *hsa-miR-193b-5p* | 11.0 |
|  |  |  | *hsa-miR-194-3p* | 12.0 |
|  |  |  | *hsa-miR-19b-1-5p* | 23.9 |
|  |  |  | *hsa-miR-200c-5p* | 131.9 |
|  |  |  | *hsa-miR-205-3p* | 108.0 |
|  |  |  | *hsa-miR-20a-3p* | 2158.5 |
|  |  |  | *hsa-miR-212-3p* | 8.5 |
|  |  |  | *hsa-miR-214-5p* | 6.5 |
|  |  |  | *hsa-miR-221-5p* | 344.3 |
|  |  |  | *hsa-miR-223-5p* | 4.5 |
|  |  |  | *hsa-miR-23a-5p* | 8.5 |
|  |  |  | *hsa-miR-24-2-5p* | 22.0 |
|  |  |  | *hsa-miR-25-5p* | 6.5 |
|  |  |  | *hsa-miR-26a-2-3p* | 18.5 |
|  |  |  | *hsa-miR-26b-3p* | 8.0 |
|  |  |  | ***hsa-miR-29a-5p*** | 1080.5 |
|  |  |  | *hsa-miR-29b-1-5p* | 329.6 |
|  |  |  | ***hsa-miR-30a-3p*** | 17422.3 |
|  |  |  | *hsa-miR-30c-2-3p* | 995.1 |
|  |  |  | *hsa-miR-30d-3p* | 149.1 |
|  |  |  | *hsa-miR-31-3p* | 2.0 |
|  |  |  | *hsa-miR-329-3p* | 2.5 |
|  |  |  | *hsa-miR-335-3p* | 5.0 |
|  |  |  | *hsa-miR-33a-3p* | 133.7 |
|  |  |  | *hsa-miR-33b-3p* | 21.5 |
|  |  |  | *hsa-miR-34a-3p* | 5.0 |
|  |  |  | *hsa-miR-34c-3p* | 1.0 |
|  |  |  | *hsa-miR-361-3p* | 1317.6 |
|  |  |  | *hsa-miR-363-5p* | 11.0 |
|  |  |  | *hsa-miR-365b-5p* | 6.0 |
|  |  |  | *hsa-miR-370-5p* | 1.0 |
|  |  |  | *hsa-miR-376a-5p* | 26.9 |
|  |  |  | *hsa-miR-376c-3p* | 1935.9 |
|  |  |  | *hsa-miR-377-5p* | 0.5 |
|  |  |  | *hsa-miR-378a-5p* | 399.3 |
|  |  |  | *hsa-miR-379-3p* | 163.0 |
|  |  |  | *hsa-miR-381-5p* | 17.5 |
|  |  |  | *hsa-miR-382-3p* | 41.4 |
|  |  |  | *hsa-miR-412-5p* | 43.0 |
|  |  |  | *hsa-miR-485-3p* | 1.5 |
|  |  |  | *hsa-miR-487a-5p* | 38.9 |
|  |  |  | *hsa-miR-487b-5p* | 4.5 |
|  |  |  | *hsa-miR-490-5p* | 2.0 |
|  |  |  | *hsa-miR-491-3p* | 6.0 |
|  |  |  | *hsa-miR-493-5p* | 552.7 |
|  |  |  | *hsa-miR-497-3p* | 48.6 |
|  |  |  | *hsa-miR-500a-3p* | 1076.5 |
|  |  |  | *hsa-miR-505-5p* | 3.5 |
|  |  |  | *hsa-miR-532-3p* | 192.9 |
|  |  |  | *hsa-miR-541-5p* | 8.0 |
|  |  |  | *hsa-miR-628-3p* | 150.1 |
|  |  |  | *hsa-miR-652-5p* | 170.7 |
|  |  |  | *hsa-miR-670-3p* | 1.0 |
|  |  |  | *hsa-miR-671-3p* | 31.5 |
|  |  |  | *hsa-miR-7-1-3p* | 272.8 |
|  |  |  | *hsa-miR-876-3p* | 1.0 |
|  |  |  | *hsa-miR-885-3p* | 128.9 |
|  |  |  | *hsa-miR-92a-1-5p* | 26.5 |
|  |  |  | *hsa-miR-96-3p* | 7.5 |
|  |  |  | *hsa-miR-98-3p* | 5.0 |
|  |  |  | *mdo-miR-32-3p* | 231.5 |
|  |  |  | *mml-miR-106a-3p* | 2.0 |
|  |  |  | *mml-miR-20b-3p* | 33.1 |
|  |  |  | *mml-miR-452-3p* | 75.0 |
|  |  |  | *mml-miR-543-5p* | 34.5 |
|  |  |  | *mml-miR-6529-3p* | 22.0 |
|  |  |  | *mmu-let-7g-3p* | 0.5 |
|  |  |  | *mmu-let-7i-3p* | 18.9 |
|  |  |  | *mmu-miR-100-3p* | 162.7 |
|  |  |  | *mmu-miR-103-1-5p* | 2.0 |
|  |  |  | *mmu-miR-106b-3p* | 1571.8 |
|  |  |  | *mmu-miR-107-5p* | 1.5 |
|  |  |  | *mmu-miR-125a-3p* | 292.8 |
|  |  |  | *mmu-miR-127-5p* | 188.7 |
|  |  |  | *mmu-miR-128-1-5p* | 5.0 |
|  |  |  | *mmu-miR-1298-3p* | 2.5 |
|  |  |  | *mmu-miR-130b-5p* | 4.5 |
|  |  |  | *mmu-miR-132-5p* | 71.5 |
|  |  |  | *mmu-miR-133a-5p* | 8.0 |
|  |  |  | *mmu-miR-135a-1-3p* | 6.0 |
|  |  |  | *mmu-miR-135a-2-3p* | 19.0 |
|  |  |  | *mmu-miR-136-3p* | 837.0 |
|  |  |  | *mmu-miR-138-1-3p* | 4.5 |
|  |  |  | *mmu-miR-138-2-3p* | 4.5 |
|  |  |  | ***mmu-miR-140-5p*** | 3202.0 |
|  |  |  | *mmu-miR-143-5p* | 4.0 |
|  |  |  | *mmu-miR-144-5p* | 55.1 |
|  |  |  | *mmu-miR-146b-3p* | 98.8 |
|  |  |  | *mmu-miR-15b-3p* | 35.5 |
|  |  |  | *mmu-miR-16-2-3p* | 18.5 |
|  |  |  | *mmu-miR-181a-2-3p* | 1.0 |
|  |  |  | *mmu-miR-183-3p* | 48.9 |
|  |  |  | *mmu-miR-188-3p* | 52.1 |
|  |  |  | *mmu-miR-195a-3p* | 249.4 |
|  |  |  | *mmu-miR-200a-5p* | 645.9 |
|  |  |  | ***mmu-miR-200b-5p*** | 4542.6 |
|  |  |  | *mmu-miR-204-3p* | 28.5 |
|  |  |  | *mmu-miR-210-5p* | 0.5 |
|  |  |  | *mmu-miR-216a-3p* | 0.5 |
|  |  |  | *mmu-miR-218-1-3p* | 4.0 |
|  |  |  | *mmu-miR-27b-5p* | 217.7 |
|  |  |  | *mmu-miR-299a-3p* | 244.5 |
|  |  |  | *mmu-miR-29c-5p* | 465.8 |
|  |  |  | *mmu-miR-301a-5p* | 0.5 |
|  |  |  | *mmu-miR-3064-5p* | 12.5 |
|  |  |  | *mmu-miR-30c-1-3p* | 212.5 |
|  |  |  | ***mmu-miR-30e-3p*** | 8813.7 |
|  |  |  | *mmu-miR-409-3p* | 566.6 |
|  |  |  | *mmu-miR-410-5p* | 0.5 |
|  |  |  | *mmu-miR-411-3p* | 209.7 |
|  |  |  | *mmu-miR-431-3p* | 1.0 |
|  |  |  | *mmu-miR-542-3p* | 968.5 |
|  |  |  | *mmu-miR-744-3p* | 1.0 |
|  |  |  | *mmu-miR-874-5p* | 28.5 |
|  |  |  | *mmu-miR-99b-3p* | 98.0 |
|  |  |  | *oar-miR-1185-3p* | 41.0 |
|  |  |  | *oar-miR-154a-3p* | 15.5 |
|  |  |  | *oar-miR-154b-3p* | 79.4 |
|  |  |  | *oar-miR-3957-3p* | 1.0 |
|  |  |  | *oar-miR-3958-5p* | 1.0 |
|  |  |  | *oar-miR-411b-3p* | 1.5 |
|  |  |  | *oar-miR-494-5p* | 4.0 |
|  |  |  | *oar-miR-544-5p* | 43.0 |
|  |  |  | *rno-miR-148b-5p* | 6.5 |
|  |  |  | *rno-miR-324-3p* | 94.0 |
|  |  |  | *rno-miR-330-5p* | 26.9 |
|  |  |  | *rno-miR-338-5p* | 140.5 |
|  |  |  | ***rno-miR-340-5p*** | 7720.7 |
|  |  |  | *rno-miR-421-5p* | 4.0 |
|  |  |  | *ssc-miR-181d-3p* | 1.0 |
|  |  |  | *ssc-miR-374a-3p* | 1915.0 |
|  |  |  | *ssc-miR-374b-3p* | 119.9 |
|  |  |  | *tgu-let-7b-3p* | 42.3 |
|  |  |  | *tgu-miR-194-3p* | 3.0 |

In bold: common miRNA to both species with expression above 100 RPM.

**C.**

| **miRNA** | **Mouse** | **AGO2** | **miRNA** | **Bovine** |
| --- | --- | --- | --- | --- |
| *mmu-1_1164* | 0.0 | 29.5 | *bta-1_1147* | 1.5 |
| *mmu-1_1220* | 40.9 | 12.0 | *bta-1_1247* | 1.0 |
| *mmu-1_1220* | 0.0 | 6.0 | *bta-1_1257* | 4.5 |
| *mmu-1_1287* | 0.0 | 35.7 | *bta-1_1291* | 1.0 |
| *mmu-1_1349* | 0.0 | 4.7 | *bta-1_1291* | 1.5 |
| *mmu-1_1498* | 0.0 | 6.0 | *bta-1_1509* | 36.2 |
| *mmu-1_1498* | 0.0 | 1.3 | *bta-1_1509* | 14.9 |
| *mmu-1_159* | 0.0 | 6.0 | *bta-1_1656* | 1.5 |
| *mmu-1_1600* | 0.0 | 51.2 | *bta-1_189* | 12.5 |
| *mmu-1_1606* | 0.0 | 13.2 | *bta-1_336* | 12.5 |
| *mmu-1_1752* | 0.0 | 16.3 | *bta-1_383* | 1.0 |
| *mmu-1_1789* | 0.0 | 5.3 | *bta-1_46* | 1.0 |
| *mmu-1_1789* | 0.0 | 1.3 | *bta-1_500* | 2.0 |
| *mmu-1_1880* | 0.0 | 28.7 | *bta-1_635* | 13.5 |
| *mmu-1_1982* | 4.2 | 0.0 | *bta-1_650* | 6.0 |
| *mmu-1_2030* | 0.0 | 31.8 | *bta-1_726* | 1.0 |
| *mmu-1_2153* | 0.0 | 6.0 | *bta-1_726* | 0.5 |
| *mmu-1_2198* | 0.0 | 4.7 | *bta-1_821* | 6.0 |
| *mmu-1_2233* | 0.0 | 4.7 | *bta-1_846* | 58.5 |
| *mmu-1_2285* | 0.0 | 8.7 | *bta-1_846* | 1.5 |
| *mmu-1_230* | 0.3 | 12.4 | *bta-1_854* | 1.5 |
| *mmu-1_2303* | 0.0 | 70.6 | *bta-1_855* | 1.0 |
| *mmu-1_2378* | 0.0 | 31.0 | *bta-1_876* | 10.0 |
| *mmu-1_2378* | 0.0 | 1.3 | *bta-1_876* | 3.5 |
| *mmu-1_252* | 0.0 | 24.5 | *bta-1_915* | 10.0 |
| *mmu-1_2535* | 0.0 | 14.7 | *bta-1_915* | 0.5 |
| *mmu-1_2549* | 0.0 | 42.7 | *bta-10_1732* | 48.0 |
| *mmu-1_267* | 1.0 | 4.7 | *bta-10_1742* | 1.0 |
| *mmu-1_2697* | 0.0 | 37.2 | *bta-10_1780* | 0.5 |
| *mmu-1_2776* | 0.0 | 36.5 | *bta-10_1831* | 1.5 |
| *mmu-1_2777* | 0.0 | 24.0 | *bta-10_1842* | 1.5 |
| *mmu-1_2777* | 0.0 | 1.3 | *bta-10_1846* | 2.0 |
| *mmu-1_2785* | 0.0 | 6.0 | *bta-10_2099* | 2.0 |
| *mmu-1_2823* | 0.0 | 58.9 | *bta-10_2213* | 0.5 |
| *mmu-1_2866* | 0.0 | 31.0 | *bta-10_2288* | 1.5 |
| *mmu-1_2981* | 0.0 | 4.7 | *bta-10_2288* | 0.5 |
| *mmu-1_326* | 0.4 | 62.3 | *bta-10_2312* | 6.5 |
| *mmu-1_329* | 0.0 | 24.8 | *bta-10_2420* | 22.6 |
| *mmu-1_340* | 0.0 | 4.7 | *bta-10_2498* | 3.5 |
| *mmu-1_390* | 0.0 | 48.1 | *bta-10_2520* | 1.5 |
| *mmu-1_517* | 1.4 | 35.1 | *bta-10_2529* | 89.1 |
| *mmu-1_523* | 0.0 | 24.0 | *bta-10_2529* | 0.5 |
| *mmu-1_566* | 0.0 | 52.7 | *bta-10_2543* | 1.0 |
| *mmu-1_574* | 0.0 | 23.3 | *bta-10_2600* | 56.5 |
| *mmu-1_576* | 24.1 | 183.4 | *bta-10_2600* | 2.0 |
| *mmu-1_582* | 0.0 | 5.3 | *bta-10_2603* | 3.5 |
| *mmu-1_626* | 0.0 | 5.3 | *bta-10_2612* | 1.0 |
| *mmu-1_649* | 0.0 | 22.5 | *bta-10_2660* | 2.5 |
| *mmu-1_649* | 0.0 | 6.0 | *bta-10_2670* | 0.5 |
| *mmu-1_916* | 0.0 | 8.0 | *bta-10_2697* | 2.5 |
| *mmu-1_964* | 0.0 | 42.7 | *bta-10_2718* | 1.0 |
| *mmu-1_988* | 0.0 | 7.8 | *bta-10_2736* | 7789.7 |
| *mmu-1_998* | 0.0 | 4.7 | *bta-10_2786* | 1.0 |
| *mmu-10_3161* | 0.0 | 69.0 | *bta-10_2799* | 1.0 |
| *mmu-10_3216* | 0.0 | 257.6 | *bta-10_3147* | 1.0 |
| *mmu-10_3228* | 0.0 | 14.7 | *bta-10_3152* | 1.0 |
| *mmu-10_3249* | 0.0 | 5.3 | *bta-10_3162* | 1.0 |
| *mmu-10_3281* | 0.0 | 40.3 | *bta-10_3319* | 3.5 |
| *mmu-10_3288* | 0.0 | 19.4 | *bta-10_3319* | 1.0 |
| *mmu-10_3288* | 0.0 | 0.7 | *bta-10_3341* | 13.5 |
| *mmu-10_3298* | 0.0 | 4.7 | *bta-11_3412* | 18.0 |
| *mmu-10_3331* | 0.0 | 23.3 | *bta-11_3424* | 1.0 |
| *mmu-10_3346* | 0.0 | 45.0 | *bta-11_3629* | 10.0 |
| *mmu-10_3347* | 0.0 | 45.0 | *bta-11_3676* | 1.0 |
| *mmu-10_3450* | 0.0 | 47.9 | *bta-11_3731* | 5.5 |
| *mmu-10_3454* | 0.0 | 28.7 | *bta-11_3738* | 1.0 |
| *mmu-10_3468* | 0.0 | 21.7 | *bta-11_3738* | 0.5 |
| *mmu-10_3594* | 0.0 | 55.8 | *bta-11_3777* | 99.3 |
| *mmu-10_3594* | 0.0 | 0.7 | *bta-11_3866* | 6.0 |
| *mmu-10_3701* | 0.0 | 24.0 | *bta-11_3885* | 1.0 |
| *mmu-10_3708* | 0.0 | 20.0 | *bta-11_3885* | 0.5 |
| *mmu-10_3730* | 0.0 | 35.7 | *bta-11_4070* | 2.0 |
| *mmu-10_3756* | 0.0 | 73.7 | *bta-11_4097* | 1.5 |
| *mmu-10_3769* | 0.0 | 42.7 | *bta-11_4133* | 14.5 |
| *mmu-10_3798* | 0.0 | 4.7 | *bta-11_4133* | 0.5 |
| *mmu-10_3802* | 0.0 | 39.6 | *bta-11_4317* | 7.5 |
| *mmu-10_3919* | 0.0 | 54.3 | *bta-11_4317* | 1.5 |
| *mmu-10_3986* | 0.0 | 9.3 | *bta-11_4401* | 1.0 |
| *mmu-10_4058* | 0.0 | 4.7 | *bta-11_4486* | 12.0 |
| *mmu-10_4073* | 0.0 | 24.3 | *bta-11_4520* | 1.0 |
| *mmu-10_4073* | 0.0 | 2.7 | *bta-11_4679* | 3.0 |
| *mmu-10_4106* | 0.0 | 49.6 | *bta-11_4702* | 1.5 |
| *mmu-10_4106* | 0.0 | 4.0 | *bta-11_4702* | 0.5 |
| *mmu-10_4234* | 0.0 | 8.5 | *bta-11_4804* | 2.0 |
| *mmu-10_4305* | 0.0 | 52.0 | *bta-11_4808* | 0.5 |
| *mmu-10_4376* | 0.0 | 50.4 | *bta-11_4895* | 1.0 |
| *mmu-10_4415* | 0.0 | 9.3 | *bta-11_5023* | 0.5 |
| *mmu-10_4417* | 0.0 | 10.1 | *bta-11_5130* | 1.0 |
| *mmu-10_4424* | 0.0 | 4.7 | *bta-11_5130* | 0.5 |
| *mmu-10_4460* | 0.0 | 22.5 | *bta-11_5139* | 1.0 |
| *mmu-10_4489* | 0.0 | 4.7 | *bta-11_5165* | 1.0 |
| *mmu-10_4500* | 0.0 | 70.6 | *bta-11_5261* | 5.0 |
| *mmu-10_4600* | 0.0 | 11.6 | *bta-11_5290* | 1.0 |
| *mmu-10_4615* | 0.0 | 6.7 | *bta-11_5310* | 1.0 |
| *mmu-10_4668* | 0.0 | 47.3 | *bta-11_5342* | 1.0 |
| *mmu-10_4674* | 0.0 | 6.0 | *bta-12_5391* | 3.5 |
| *mmu-10_4682* | 0.0 | 14.7 | *bta-12_5397* | 1.5 |
| *mmu-10_4692* | 0.0 | 26.4 | *bta-12_5486* | 48.6 |
| *mmu-10_4692* | 0.0 | 2.7 | *bta-12_5486* | 1.5 |
| *mmu-10_4841* | 0.0 | 16.5 | *bta-12_5501* | 140.9 |
| *mmu-10_4875* | 0.0 | 5.3 | *bta-12_5551* | 1.0 |
| *mmu-10_4979* | 5.8 | 7.3 | *bta-12_5596* | 84.2 |
| *mmu-10_4979* | 0.0 | 0.7 | *bta-12_5676* | 1.5 |
| *mmu-10_5021* | 0.7 | 24.6 | *bta-12_5676* | 0.5 |
| *mmu-10_5023* | 0.0 | 24.0 | *bta-12_5781* | 3.0 |
| *mmu-10_5033* | 0.0 | 4.7 | *bta-12_5826* | 9.0 |
| *mmu-11_5157* | 0.0 | 4.7 | *bta-12_5858* | 404.3 |
| *mmu-11_5306* | 0.0 | 17.8 | *bta-12_5865* | 3.5 |
| *mmu-11_5307* | 0.0 | 6.0 | *bta-12_5879* | 1.0 |
| *mmu-11_5533* | 0.0 | 48.4 | *bta-12_5909* | 3.0 |
| *mmu-11_5579* | 0.0 | 22.5 | *bta-12_6057* | 35.9 |
| *mmu-11_5656* | 0.0 | 4.7 | *bta-12_6071* | 4.5 |
| *mmu-11_5722* | 0.0 | 5.3 | *bta-12_6071* | 0.5 |
| *mmu-11_5734* | 0.0 | 5.3 | *bta-12_6093* | 0.5 |
| *mmu-11_5752* | 0.0 | 20.2 | *bta-12_6093* | 0.5 |
| *mmu-11_5777* | 0.0 | 12.0 | *bta-12_6200* | 1.0 |
| *mmu-11_5787* | 14.4 | 33.7 | *bta-12_6203* | 1.0 |
| *mmu-11_5787* | 1.4 | 1.3 | *bta-13_6276* | 15.0 |
| *mmu-11_5790* | 0.0 | 8.5 | *bta-13_6279* | 2.0 |
| *mmu-11_5790* | 0.3 | 1.3 | *bta-13_6290* | 4.0 |
| *mmu-11_5828* | 0.6 | 76.5 | *bta-13_6374* | 0.5 |
| *mmu-11_5828* | 0.6 | 0.0 | *bta-13_6408* | 36.0 |
| *mmu-11_5843* | 8.1 | 36.3 | *bta-13_6423* | 1.0 |
| *mmu-11_5847* | 0.0 | 10.9 | *bta-13_6492* | 4.0 |
| *mmu-11_5934* | 0.0 | 34.9 | *bta-13_6519* | 20.6 |
| *mmu-11_5945* | 0.3 | 22.1 | *bta-13_6519* | 2.5 |
| *mmu-11_5995* | 0.0 | 50.2 | *bta-13_6655* | 6.0 |
| *mmu-11_6031* | 0.0 | 7.3 | *bta-13_6674* | 1.0 |
| *mmu-11_6031* | 0.0 | 2.7 | *bta-13_6813* | 2.5 |
| *mmu-11_6050* | 0.0 | 5.3 | *bta-13_7035* | 20.0 |
| *mmu-11_6063* | 0.0 | 22.5 | *bta-13_7103* | 19.0 |
| *mmu-11_6132* | 0.0 | 3.3 | *bta-13_7106* | 1.0 |
| *mmu-11_6158* | 0.0 | 6.0 | *bta-13_7130* | 4.0 |
| *mmu-11_6164* | 3.6 | 0.0 | *bta-13_7156* | 3.5 |
| *mmu-11_6187* | 0.0 | 10.0 | *bta-13_7156* | 3.5 |
| *mmu-11_6248* | 0.0 | 18.6 | *bta-13_7170* | 43.5 |
| *mmu-11_6360* | 0.0 | 22.5 | *bta-13_7192* | 1.0 |
| *mmu-11_6364* | 0.0 | 76.8 | *bta-13_7197* | 9.5 |
| *mmu-11_6456* | 0.0 | 50.4 | *bta-13_7201* | 1.5 |
| *mmu-11_6469* | 92.1 | 2.7 | *bta-13_7201* | 0.5 |
| *mmu-11_6469* | 0.0 | 4.7 | *bta-13_7241* | 1.5 |
| *mmu-11_6501* | 1.6 | 12.6 | *bta-13_7285* | 7.9 |
| *mmu-11_6554* | 0.0 | 12.8 | *bta-13_7341* | 3.5 |
| *mmu-11_6558* | 0.0 | 44.2 | *bta-13_7341* | 0.5 |
| *mmu-11_6570* | 0.0 | 39.3 | *bta-13_7348* | 24.5 |
| *mmu-11_6604* | 0.0 | 4.7 | *bta-13_7364* | 2.5 |
| *mmu-11_6607* | 0.0 | 6.7 | *bta-13_7394* | 482.5 |
| *mmu-11_6623* | 3204.2 | 64.6 | *bta-13_7436* | 1.5 |
| *mmu-11_6623* | 1.9 | 0.0 | *bta-13_7459* | 4.0 |
| *mmu-11_6703* | 0.0 | 6.7 | *bta-13_7459* | 1.0 |
| *mmu-11_6705* | 0.0 | 14.0 | *bta-13_7477* | 1.5 |
| *mmu-11_6746* | 0.0 | 17.1 | *bta-13_7521* | 1.5 |
| *mmu-11_6774* | 0.0 | 6.0 | *bta-13_7521* | 0.5 |
| *mmu-11_6788* | 126.6 | 239.1 | *bta-13_7536* | 28.0 |
| *mmu-11_6788* | 3.5 | 10.6 | *bta-14_7825* | 0.5 |
| *mmu-11_6799* | 0.0 | 6.7 | *bta-14_7918* | 0.5 |
| *mmu-11_6866* | 3.1 | 2.7 | *bta-14_7948* | 1.5 |
| *mmu-11_6867* | 0.0 | 45.0 | *bta-14_8062* | 1.0 |
| *mmu-11_6957* | 0.0 | 4.7 | *bta-14_8089* | 1.0 |
| *mmu-11_7015* | 0.0 | 5.3 | *bta-14_8128* | 1.0 |
| *mmu-11_7083* | 0.3 | 52.2 | *bta-14_8132* | 25.5 |
| *mmu-11_7098* | 1.4 | 605.2 | *bta-14_8150* | 3.0 |
| *mmu-11_7373* | 0.0 | 19.9 | *bta-14_8160* | 1.5 |
| *mmu-11_7373* | 0.0 | 4.0 | *bta-14_8262* | 14.5 |
| *mmu-11_7427* | 0.0 | 29.5 | *bta-14_8296* | 1.5 |
| *mmu-11_7430* | 0.0 | 7.2 | *bta-14_8516* | 1.0 |
| *mmu-11_7581* | 0.0 | 15.5 | *bta-14_8561* | 2.0 |
| *mmu-11_7599* | 0.0 | 5.3 | *bta-14_8629* | 5.5 |
| *mmu-11_7611* | 0.0 | 22.5 | *bta-14_8643* | 336.0 |
| *mmu-11_7616* | 0.0 | 7.3 | *bta-14_8643* | 1.0 |
| *mmu-11_7702* | 0.0 | 9.3 | *bta-15_8740* | 3.0 |
| *mmu-11_7768* | 0.0 | 18.6 | *bta-15_8740* | 0.5 |
| *mmu-11_7784* | 0.0 | 56.6 | *bta-15_8930* | 2.0 |
| *mmu-11_7803* | 0.0 | 52.0 | *bta-15_8949* | 36.4 |
| *mmu-11_7857* | 0.0 | 6.0 | *bta-15_9006* | 2.0 |
| *mmu-11_7939* | 0.0 | 60.4 | *bta-15_9006* | 0.5 |
| *mmu-11_7971* | 0.0 | 40.3 | *bta-15_9039* | 1.5 |
| *mmu-11_8029* | 0.0 | 4.7 | *bta-15_9271* | 3.5 |
| *mmu-11_8052* | 0.0 | 6.0 | *bta-15_9300* | 5.0 |
| *mmu-11_8102* | 0.0 | 34.9 | *bta-15_9483* | 16.0 |
| *mmu-11_8102* | 0.0 | 4.0 | *bta-15_9524* | 2.0 |
| *mmu-11_8338* | 0.0 | 22.5 | *bta-15_9626* | 4.5 |
| *mmu-11_8389* | 0.0 | 6.0 | *bta-15_9760* | 1.5 |
| *mmu-11_8449* | 0.0 | 48.1 | *bta-15_9894* | 5.5 |
| *mmu-11_8497* | 0.0 | 20.2 | *bta-16_10029* | 165.2 |
| *mmu-11_8552* | 0.0 | 4.7 | *bta-16_10094* | 2047.2 |
| *mmu-11_8639* | 0.0 | 49.6 | *bta-16_10096* | 1.0 |
| *mmu-11_8696* | 0.0 | 39.6 | *bta-16_10098* | 1.0 |
| *mmu-11_8698* | 0.0 | 45.0 | *bta-16_10099* | 14.5 |
| *mmu-11_8860* | 0.0 | 42.1 | *bta-16_10129* | 1.0 |
| *mmu-11_8860* | 0.7 | 0.0 | *bta-16_10326* | 1.0 |
| *mmu-12_10033* | 0.0 | 63.6 | *bta-16_10338* | 1.0 |
| *mmu-12_10067* | 0.0 | 6.0 | *bta-16_10347* | 3.0 |
| *mmu-12_10152* | 0.0 | 36.5 | *bta-16_10347* | 2.0 |
| *mmu-12_10152* | 0.0 | 3.3 | *bta-16_10413* | 21.5 |
| *mmu-12_10240* | 0.0 | 11.3 | *bta-16_10415* | 1.0 |
| *mmu-12_10271* | 0.0 | 29.5 | *bta-16_10452* | 1.0 |
| *mmu-12_10289* | 0.0 | 38.0 | *bta-16_10498* | 22.0 |
| *mmu-12_10464* | 0.0 | 2.7 | *bta-16_10630* | 2.0 |
| *mmu-12_10472* | 0.0 | 5.3 | *bta-16_10630* | 1.5 |
| *mmu-12_10472* | 0.0 | 2.0 | *bta-16_10667* | 1627.7 |
| *mmu-12_10514* | 0.0 | 17.1 | *bta-16_10674* | 0.5 |
| *mmu-12_10601* | 0.0 | 41.9 | *bta-16_10773* | 1.0 |
| *mmu-12_10633* | 0.0 | 6.0 | *bta-16_10773* | 0.5 |
| *mmu-12_10719* | 0.0 | 5.3 | *bta-16_10781* | 2.5 |
| *mmu-12_10834* | 0.0 | 6.0 | *bta-16_10856* | 0.5 |
| *mmu-12_10837* | 0.0 | 6.7 | *bta-16_10868* | 2.0 |
| *mmu-12_10863* | 0.0 | 20.9 | *bta-16_10912* | 0.5 |
| *mmu-12_10872* | 56.0 | 6.0 | *bta-16_10929* | 3.0 |
| *mmu-12_10889* | 0.0 | 8.0 | *bta-16_10980* | 1.0 |
| *mmu-12_9150* | 0.0 | 65.9 | *bta-16_11038* | 3.0 |
| *mmu-12_9417* | 0.0 | 4.7 | *bta-16_11039* | 3.5 |
| *mmu-12_9511* | 0.0 | 20.2 | *bta-16_11039* | 0.5 |
| *mmu-12_9580* | 0.0 | 6.7 | *bta-16_11041* | 1.0 |
| *mmu-12_9580* | 0.6 | 0.0 | *bta-16_11084* | 14.0 |
| *mmu-12_9634* | 0.0 | 19.4 | *bta-16_11084* | 21.5 |
| *mmu-12_9652* | 0.0 | 80.7 | *bta-16_11215* | 2.0 |
| *mmu-12_9670* | 0.0 | 43.4 | *bta-16_11222* | 1.0 |
| *mmu-12_9733* | 0.0 | 35.7 | *bta-16_11252* | 1.5 |
| *mmu-12_9894* | 0.0 | 32.6 | *bta-16_9984* | 1.0 |
| *mmu-12_9931* | 0.0 | 26.4 | *bta-17_11288* | 7.0 |
| *mmu-12_9959* | 0.0 | 6.0 | *bta-17_11382* | 1.0 |
| *mmu-12_9971* | 0.0 | 4.7 | *bta-17_11474* | 22.0 |
| *mmu-13_11049* | 0.0 | 14.0 | *bta-17_11654* | 13.0 |
| *mmu-13_11107* | 11.9 | 60.0 | *bta-17_11761* | 4.5 |
| *mmu-13_11181* | 0.0 | 48.9 | *bta-17_11780* | 1.0 |
| *mmu-13_11245* | 1.5 | 2.7 | *bta-17_11802* | 2.0 |
| *mmu-13_11301* | 0.0 | 6.7 | *bta-17_11869* | 2.5 |
| *mmu-13_11333* | 50.7 | 12.1 | *bta-17_12013* | 0.5 |
| *mmu-13_11454* | 0.0 | 6.0 | *bta-17_12013* | 1.5 |
| *mmu-13_11493* | 0.0 | 48.1 | *bta-17_12035* | 12.5 |
| *mmu-13_11638* | 2.7 | 3.3 | *bta-17_12105* | 1.0 |
| *mmu-13_11658* | 0.0 | 12.8 | *bta-17_12113* | 3.5 |
| *mmu-13_11802* | 0.0 | 26.4 | *bta-17_12113* | 2.0 |
| *mmu-13_12158* | 0.0 | 25.6 | *bta-17_12128* | 3.5 |
| *mmu-13_12174* | 0.0 | 60.3 | *bta-17_12143* | 4.0 |
| *mmu-13_12207* | 0.0 | 5.3 | *bta-17_12158* | 4.0 |
| *mmu-13_12241* | 0.0 | 27.9 | *bta-17_12301* | 2.0 |
| *mmu-13_12268* | 2.0 | 4.7 | *bta-17_12392* | 1.0 |
| *mmu-13_12346* | 0.0 | 18.6 | *bta-17_12481* | 1.0 |
| *mmu-13_12391* | 0.0 | 17.1 | *bta-18_12866* | 0.5 |
| *mmu-13_12474* | 0.0 | 6.7 | *bta-18_12920* | 3.0 |
| *mmu-13_12482* | 0.0 | 5.3 | *bta-18_12938* | 0.5 |
| *mmu-13_12482* | 0.0 | 4.7 | *bta-18_13000* | 1.0 |
| *mmu-13_12504* | 0.0 | 5.3 | *bta-18_13048* | 1.0 |
| *mmu-13_12504* | 0.0 | 2.7 | *bta-18_13152* | 15.0 |
| *mmu-13_12547* | 2.8 | 0.0 | *bta-18_13284* | 1.0 |
| *mmu-13_12634* | 0.0 | 29.5 | *bta-18_13349* | 2.0 |
| *mmu-13_12734* | 0.0 | 35.6 | *bta-18_13469* | 1.5 |
| *mmu-13_12872* | 0.0 | 34.9 | *bta-18_13488* | 21.0 |
| *mmu-14_13028* | 0.0 | 5.3 | *bta-18_13603* | 1.0 |
| *mmu-14_13125* | 0.0 | 26.0 | *bta-18_13649* | 1.0 |
| *mmu-14_13176* | 0.0 | 76.5 | *bta-18_13664* | 1.5 |
| *mmu-14_13197* | 0.0 | 14.7 | *bta-18_13664* | 0.5 |
| *mmu-14_13226* | 3.5 | 0.0 | *bta-18_13763* | 2.0 |
| *mmu-14_13246* | 0.0 | 9.3 | *bta-18_13769* | 3.5 |
| *mmu-14_13315* | 0.0 | 5.3 | *bta-18_13909* | 10.0 |
| *mmu-14_13379* | 0.3 | 6.0 | *bta-18_13924* | 4.0 |
| *mmu-14_13439* | 0.0 | 27.3 | *bta-19_14244* | 1.0 |
| *mmu-14_13496* | 0.0 | 24.8 | *bta-19_14363* | 0.5 |
| *mmu-14_13497* | 0.0 | 27.9 | *bta-19_14534* | 1.0 |
| *mmu-14_13516* | 0.0 | 20.9 | *bta-19_14585* | 1.0 |
| *mmu-14_13655* | 0.0 | 45.8 | *bta-19_14622* | 1.0 |
| *mmu-14_13792* | 0.0 | 4.7 | *bta-19_14695* | 0.5 |
| *mmu-14_13842* | 0.0 | 33.4 | *bta-19_14704* | 3.5 |
| *mmu-14_13842* | 0.0 | 10.6 | *bta-19_14704* | 0.5 |
| *mmu-14_13985* | 0.0 | 4.7 | *bta-19_14812* | 1.5 |
| *mmu-14_13985* | 0.0 | 0.8 | *bta-19_14850* | 1.5 |
| *mmu-14_14008* | 0.0 | 34.1 | *bta-19_14883* | 25.4 |
| *mmu-14_14065* | 0.0 | 29.5 | *bta-19_14902* | 58.6 |
| *mmu-14_14065* | 0.0 | 0.7 | *bta-19_14929* | 3.5 |
| *mmu-14_14160* | 0.0 | 51.2 | *bta-19_15011* | 1.5 |
| *mmu-15_14628* | 0.0 | 4.7 | *bta-19_15145* | 3.0 |
| *mmu-15_14628* | 0.0 | 2.0 | *bta-19_15145* | 1.5 |
| *mmu-15_14678* | 0.0 | 10.1 | *bta-19_15171* | 0.5 |
| *mmu-15_14738* | 0.0 | 5.3 | *bta-19_15181* | 10.0 |
| *mmu-15_14760* | 0.0 | 4.7 | *bta-19_15227* | 5.5 |
| *mmu-15_14880* | 0.0 | 27.9 | *bta-19_15270* | 77.6 |
| *mmu-15_14905* | 0.0 | 20.9 | *bta-19_15280* | 4.0 |
| *mmu-15_14943* | 14.8 | 5.3 | *bta-19_15355* | 2.0 |
| *mmu-15_15015* | 0.0 | 4.7 | *bta-19_15355* | 0.5 |
| *mmu-15_15055* | 0.0 | 11.6 | *bta-19_15373* | 2.5 |
| *mmu-15_15059* | 0.0 | 45.0 | *bta-19_15373* | 0.5 |
| *mmu-15_15116* | 0.0 | 33.3 | *bta-19_15455* | 1.5 |
| *mmu-15_15131* | 0.0 | 6.7 | *bta-19_15595* | 1.5 |
| *mmu-15_15172* | 0.0 | 25.3 | *bta-19_15739* | 1.5 |
| *mmu-15_15172* | 0.0 | 2.7 | *bta-19_15780* | 5.0 |
| *mmu-15_15173* | 0.0 | 8.0 | *bta-19_15788* | 6.5 |
| *mmu-15_15258* | 0.0 | 5.3 | *bta-19_15792* | 1.0 |
| *mmu-15_15267* | 17.3 | 853.1 | *bta-19_16009* | 0.5 |
| *mmu-15_15267* | 0.6 | 0.0 | *bta-19_16053* | 1.5 |
| *mmu-15_15363* | 0.0 | 62.8 | *bta-19_16066* | 5.5 |
| *mmu-15_15419* | 0.0 | 14.7 | *bta-19_16066* | 1.0 |
| *mmu-15_15733* | 0.0 | 55.0 | *bta-19_16103* | 3.0 |
| *mmu-15_15765* | 0.0 | 27.9 | *bta-19_16108* | 1.5 |
| *mmu-15_15779* | 0.0 | 5.3 | *bta-19_16218* | 7.5 |
| *mmu-15_15860* | 0.0 | 8.7 | *bta-19_16218* | 1.0 |
| *mmu-15_15895* | 0.0 | 45.6 | *bta-19_16257* | 1.0 |
| *mmu-15_15968* | 0.0 | 6.7 | *bta-19_16317* | 2.5 |
| *mmu-15_16029* | 0.0 | 48.9 | *bta-19_16336* | 10.6 |
| *mmu-15_16031* | 0.0 | 5.3 | *bta-2_16430* | 80.5 |
| *mmu-15_16172* | 0.0 | 45.8 | *bta-2_16430* | 2.0 |
| *mmu-15_16194* | 0.0 | 52.5 | *bta-2_16525* | 1.0 |
| *mmu-15_16194* | 0.0 | 2.0 | *bta-2_16698* | 22.0 |
| *mmu-15_16333* | 0.0 | 6.7 | *bta-2_16930* | 4.0 |
| *mmu-15_16368* | 0.0 | 33.3 | *bta-2_16998* | 1.0 |
| *mmu-15_16436* | 0.0 | 25.6 | *bta-2_17037* | 1.5 |
| *mmu-15_16477* | 0.0 | 6.0 | *bta-2_17037* | 0.5 |
| *mmu-15_16513* | 0.0 | 27.1 | *bta-2_17125* | 2.0 |
| *mmu-15_16519* | 0.0 | 8.7 | *bta-2_17125* | 1.0 |
| *mmu-15_16566* | 0.0 | 5.3 | *bta-2_17144* | 1.5 |
| *mmu-16_16750* | 0.0 | 8.0 | *bta-2_17274* | 1.0 |
| *mmu-16_16757* | 0.0 | 38.0 | *bta-2_17280* | 1.0 |
| *mmu-16_16835* | 0.0 | 18.5 | *bta-2_17409* | 3.5 |
| *mmu-16_16915* | 0.0 | 47.3 | *bta-2_17581* | 14.4 |
| *mmu-16_16934* | 0.0 | 7.3 | *bta-2_17581* | 6.5 |
| *mmu-16_16934* | 0.3 | 0.0 | *bta-2_17742* | 2.5 |
| *mmu-16_16936* | 0.0 | 20.2 | *bta-2_17742* | 1.0 |
| *mmu-16_16962* | 0.4 | 4.7 | *bta-2_17747* | 1.0 |
| *mmu-16_16977* | 0.0 | 23.3 | *bta-2_17870* | 1.0 |
| *mmu-16_16985* | 0.0 | 30.2 | *bta-2_17994* | 15.5 |
| *mmu-16_17174* | 0.0 | 25.6 | *bta-2_17994* | 1.0 |
| *mmu-16_17219* | 0.0 | 56.6 | *bta-2_18067* | 1.0 |
| *mmu-16_17506* | 0.0 | 56.6 | *bta-2_18081* | 9.0 |
| *mmu-16_17542* | 0.7 | 14.5 | *bta-2_18081* | 0.5 |
| *mmu-16_17559* | 0.0 | 4.7 | *bta-2_18084* | 14.6 |
| *mmu-16_17579* | 0.0 | 33.3 | *bta-2_18089* | 9.6 |
| *mmu-16_17579* | 0.0 | 3.3 | *bta-2_18232* | 1.0 |
| *mmu-16_17607* | 0.0 | 7.3 | *bta-20_18290* | 1.0 |
| *mmu-16_17647* | 0.0 | 29.5 | *bta-20_18314* | 0.5 |
| *mmu-16_17647* | 0.0 | 2.7 | *bta-20_18345* | 1.0 |
| *mmu-16_17659* | 0.0 | 4.7 | *bta-20_18429* | 6.5 |
| *mmu-16_17838* | 0.0 | 20.9 | *bta-20_18500* | 1.0 |
| *mmu-16_17933* | 0.0 | 45.8 | *bta-20_18534* | 0.5 |
| *mmu-16_18195* | 0.0 | 17.1 | *bta-20_18562* | 3.0 |
| *mmu-16_18195* | 0.0 | 0.7 | *bta-20_18593* | 6.0 |
| *mmu-16_18199* | 0.0 | 8.5 | *bta-20_18593* | 0.5 |
| *mmu-16_18214* | 0.0 | 74.5 | *bta-20_18740* | 6.0 |
| *mmu-16_18230* | 0.0 | 26.4 | *bta-21_19032* | 11.0 |
| *mmu-16_18230* | 0.0 | 0.7 | *bta-21_19032* | 1.0 |
| *mmu-16_18263* | 0.0 | 8.7 | *bta-21_19213* | 1.0 |
| *mmu-16_18294* | 0.0 | 6.0 | *bta-21_19303* | 5.5 |
| *mmu-16_18310* | 0.0 | 34.1 | *bta-21_19354* | 113.4 |
| *mmu-17_18361* | 0.0 | 6.0 | *bta-21_19354* | 43.1 |
| *mmu-17_18375* | 0.0 | 40.3 | *bta-21_19450* | 3.5 |
| *mmu-17_18451* | 9.9 | 22.4 | *bta-21_19463* | 1.5 |
| *mmu-17_18451* | 0.3 | 4.0 | *bta-21_19497* | 4.0 |
| *mmu-17_18584* | 0.0 | 45.0 | *bta-21_19497* | 0.5 |
| *mmu-17_18653* | 0.0 | 4.7 | *bta-21_19563* | 1.0 |
| *mmu-17_18707* | 0.0 | 7.3 | *bta-21_19585* | 2.0 |
| *mmu-17_18732* | 0.0 | 8.0 | *bta-21_19593* | 24.0 |
| *mmu-17_18766* | 0.0 | 9.3 | *bta-21_19636* | 15.0 |
| *mmu-17_18807* | 0.0 | 16.3 | *bta-21_19636* | 2.5 |
| *mmu-17_19104* | 0.0 | 7.3 | *bta-21_20033* | 63.4 |
| *mmu-17_19106* | 0.0 | 50.4 | *bta-21_20146* | 76.7 |
| *mmu-17_19141* | 0.0 | 22.5 | *bta-21_20146* | 9.5 |
| *mmu-17_19161* | 0.0 | 6.0 | *bta-21_20150* | 5.0 |
| *mmu-17_19172* | 0.0 | 4.7 | *bta-21_20150* | 0.5 |
| *mmu-17_19245* | 0.0 | 4.7 | *bta-21_20156* | 1.5 |
| *mmu-17_19248* | 0.0 | 20.9 | *bta-22_20208* | 10.0 |
| *mmu-17_19385* | 0.0 | 8.7 | *bta-22_20230* | 10.0 |
| *mmu-17_19402* | 0.0 | 27.1 | *bta-22_20298* | 2.0 |
| *mmu-17_19502* | 0.0 | 14.0 | *bta-22_20329* | 1.0 |
| *mmu-17_19530* | 0.0 | 5.3 | *bta-22_20338* | 0.5 |
| *mmu-17_19530* | 0.0 | 2.0 | *bta-22_20340* | 3.0 |
| *mmu-17_19555* | 0.0 | 22.5 | *bta-22_20340* | 0.5 |
| *mmu-17_19562* | 0.0 | 14.0 | *bta-22_20343* | 1.0 |
| *mmu-17_19594* | 0.0 | 65.9 | *bta-22_20354* | 1.0 |
| *mmu-17_19641* | 0.0 | 48.1 | *bta-22_20356* | 1.0 |
| *mmu-17_19692* | 0.0 | 25.6 | *bta-22_20358* | 11.0 |
| *mmu-17_19703* | 1.7 | 68.9 | *bta-22_20438* | 0.5 |
| *mmu-17_19711* | 0.0 | 12.0 | *bta-22_20612* | 1.0 |
| *mmu-17_19795* | 0.0 | 9.3 | *bta-22_20670* | 2.5 |
| *mmu-17_19819* | 0.0 | 44.2 | *bta-22_20670* | 1.0 |
| *mmu-17_19939* | 0.0 | 8.0 | *bta-22_20912* | 2.0 |
| *mmu-17_19969* | 0.3 | 37.7 | *bta-22_20963* | 31.0 |
| *mmu-17_19973* | 0.0 | 38.0 | *bta-22_21004* | 1.5 |
| *mmu-17_20030* | 0.0 | 50.3 | *bta-22_21082* | 1.0 |
| *mmu-17_20034* | 0.0 | 11.3 | *bta-22_21087* | 25.9 |
| *mmu-17_20168* | 0.0 | 60.5 | *bta-22_21141* | 548.4 |
| *mmu-17_20168* | 0.0 | 3.3 | *bta-23_21249* | 0.5 |
| *mmu-17_20175* | 0.0 | 42.7 | *bta-23_21269* | 1.0 |
| *mmu-17_20313* | 466.3 | 19.3 | *bta-23_21303* | 1.0 |
| *mmu-17_20313* | 0.0 | 0.7 | *bta-23_21317* | 3.5 |
| *mmu-17_20332* | 0.0 | 5.3 | *bta-23_21647* | 2.0 |
| *mmu-18_20413* | 2.7 | 29.5 | *bta-23_21705* | 13.5 |
| *mmu-18_20471* | 0.0 | 13.7 | *bta-23_21705* | 2.0 |
| *mmu-18_20471* | 0.0 | 3.3 | *bta-23_22034* | 1.5 |
| *mmu-18_20484* | 0.0 | 4.7 | *bta-23_22081* | 5.5 |
| *mmu-18_20484* | 0.6 | 0.0 | *bta-23_22081* | 0.5 |
| *mmu-18_20512* | 0.0 | 5.3 | *bta-23_22137* | 1.0 |
| *mmu-18_20567* | 0.0 | 6.0 | *bta-23_22185* | 153.9 |
| *mmu-18_20571* | 0.0 | 15.5 | *bta-23_22193* | 8.5 |
| *mmu-18_20665* | 8.3 | 0.0 | *bta-23_22217* | 0.5 |
| *mmu-18_20692* | 5.6 | 49.9 | *bta-23_22447* | 0.5 |
| *mmu-18_20692* | 1.1 | 0.0 | *bta-24_22709* | 12.9 |
| *mmu-18_20722* | 0.0 | 42.7 | *bta-24_22762* | 4.5 |
| *mmu-18_20765* | 0.0 | 6.0 | *bta-24_22768* | 2.5 |
| *mmu-18_20979* | 0.0 | 4.7 | *bta-24_23039* | 133.3 |
| *mmu-18_21031* | 0.0 | 4.7 | *bta-24_23112* | 0.5 |
| *mmu-18_21203* | 0.0 | 28.7 | *bta-24_23237* | 1.5 |
| *mmu-18_21260* | 0.0 | 39.8 | *bta-24_23239* | 5.0 |
| *mmu-18_21271* | 0.0 | 4.7 | *bta-24_23264* | 1.5 |
| *mmu-18_21287* | 0.0 | 27.9 | *bta-24_23264* | 0.5 |
| *mmu-18_21309* | 0.0 | 5.3 | *bta-25_23395* | 1.0 |
| *mmu-18_21334* | 0.0 | 10.0 | *bta-25_23442* | 482.0 |
| *mmu-18_21361* | 0.0 | 44.2 | *bta-25_23442* | 0.5 |
| *mmu-18_21456* | 0.0 | 7.3 | *bta-25_23474* | 16.0 |
| *mmu-18_21458* | 0.0 | 31.0 | *bta-25_23474* | 3.5 |
| *mmu-18_21464* | 0.0 | 13.2 | *bta-25_23498* | 15.5 |
| *mmu-18_21692* | 0.0 | 5.3 | *bta-25_23569* | 5.0 |
| *mmu-18_21704* | 0.3 | 12.2 | *bta-25_23584* | 2.5 |
| *mmu-18_21754* | 0.0 | 9.3 | *bta-25_23601* | 6.0 |
| *mmu-19_21842* | 0.0 | 31.8 | *bta-25_23697* | 1.0 |
| *mmu-19_21892* | 4.9 | 2.7 | *bta-25_23784* | 8.5 |
| *mmu-19_21922* | 0.3 | 173.8 | *bta-25_23962* | 1.0 |
| *mmu-19_21944* | 0.0 | 5.3 | *bta-25_24004* | 4.0 |
| *mmu-19_21949* | 0.0 | 7.3 | *bta-25_24022* | 33.9 |
| *mmu-19_21998* | 0.0 | 6.0 | *bta-25_24037* | 1.0 |
| *mmu-19_22030* | 0.0 | 31.0 | *bta-25_24041* | 1.0 |
| *mmu-19_22255* | 0.9 | 2.0 | *bta-25_24045* | 2.0 |
| *mmu-19_22258* | 0.0 | 6.7 | *bta-25_24045* | 0.5 |
| *mmu-19_22297* | 0.0 | 34.1 | *bta-25_24121* | 1.0 |
| *mmu-19_22387* | 0.0 | 30.2 | *bta-25_24128* | 1.0 |
| *mmu-19_22398* | 0.0 | 42.7 | *bta-25_24128* | 0.5 |
| *mmu-19_22406* | 0.0 | 4.7 | *bta-25_24137* | 0.5 |
| *mmu-19_22514* | 0.0 | 54.6 | *bta-25_24138* | 12.0 |
| *mmu-19_22574* | 0.0 | 4.7 | *bta-25_24176* | 1.0 |
| *mmu-19_22636* | 0.0 | 28.7 | *bta-25_24176* | 1.0 |
| *mmu-19_22654* | 0.0 | 4.7 | *bta-25_24233* | 2.5 |
| *mmu-19_22660* | 0.0 | 8.7 | *bta-25_24332* | 14.5 |
| *mmu-19_22739* | 0.0 | 29.5 | *bta-25_24398* | 1.0 |
| *mmu-19_22742* | 0.0 | 32.6 | *bta-25_24462* | 3.0 |
| *mmu-19_22806* | 0.0 | 42.7 | *bta-25_24489* | 1.0 |
| *mmu-19_22806* | 0.0 | 2.0 | *bta-25_24592* | 1.0 |
| *mmu-19_22837* | 0.0 | 19.1 | *bta-25_24599* | 14.6 |
| *mmu-19_22953* | 0.0 | 20.9 | *bta-25_24620* | 1.0 |
| *mmu-19_22987* | 0.0 | 7.3 | *bta-25_24629* | 1.5 |
| *mmu-19_23098* | 0.0 | 29.5 | *bta-25_24638* | 1.0 |
| *mmu-19_23099* | 0.0 | 17.1 | *bta-26_24821* | 1.0 |
| *mmu-19_23099* | 0.0 | 3.3 | *bta-26_24833* | 1.0 |
| *mmu-19_23176* | 0.0 | 46.5 | *bta-26_24854* | 1.0 |
| *mmu-19_23244* | 0.0 | 43.4 | *bta-26_24925* | 558.1 |
| *mmu-19_23333* | 0.0 | 26.4 | *bta-26_24925* | 300.7 |
| *mmu-19_23442* | 0.0 | 86.1 | *bta-26_24993* | 2.5 |
| *mmu-19_23519* | 0.0 | 44.2 | *bta-26_25006* | 1.0 |
| *mmu-19_23571* | 0.0 | 12.6 | *bta-26_25020* | 0.5 |
| *mmu-19_23598* | 6.9 | 0.0 | *bta-26_25047* | 0.5 |
| *mmu-19_23607* | 0.0 | 4.7 | *bta-26_25272* | 3.5 |
| *mmu-2_23747* | 4.4 | 0.0 | *bta-26_25298* | 30.9 |
| *mmu-2_23747* | 0.7 | 0.0 | *bta-26_25359* | 16.5 |
| *mmu-2_23833* | 0.0 | 44.2 | *bta-26_25392* | 298.4 |
| *mmu-2_23876* | 492.2 | 3.4 | *bta-26_25392* | 1.5 |
| *mmu-2_23912* | 0.0 | 61.3 | *bta-26_25395* | 3.0 |
| *mmu-2_23932* | 0.0 | 52.0 | *bta-26_25414* | 5.0 |
| *mmu-2_23982* | 0.0 | 14.7 | *bta-26_25421* | 213.2 |
| *mmu-2_24008* | 0.0 | 20.9 | *bta-26_25421* | 0.5 |
| *mmu-2_24033* | 0.0 | 12.4 | *bta-26_25427* | 1.5 |
| *mmu-2_24066* | 0.0 | 7.0 | *bta-26_25436* | 14.0 |
| *mmu-2_24092* | 0.0 | 8.7 | *bta-27_25727* | 2.5 |
| *mmu-2_24241* | 0.0 | 40.3 | *bta-27_25756* | 1.0 |
| *mmu-2_24274* | 0.0 | 31.8 | *bta-27_25882* | 1.0 |
| *mmu-2_24371* | 22.3 | 63.3 | *bta-28_25946* | 2.0 |
| *mmu-2_24371* | 0.3 | 0.0 | *bta-28_25961* | 8.5 |
| *mmu-2_24597* | 0.0 | 20.6 | *bta-28_26039* | 1.0 |
| *mmu-2_24597* | 0.6 | 0.0 | *bta-28_26133* | 0.5 |
| *mmu-2_24638* | 448.2 | 726.8 | *bta-28_26220* | 1.0 |
| *mmu-2_24663* | 0.0 | 55.1 | *bta-28_26273* | 0.5 |
| *mmu-2_24871* | 0.0 | 7.3 | *bta-28_26492* | 101.6 |
| *mmu-2_24894* | 0.0 | 24.0 | *bta-28_26494* | 17.5 |
| *mmu-2_25023* | 0.0 | 5.3 | *bta-28_26494* | 4.5 |
| *mmu-2_25072* | 0.0 | 8.0 | *bta-29_26616* | 22.6 |
| *mmu-2_25096* | 0.0 | 28.7 | *bta-29_26616* | 2.5 |
| *mmu-2_25118* | 0.0 | 6.0 | *bta-29_26636* | 56.0 |
| *mmu-2_25129* | 0.0 | 12.0 | *bta-29_26636* | 1.5 |
| *mmu-2_25169* | 0.0 | 4.7 | *bta-29_26660* | 6.5 |
| *mmu-2_25204* | 0.0 | 31.7 | *bta-29_26761* | 1.0 |
| *mmu-2_25215* | 0.0 | 10.9 | *bta-29_26776* | 52.5 |
| *mmu-2_25236* | 0.0 | 8.5 | *bta-29_26776* | 0.5 |
| *mmu-2_25281* | 0.0 | 5.3 | *bta-29_26806* | 389.8 |
| *mmu-2_25288* | 0.0 | 30.2 | *bta-29_26897* | 1.5 |
| *mmu-2_25289* | 0.0 | 30.2 | *bta-29_26978* | 5.5 |
| *mmu-2_25304* | 0.3 | 27.9 | *bta-29_26978* | 3.0 |
| *mmu-2_25327* | 0.0 | 38.8 | *bta-29_27008* | 1.5 |
| *mmu-2_25440* | 0.0 | 24.8 | *bta-29_27022* | 0.5 |
| *mmu-2_25553* | 0.0 | 24.0 | *bta-29_27069* | 1.0 |
| *mmu-2_25634* | 0.0 | 17.8 | *bta-29_27085* | 1.0 |
| *mmu-2_25689* | 0.0 | 50.4 | *bta-29_27116* | 1.5 |
| *mmu-2_25689* | 0.0 | 2.7 | *bta-29_27123* | 1.0 |
| *mmu-2_25721* | 0.0 | 6.0 | *bta-29_27143* | 6.5 |
| *mmu-2_25721* | 0.3 | 0.0 | *bta-29_27215* | 1.0 |
| *mmu-2_25724* | 0.0 | 8.0 | *bta-29_27289* | 1.5 |
| *mmu-2_25787* | 0.0 | 5.3 | *bta-29_27432* | 8.5 |
| *mmu-2_25806* | 0.0 | 49.6 | *bta-29_27481* | 1.0 |
| *mmu-2_25865* | 0.0 | 38.0 | *bta-29_27506* | 2.0 |
| *mmu-2_25935* | 0.0 | 5.3 | *bta-29_27558* | 161.1 |
| *mmu-2_25980* | 0.0 | 28.7 | *bta-29_27558* | 3.0 |
| *mmu-2_26012* | 0.0 | 81.2 | *bta-29_27565* | 5.5 |
| *mmu-2_26041* | 0.0 | 11.6 | *bta-29_27568* | 1.5 |
| *mmu-2_26150* | 0.0 | 7.3 | *bta-29_27600* | 11.5 |
| *mmu-2_26175* | 0.0 | 31.8 | *bta-29_27600* | 2.0 |
| *mmu-2_26234* | 0.0 | 26.4 | *bta-3_27628* | 92.3 |
| *mmu-2_26356* | 0.0 | 17.8 | *bta-3_27628* | 2.0 |
| *mmu-2_26377* | 0.0 | 4.7 | *bta-3_27763* | 1.0 |
| *mmu-2_26600* | 0.0 | 6.0 | *bta-3_27838* | 4.5 |
| *mmu-2_26825* | 0.0 | 34.1 | *bta-3_28066* | 3.0 |
| *mmu-2_26838* | 0.0 | 5.3 | *bta-3_28099* | 64.4 |
| *mmu-2_26845* | 0.0 | 10.6 | *bta-3_28218* | 2.0 |
| *mmu-2_26900* | 0.0 | 65.9 | *bta-3_28373* | 1.5 |
| *mmu-2_26901* | 0.0 | 22.5 | *bta-3_28453* | 1.0 |
| *mmu-2_26913* | 0.0 | 22.5 | *bta-3_28477* | 1.5 |
| *mmu-2_26940* | 0.0 | 7.3 | *bta-3_28537* | 5.0 |
| *mmu-2_27050* | 0.0 | 18.6 | *bta-3_28571* | 2.0 |
| *mmu-2_27061* | 0.7 | 41.9 | *bta-3_28584* | 0.5 |
| *mmu-2_27065* | 0.0 | 10.1 | *bta-3_28615* | 4.0 |
| *mmu-2_27067* | 0.0 | 38.8 | *bta-3_28615* | 0.5 |
| *mmu-2_27075* | 0.0 | 4.7 | *bta-3_28697* | 5.0 |
| *mmu-2_27198* | 0.0 | 37.2 | *bta-3_28904* | 1.0 |
| *mmu-2_27209* | 0.0 | 42.7 | *bta-3_28904* | 1.0 |
| *mmu-2_27262* | 0.0 | 25.6 | *bta-3_28939* | 6.5 |
| *mmu-3_27397* | 0.0 | 7.3 | *bta-3_28973* | 1.0 |
| *mmu-3_27415* | 0.0 | 5.3 | *bta-3_29060* | 2.0 |
| *mmu-3_27477* | 0.0 | 39.1 | *bta-3_29216* | 0.5 |
| *mmu-3_27510* | 0.0 | 13.2 | *bta-3_29216* | 1.0 |
| *mmu-3_27709* | 0.0 | 38.0 | *bta-3_29333* | 122.9 |
| *mmu-3_27709* | 0.0 | 5.3 | *bta-3_29372* | 6.0 |
| *mmu-3_27719* | 0.0 | 51.2 | *bta-3_29445* | 2.0 |
| *mmu-3_27773* | 0.0 | 53.5 | *bta-3_29604* | 2.0 |
| *mmu-3_27827* | 0.0 | 9.3 | *bta-3_29611* | 1.0 |
| *mmu-3_28020* | 0.0 | 32.6 | *bta-4_29887* | 4.0 |
| *mmu-3_28020* | 0.4 | 0.0 | *bta-4_29891* | 0.5 |
| *mmu-3_28162* | 0.0 | 20.9 | *bta-4_29891* | 0.5 |
| *mmu-3_28288* | 0.0 | 64.4 | *bta-4_29931* | 61.0 |
| *mmu-3_28325* | 1165.1 | 2053.6 | *bta-4_29949* | 4.0 |
| *mmu-3_28427* | 0.3 | 10.6 | *bta-4_29964* | 1.0 |
| *mmu-3_28430* | 0.0 | 22.5 | *bta-4_29964* | 1.0 |
| *mmu-3_28531* | 0.0 | 4.7 | *bta-4_30095* | 2.5 |
| *mmu-3_28634* | 0.0 | 53.5 | *bta-4_30189* | 1.5 |
| *mmu-3_28725* | 0.0 | 55.1 | *bta-4_30272* | 10.5 |
| *mmu-3_28807* | 0.0 | 17.8 | *bta-4_30276* | 3.5 |
| *mmu-3_28911* | 0.0 | 21.7 | *bta-4_30568* | 1.0 |
| *mmu-3_28942* | 0.0 | 4.7 | *bta-4_30892* | 1.5 |
| *mmu-3_28943* | 0.0 | 36.5 | *bta-4_30919* | 1.0 |
| *mmu-3_28972* | 0.0 | 6.7 | *bta-4_30967* | 9.0 |
| *mmu-3_29022* | 0.0 | 5.3 | *bta-5_31204* | 0.5 |
| *mmu-3_29129* | 9.8 | 1.3 | *bta-5_31373* | 6.5 |
| *mmu-3_29131* | 7.1 | 12.6 | *bta-5_31417* | 14.0 |
| *mmu-3_29135* | 0.0 | 63.6 | *bta-5_31457* | 40.0 |
| *mmu-3_29221* | 0.0 | 6.0 | *bta-5_31457* | 2.5 |
| *mmu-3_29345* | 0.0 | 26.4 | *bta-5_31639* | 0.5 |
| *mmu-3_29393* | 0.0 | 6.0 | *bta-5_31760* | 0.5 |
| *mmu-3_29393* | 0.0 | 1.3 | *bta-5_31809* | 4.0 |
| *mmu-3_29492* | 0.0 | 49.2 | *bta-5_31809* | 0.5 |
| *mmu-3_29599* | 19.9 | 2.7 | *bta-5_31859* | 11.0 |
| *mmu-3_29716* | 0.0 | 49.6 | *bta-5_31859* | 2.5 |
| *mmu-3_29716* | 0.0 | 0.7 | *bta-5_31916* | 1.0 |
| *mmu-3_29721* | 0.0 | 4.7 | *bta-5_31934* | 4.5 |
| *mmu-3_29728* | 0.0 | 27.9 | *bta-5_31998* | 1.5 |
| *mmu-3_29746* | 1.2 | 5.3 | *bta-5_31998* | 0.5 |
| *mmu-3_29770* | 0.0 | 21.8 | *bta-5_32032* | 0.5 |
| *mmu-4_29835* | 0.0 | 25.9 | *bta-5_32082* | 2.0 |
| *mmu-4_29835* | 0.0 | 5.3 | *bta-5_32096* | 8.0 |
| *mmu-4_29850* | 0.0 | 9.8 | *bta-5_32096* | 3.0 |
| *mmu-4_30034* | 0.4 | 8.0 | *bta-5_32132* | 7.0 |
| *mmu-4_30229* | 0.3 | 8.0 | *bta-5_32308* | 1.0 |
| *mmu-4_30412* | 0.0 | 4.7 | *bta-5_32444* | 1.0 |
| *mmu-4_30441* | 0.0 | 6.7 | *bta-5_32567* | 7441.5 |
| *mmu-4_30457* | 0.0 | 5.3 | *bta-5_32590* | 1.0 |
| *mmu-4_30653* | 34.2 | 2.7 | *bta-5_32635* | 2.0 |
| *mmu-4_30653* | 0.6 | 4.0 | *bta-5_32642* | 0.5 |
| *mmu-4_30697* | 0.3 | 58.9 | *bta-5_32781* | 11.5 |
| *mmu-4_30727* | 0.0 | 19.4 | *bta-5_33180* | 3.0 |
| *mmu-4_30795* | 0.0 | 8.0 | *bta-6_33549* | 23.0 |
| *mmu-4_30836* | 0.0 | 4.7 | *bta-6_33629* | 3.0 |
| *mmu-4_30933* | 0.0 | 56.6 | *bta-6_33846* | 1.0 |
| *mmu-4_30968* | 0.0 | 5.3 | *bta-6_33860* | 390.1 |
| *mmu-4_30974* | 0.0 | 20.2 | *bta-6_33860* | 4.5 |
| *mmu-4_31072* | 0.0 | 33.7 | *bta-6_33876* | 2.5 |
| *mmu-4_31117* | 0.0 | 4.7 | *bta-6_33876* | 0.5 |
| *mmu-4_31117* | 0.0 | 2.0 | *bta-6_33889* | 0.5 |
| *mmu-4_31140* | 0.0 | 6.7 | *bta-6_34001* | 2.0 |
| *mmu-4_31180* | 0.0 | 46.5 | *bta-6_34001* | 0.5 |
| *mmu-4_31286* | 1.0 | 36.5 | *bta-6_34009* | 1.0 |
| *mmu-4_31286* | 0.3 | 0.0 | *bta-6_34049* | 1.0 |
| *mmu-4_31314* | 0.0 | 4.7 | *bta-6_34192* | 1.0 |
| *mmu-4_31425* | 0.0 | 48.1 | *bta-6_34203* | 38.6 |
| *mmu-4_31494* | 0.0 | 5.3 | *bta-6_34506* | 44.1 |
| *mmu-4_31494* | 0.0 | 5.3 | *bta-6_34506* | 0.5 |
| *mmu-4_31504* | 0.0 | 38.0 | *bta-6_34515* | 1.0 |
| *mmu-4_31859* | 100.3 | 245.2 | *bta-6_34539* | 9.0 |
| *mmu-4_31863* | 0.0 | 233.9 | *bta-6_34539* | 5.5 |
| *mmu-4_31897* | 0.0 | 6.7 | *bta-6_34563* | 3.5 |
| *mmu-4_31905* | 0.0 | 23.3 | *bta-6_34600* | 1.0 |
| *mmu-4_31929* | 2.5 | 0.0 | *bta-7_34616* | 0.5 |
| *mmu-4_32017* | 0.0 | 10.9 | *bta-7_34616* | 0.5 |
| *mmu-4_32028* | 0.0 | 14.7 | *bta-7_34681* | 1.0 |
| *mmu-4_32062* | 0.0 | 35.7 | *bta-7_34740* | 2.0 |
| *mmu-4_32298* | 3.2 | 13.3 | *bta-7_34740* | 1.0 |
| *mmu-4_32320* | 0.0 | 58.9 | *bta-7_34743* | 0.5 |
| *mmu-4_32327* | 0.6 | 22.6 | *bta-7_34884* | 35.1 |
| *mmu-4_32327* | 0.0 | 1.3 | *bta-7_34931* | 15.5 |
| *mmu-4_32473* | 0.0 | 9.3 | *bta-7_35033* | 3.0 |
| *mmu-4_32570* | 0.0 | 5.3 | *bta-7_35090* | 11.5 |
| *mmu-4_32576* | 0.0 | 31.8 | *bta-7_35338* | 3.0 |
| *mmu-4_32636* | 0.0 | 4.7 | *bta-7_35358* | 1.0 |
| *mmu-4_32686* | 0.4 | 34.9 | *bta-7_35384* | 564.3 |
| *mmu-4_32689* | 153.7 | 49.2 | *bta-7_35546* | 5.0 |
| *mmu-5_32748* | 0.0 | 20.2 | *bta-7_35597* | 7.5 |
| *mmu-5_32796* | 0.0 | 27.1 | *bta-7_35651* | 1.5 |
| *mmu-5_32905* | 0.0 | 41.1 | *bta-7_35679* | 1.5 |
| *mmu-5_33075* | 0.0 | 7.3 | *bta-7_35703* | 2.0 |
| *mmu-5_33265* | 0.0 | 21.7 | *bta-7_35746* | 1.0 |
| *mmu-5_33329* | 0.0 | 49.6 | *bta-7_35806* | 2.0 |
| *mmu-5_33869* | 1.5 | 0.0 | *bta-7_35869* | 4.0 |
| *mmu-5_33956* | 0.0 | 12.2 | *bta-7_35869* | 1.0 |
| *mmu-5_33983* | 0.0 | 4.7 | *bta-7_35928* | 1.5 |
| *mmu-5_33988* | 0.4 | 56.4 | *bta-7_35950* | 1.0 |
| *mmu-5_33990* | 0.7 | 7.3 | *bta-7_36100* | 1.0 |
| *mmu-5_33991* | 0.3 | 30.1 | *bta-7_36193* | 1.0 |
| *mmu-5_34166* | 1.1 | 73.2 | *bta-7_36333* | 23.5 |
| *mmu-5_34205* | 0.0 | 28.7 | *bta-7_36333* | 0.5 |
| *mmu-5_34223* | 0.0 | 17.8 | *bta-7_36346* | 31.0 |
| *mmu-5_34227* | 0.0 | 65.9 | *bta-7_36390* | 7.0 |
| *mmu-5_34227* | 0.0 | 2.0 | *bta-7_36478* | 4.5 |
| *mmu-5_34272* | 0.0 | 4.7 | *bta-7_36505* | 1.5 |
| *mmu-5_34286* | 0.0 | 39.6 | *bta-8_36580* | 1.0 |
| *mmu-5_34313* | 0.0 | 32.6 | *bta-8_36658* | 1.0 |
| *mmu-5_34337* | 0.0 | 69.8 | *bta-8_36917* | 0.5 |
| *mmu-5_34352* | 0.0 | 4.7 | *bta-8_36971* | 1.0 |
| *mmu-5_34361* | 0.0 | 13.2 | *bta-8_37049* | 5.5 |
| *mmu-5_34456* | 0.0 | 17.8 | *bta-8_37065* | 5.0 |
| *mmu-5_34479* | 0.0 | 5.3 | *bta-8_37088* | 5.5 |
| *mmu-5_34483* | 0.0 | 4.7 | *bta-8_37088* | 2.5 |
| *mmu-5_34514* | 0.0 | 14.0 | *bta-8_37135* | 1.0 |
| *mmu-5_34519* | 0.7 | 130.2 | *bta-8_37440* | 1.0 |
| *mmu-5_34769* | 0.0 | 5.3 | *bta-8_37440* | 0.5 |
| *mmu-5_34802* | 20.1 | 35.8 | *bta-8_37539* | 245.6 |
| *mmu-5_34802* | 2.0 | 5.3 | *bta-8_37539* | 0.5 |
| *mmu-5_34809* | 0.0 | 8.0 | *bta-8_37739* | 109.8 |
| *mmu-5_34813* | 3.3 | 0.0 | *bta-8_37752* | 1.5 |
| *mmu-5_34887* | 0.0 | 13.2 | *bta-8_37752* | 1.5 |
| *mmu-5_34938* | 0.0 | 10.9 | *bta-8_37792* | 14.0 |
| *mmu-5_35028* | 0.0 | 284.3 | *bta-8_37934* | 1.0 |
| *mmu-5_35236* | 0.0 | 78.3 | *bta-8_38001* | 1.5 |
| *mmu-5_35246* | 0.0 | 20.2 | *bta-9_38080* | 33.6 |
| *mmu-5_35403* | 0.0 | 48.9 | *bta-9_38080* | 3.5 |
| *mmu-5_35425* | 0.0 | 5.3 | *bta-9_38092* | 1.0 |
| *mmu-5_35425* | 0.0 | 2.0 | *bta-9_38390* | 10.5 |
| *mmu-5_35519* | 0.0 | 6.0 | *bta-9_38460* | 1.0 |
| *mmu-5_35559* | 0.0 | 6.7 | *bta-9_38758* | 6.0 |
| *mmu-5_35586* | 0.0 | 38.8 | *bta-9_38867* | 13.0 |
| *mmu-5_35594* | 0.0 | 4.7 | *bta-9_39096* | 1.0 |
| *mmu-5_35611* | 0.0 | 40.4 | *bta-9_39097* | 1.5 |
| *mmu-5_35660* | 0.0 | 4.7 | *bta-9_39116* | 1.0 |
| *mmu-5_35694* | 0.0 | 4.7 | *bta-GJ060136.1_40809* | 0.5 |
| *mmu-5_35697* | 0.0 | 42.0 | *bta-X_39354* | 2.5 |
| *mmu-5_35787* | 0.0 | 58.2 | *bta-X_39460* | 5.0 |
| *mmu-5_35887* | 0.0 | 4.7 | *bta-X_39460* | 0.5 |
| *mmu-5_35908* | 0.0 | 21.7 | *bta-X_39461* | 1.0 |
| *mmu-5_35935* | 0.0 | 39.6 | *bta-X_39470* | 62.5 |
| *mmu-5_35996* | 0.0 | 6.7 | *bta-X_39470* | 2.5 |
| *mmu-5_36020* | 0.0 | 25.6 | *bta-X_39471* | 4.0 |
| *mmu-5_36072* | 0.0 | 31.0 | *bta-X_39613* | 7.5 |
| *mmu-5_36093* | 0.0 | 5.3 | *bta-X_39615* | 0.5 |
| *mmu-5_36146* | 3.5 | 5.3 | *bta-X_39888* | 1.0 |
| *mmu-5_36189* | 0.0 | 22.5 | *bta-X_40081* | 1.5 |
| *mmu-6_36293* | 0.0 | 28.7 | *bta-X_40085* | 1.0 |
| *mmu-6_36336* | 0.0 | 49.6 | *bta-X_40144* | 1.5 |
| *mmu-6_36421* | 0.0 | 36.5 | *bta-X_40355* | 9.5 |
| *mmu-6_36423* | 2.5 | 41.1 | *bta-X_40394* | 5.0 |
| *mmu-6_36669* | 0.0 | 19.4 | *bta-X_40394* | 2.0 |
| *mmu-6_36691* | 0.0 | 55.8 | *bta-X_40395* | 8.5 |
| *mmu-6_36691* | 0.0 | 2.7 | *bta-X_40395* | 0.5 |
| *mmu-6_36737* | 0.0 | 41.9 | *bta-X_40470* | 1.0 |
| *mmu-6_36737* | 0.0 | 4.0 | *bta-X_40548* | 1.5 |
| *mmu-6_36847* | 0.0 | 27.9 | *bta-X_40615* | 0.5 |
| *mmu-6_36947* | 0.0 | 31.3 | *bta-X_40736* | 1.0 |
| *mmu-6_36950* | 0.4 | 59.7 | *bta-X_40736* | 0.5 |
| *mmu-6_37008* | 0.0 | 8.0 | *bta-X_40741* | 3.0 |
| *mmu-6_37012* | 0.0 | 44.2 |  |  |
| *mmu-6_37012* | 0.0 | 2.7 |  |  |
| *mmu-6_37065* | 0.0 | 6.0 |  |  |
| *mmu-6_37176* | 0.0 | 4.7 |  |  |
| *mmu-6_37491* | 0.0 | 5.3 |  |  |
| *mmu-6_37530* | 0.4 | 36.3 |  |  |
| *mmu-6_37608* | 0.0 | 41.1 |  |  |
| *mmu-6_37623* | 0.0 | 4.7 |  |  |
| *mmu-6_37779* | 3.1 | 5.3 |  |  |
| *mmu-6_37787* | 0.0 | 17.8 |  |  |
| *mmu-6_37864* | 0.0 | 60.5 |  |  |
| *mmu-6_37879* | 0.0 | 30.2 |  |  |
| *mmu-6_38019* | 0.0 | 90.0 |  |  |
| *mmu-6_38282* | 1.4 | 14.4 |  |  |
| *mmu-6_38365* | 0.0 | 8.0 |  |  |
| *mmu-6_38481* | 0.0 | 58.2 |  |  |
| *mmu-6_38481* | 0.0 | 1.3 |  |  |
| *mmu-7_38549* | 3.4 | 0.0 |  |  |
| *mmu-7_38551* | 16.9 | 224.8 |  |  |
| *mmu-7_38583* | 0.0 | 5.3 |  |  |
| *mmu-7_38652* | 0.0 | 10.1 |  |  |
| *mmu-7_38713* | 0.0 | 14.0 |  |  |
| *mmu-7_38823* | 0.0 | 14.0 |  |  |
| *mmu-7_38846* | 0.0 | 31.0 |  |  |
| *mmu-7_38864* | 0.0 | 14.0 |  |  |
| *mmu-7_38937* | 0.0 | 4.7 |  |  |
| *mmu-7_39016* | 0.0 | 6.0 |  |  |
| *mmu-7_39061* | 0.0 | 4.7 |  |  |
| *mmu-7_39125* | 0.0 | 19.4 |  |  |
| *mmu-7_39180* | 0.0 | 8.7 |  |  |
| *mmu-7_39206* | 0.0 | 10.0 |  |  |
| *mmu-7_39208* | 0.0 | 42.0 |  |  |
| *mmu-7_39244* | 0.0 | 4.7 |  |  |
| *mmu-7_39244* | 0.0 | 4.0 |  |  |
| *mmu-7_39265* | 0.0 | 47.3 |  |  |
| *mmu-7_39265* | 0.0 | 1.3 |  |  |
| *mmu-7_39298* | 5.2 | 33.1 |  |  |
| *mmu-7_39423* | 0.0 | 31.0 |  |  |
| *mmu-7_39433* | 0.0 | 30.2 |  |  |
| *mmu-7_39433* | 0.7 | 0.0 |  |  |
| *mmu-7_39451* | 0.0 | 4.7 |  |  |
| *mmu-7_39520* | 0.0 | 8.7 |  |  |
| *mmu-7_39605* | 0.0 | 12.4 |  |  |
| *mmu-7_39646* | 0.0 | 14.6 |  |  |
| *mmu-7_39665* | 0.0 | 46.5 |  |  |
| *mmu-7_39685* | 0.0 | 8.7 |  |  |
| *mmu-7_39687* | 0.0 | 28.7 |  |  |
| *mmu-7_39719* | 0.0 | 52.0 |  |  |
| *mmu-7_39788* | 0.0 | 8.5 |  |  |
| *mmu-7_39813* | 0.0 | 6.0 |  |  |
| *mmu-7_39856* | 0.4 | 42.7 |  |  |
| *mmu-7_39874* | 0.0 | 5.3 |  |  |
| *mmu-7_39955* | 0.0 | 54.0 |  |  |
| *mmu-7_39973* | 0.0 | 4.7 |  |  |
| *mmu-7_40036* | 0.0 | 31.8 |  |  |
| *mmu-7_40093* | 0.0 | 6.7 |  |  |
| *mmu-7_40105* | 0.0 | 7.3 |  |  |
| *mmu-7_40132* | 0.0 | 8.5 |  |  |
| *mmu-7_40185* | 0.0 | 6.7 |  |  |
| *mmu-7_40211* | 0.0 | 5.3 |  |  |
| *mmu-7_40276* | 0.0 | 31.0 |  |  |
| *mmu-7_40291* | 0.0 | 4.7 |  |  |
| *mmu-7_40334* | 0.0 | 14.0 |  |  |
| *mmu-7_40560* | 0.0 | 4.7 |  |  |
| *mmu-7_40565* | 0.0 | 22.5 |  |  |
| *mmu-7_40580* | 11.9 | 126.4 |  |  |
| *mmu-7_40594* | 0.0 | 18.6 |  |  |
| *mmu-7_40594* | 0.0 | 0.7 |  |  |
| *mmu-7_40604* | 0.0 | 76.8 |  |  |
| *mmu-7_40608* | 0.0 | 4.7 |  |  |
| *mmu-7_40625* | 0.0 | 6.7 |  |  |
| *mmu-7_40783* | 0.0 | 4.7 |  |  |
| *mmu-7_40846* | 0.0 | 4.7 |  |  |
| *mmu-7_40895* | 0.0 | 36.5 |  |  |
| *mmu-7_40907* | 0.0 | 34.1 |  |  |
| *mmu-7_41020* | 0.0 | 27.1 |  |  |
| *mmu-7_41166* | 0.7 | 437.5 |  |  |
| *mmu-8_41233* | 0.0 | 46.5 |  |  |
| *mmu-8_41293* | 0.0 | 37.2 |  |  |
| *mmu-8_41356* | 0.0 | 25.6 |  |  |
| *mmu-8_41411* | 0.0 | 13.2 |  |  |
| *mmu-8_41481* | 0.0 | 39.6 |  |  |
| *mmu-8_41490* | 0.0 | 68.2 |  |  |
| *mmu-8_41544* | 0.0 | 18.6 |  |  |
| *mmu-8_41616* | 0.0 | 5.3 |  |  |
| *mmu-8_41659* | 0.0 | 7.3 |  |  |
| *mmu-8_41675* | 0.0 | 76.0 |  |  |
| *mmu-8_41713* | 0.0 | 36.5 |  |  |
| *mmu-8_41713* | 0.6 | 12.0 |  |  |
| *mmu-8_41770* | 0.0 | 24.8 |  |  |
| *mmu-8_41782* | 0.0 | 4.0 |  |  |
| *mmu-8_41795* | 0.0 | 4.7 |  |  |
| *mmu-8_41840* | 1.1 | 24.0 |  |  |
| *mmu-8_42009* | 0.0 | 4.7 |  |  |
| *mmu-8_42204* | 0.0 | 68.1 |  |  |
| *mmu-8_42216* | 0.0 | 48.9 |  |  |
| *mmu-8_42251* | 0.7 | 25.2 |  |  |
| *mmu-8_42297* | 0.0 | 6.0 |  |  |
| *mmu-8_42327* | 0.0 | 4.7 |  |  |
| *mmu-8_42456* | 0.0 | 5.3 |  |  |
| *mmu-8_42458* | 0.0 | 24.2 |  |  |
| *mmu-8_42461* | 0.0 | 26.4 |  |  |
| *mmu-8_42491* | 0.0 | 46.5 |  |  |
| *mmu-8_42592* | 0.0 | 34.1 |  |  |
| *mmu-8_42595* | 0.3 | 4.7 |  |  |
| *mmu-8_42633* | 0.0 | 109.1 |  |  |
| *mmu-8_42741* | 0.0 | 297.6 |  |  |
| *mmu-8_42892* | 0.9 | 47.9 |  |  |
| *mmu-8_42970* | 0.0 | 5.3 |  |  |
| *mmu-8_42982* | 0.0 | 8.5 |  |  |
| *mmu-8_43005* | 2.4 | 1142.3 |  |  |
| *mmu-8_43054* | 0.0 | 26.4 |  |  |
| *mmu-8_43231* | 0.0 | 84.4 |  |  |
| *mmu-8_43273* | 0.0 | 6.0 |  |  |
| *mmu-8_43297* | 0.0 | 53.5 |  |  |
| *mmu-8_43344* | 0.0 | 16.3 |  |  |
| *mmu-8_43383* | 0.0 | 23.3 |  |  |
| *mmu-8_43494* | 0.0 | 10.6 |  |  |
| *mmu-8_43528* | 0.0 | 37.2 |  |  |
| *mmu-9_43659* | 0.0 | 55.1 |  |  |
| *mmu-9_43663* | 12.6 | 0.0 |  |  |
| *mmu-9_43663* | 4.1 | 5.3 |  |  |
| *mmu-9_43664* | 0.0 | 35.9 |  |  |
| *mmu-9_43691* | 0.0 | 4.7 |  |  |
| *mmu-9_43698* | 0.0 | 38.0 |  |  |
| *mmu-9_43739* | 0.0 | 6.0 |  |  |
| *mmu-9_43883* | 0.0 | 8.0 |  |  |
| *mmu-9_43934* | 0.0 | 62.7 |  |  |
| *mmu-9_43952* | 0.0 | 34.9 |  |  |
| *mmu-9_43977* | 0.0 | 11.3 |  |  |
| *mmu-9_44027* | 1.1 | 4.0 |  |  |
| *mmu-9_44027* | 4.8 | 2.0 |  |  |
| *mmu-9_44084* | 0.0 | 21.3 |  |  |
| *mmu-9_44168* | 0.0 | 27.8 |  |  |
| *mmu-9_44441* | 0.0 | 10.6 |  |  |
| *mmu-9_44746* | 0.0 | 27.1 |  |  |
| *mmu-9_44754* | 0.0 | 99.8 |  |  |
| *mmu-9_44945* | 0.0 | 7.3 |  |  |
| *mmu-9_45002* | 0.0 | 30.2 |  |  |
| *mmu-9_45012* | 0.0 | 13.2 |  |  |
| *mmu-9_45155* | 0.0 | 22.5 |  |  |
| *mmu-9_45170* | 0.0 | 4.7 |  |  |
| *mmu-9_45184* | 1.2 | 1.4 |  |  |
| *mmu-9_45208* | 0.0 | 4.7 |  |  |
| *mmu-9_45368* | 0.0 | 6.0 |  |  |
| *mmu-9_45415* | 0.0 | 50.4 |  |  |
| *mmu-9_45417* | 0.0 | 10.1 |  |  |
| *mmu-9_45434* | 0.0 | 19.3 |  |  |
| *mmu-9_45439* | 0.0 | 17.1 |  |  |
| *mmu-9_45526* | 0.3 | 4.7 |  |  |
| *mmu-9_45685* | 0.0 | 24.0 |  |  |
| *mmu-9_45698* | 0.0 | 6.7 |  |  |
| *mmu-9_45878* | 0.6 | 69.2 |  |  |
| *mmu-9_45921* | 0.0 | 6.7 |  |  |
| *mmu-9_45985* | 0.0 | 6.0 |  |  |
| *mmu-9_45985* | 0.3 | 0.0 |  |  |
| *mmu-9_46012* | 0.0 | 4.7 |  |  |
| *mmu-X_46486* | 0.0 | 6.0 |  |  |
| *mmu-X_46486* | 0.0 | 2.0 |  |  |
| *mmu-X_46505* | 0.0 | 25.6 |  |  |
| *mmu-X_46740* | 0.4 | 251.4 |  |  |
| *mmu-X_46836* | 0.0 | 40.7 |  |  |
| *mmu-X_46860* | 0.0 | 4.7 |  |  |
| *mmu-X_47070* | 0.0 | 7.3 |  |  |
| *mmu-X_47075* | 0.0 | 7.8 |  |  |
| *mmu-X_47075* | 0.0 | 0.7 |  |  |
| *mmu-X_47084* | 0.3 | 172.2 |  |  |
| *mmu-X_47084* | 0.0 | 1.3 |  |  |
| *mmu-X_47093* | 0.0 | 13.2 |  |  |
| *mmu-X_47104* | 0.0 | 5.3 |  |  |
| *mmu-X_47230* | 0.0 | 4.7 |  |  |
| *mmu-X_47234* | 0.0 | 55.7 |  |  |
| *mmu-X_47418* | 0.0 | 5.3 |  |  |
| *mmu-X_47508* | 0.0 | 25.6 |  |  |
| *mmu-X_47768* | 0.0 | 5.3 |  |  |
