# Supplementary material for: Characterisation and Comparison of Lactating Mouse and Bovine Mammary Gland miRNomes
Source: PLoS One. 2014 Mar 21;9(3):e91938. doi: 10.1371/journal.pone.0091938 (PMC3962357; doi:10.1371/journal.pone.0091938)
Supplement: Table S3 — miRNA sharing identical seeds reaching cumulative 1,000 RPM threshold in lactating mouse and bovine mammary miRNomes. (DOCX) [file pone.0091938.s006.docx]

**Table S3. miRNA sharing identical seeds reaching cumulative 1,000 RPM threshold in lactating mouse and bovine mammary miRNomes**.

|  | **Mouse miRNome** | |  | **Bovine miRNome** | |  |
| --- | --- | --- | --- | --- | --- | --- |
| **Seed** | **RPM / seed** | **miRNA** | **RPM / miRNA** | **RPM / seed** | **miRNA** | **RPM / miRNA** |
| aacact | 190,123 | *mmu-miR-141-3p* | 48,559 | 33,276 | *bta-miR-141-3p* | 19,771 |
|  |  | *mmu-miR-200a-3p* | 141,563 |  | *bta-miR-200a-3p* | 13,506 |
| gaggta | 177,906 | *mmu-let-7a-1-5p* | 26,577 | 173,912 | *bta-13_7394_3p* | 48 |
|  |  | *mmu-let-7b-5p* | 13,212 |  | *bta-26_25395_5p* | 0.3 |
|  |  | *mmu-let-7c-5p* | 29,146 |  | *bta-let-7a-5p* | 67,530 |
|  |  | *mmu-let-7d-5p* | 10,365 |  | *bta-let-7b-5p* | 37,693 |
|  |  | *mmu-let-7e-5p* | 1,325 |  | *bta-let-7c-5p* | 9,705 |
|  |  | *mmu-let-7f-5p* | 28,912 |  | *bta-let-7d-5p* | 3,179 |
|  |  | *mmu-let-7g-5p* | 37,376 |  | *bta-let-7e-5p* | 1,858 |
|  |  | *mmu-let-7i-5p* | 29,906 |  | *bta-let-7f-5p* | 20,838 |
|  |  | *mmu-let-7k-5p* | 2 |  | *bta-let-7g-5p* | 21,149 |
|  |  | *mmu-miR-98-5p* | 859 |  | *bta-let-7i-5p* | 10,853 |
|  |  |  |  |  | *bta-miR-6119-5p* | 47 |
|  |  |  |  |  | *bta-miR-98-5p* | 1,012 |
| gagaac | 138,613 | *mmu-7_38551-3p* | 4 | 1,541 | *bta-miR-146a-5p* | 908 |
|  |  | *mmu-miR-146a-5p* | 1,543 |  | *bta-miR-146b-5p* | 633 |
|  |  | *mmu-miR-146b-5p* | 137,065 |  |  |  |
| cagtgc | 137,344 | *mmu-miR-148a-3p* | 128,383 | 77,417 | *bta-5_31639_3p* | 0.1 |
|  |  | *mmu-miR-148b-3p* | 2,215 |  | *bta-miR-148a-3p* | 74,417 |
|  |  | *mmu-miR-152-3p* | 6,745 |  | *bta-miR-148b-3p* | 1,089 |
|  |  | *mmu-miR-1960-5p* | 0.3 |  | *bta-miR-152-3p* | 1,912 |
| acattc | 97,928 | *mmu-miR-181a-5p* | 81,310 | 7,937 | *bta-miR-181a-5p* | 6,637 |
|  |  | *mmu-miR-181b-5p* | 15,411 |  | *bta-miR-181b-5p* | 1,222 |
|  |  | *mmu-miR-181c-5p* | 893 |  | *bta-miR-181c-5p* | 49 |
|  |  | *mmu-miR-181d-5p* | 313 |  | *bta-miR-181d-5p* | 30 |
| gagatg | 82,971 | *mmu-13_11638-5p* | 1 | 55,530 | *bta-1_650_5p* | 1 |
|  |  | *mmu-miR-143-3p* | 82,970 |  | *bta-miR-143-3p* | 55,530 |
| aatact | 64,996 | *mmu-miR-200b-3p* | 39,222 | 41,101 | *bta-miR-200b-3p* | 12,735 |
|  |  | *mmu-miR-200c-3p* | 17,478 |  | *bta-miR-200c-3p* | 27,496 |
|  |  | *mmu-miR-429-3p* | 8,296 |  | *bta-miR-429-3p* | 870 |
| agcacc | 59,769 | *mmu-miR-29a-3p* | 46,294 | 9,165 | *bta-miR-29b-3p* | 6,756 |
|  |  | *mmu-miR-29b-3p* | 12,440 |  | *bta-miR-29c-3p* | 2,399 |
|  |  | *mmu-miR-29c-3p* | 1,035 |  | *bta-miR-29d-3p* | 10 |
| tcaagt | 49,404 | *mmu-miR-26b-5p* | 11,278 | 92,119 | *bta-19_15181_5p* | 1 |
|  |  | *mmu-miR-26a-5p* | 38,126 |  | *bta-miR-26a-5p* | 69,784 |
|  |  |  |  |  | *bta-miR-26b-5p* | 22,334 |
| gtaaac | 44,151 | *mmu-miR-30a-5p* | 27,479 | 20,552 | *bta-miR-30a-5p* | 14,309 |
|  |  | *mmu-miR-30b-5p* | 516 |  | *bta-miR-30b-5p* | 107 |
|  |  | *mmu-miR-30c-5p* | 772 |  | *bta-miR-30c-5p* | 51 |
|  |  | *mmu-miR-30d-5p* | 7,080 |  | *bta-miR-30d-5p* | 4,062 |
|  |  | *mmu-miR-30e-5p* | 8,303 |  | *bta-miR-30e-5p* | 2,011 |
|  |  | *mmu-miR-384-5p* | 1 |  | *bta-miR-30f-5p* | 13 |
| gcagca | 38,639 | *mmu-miR-103-3p* | 31,549 | 12,298 | *bta-miR-103-3p* | 10,794 |
|  |  | *mmu-miR-107-3p* | 7,090 |  | *bta-miR-107-3p* | 1,504 |
| agcagc | 37,862 | *mmu-miR-15a-5p* | 6,385 | 53,534 | *bta-miR-15a-5p* | 6,284 |
|  |  | *mmu-miR-15b-5p* | 2,135 |  | *bta-miR-15b-5p* | 1,259 |
|  |  | *mmu-miR-16-5p* | 18,887 |  | *bta-miR-16a-5p* | 26,252 |
|  |  | *mmu-miR-195a-5p* | 1,989 |  | *bta-miR-16b-5p* | 5,945 |
|  |  | *mmu-miR-322-5p* | 2,700 |  | *bta-miR-195-5p* | 6,953 |
|  |  | *mmu-miR-497-5p* | 5,616 |  | *bta-miR-424-5p* | 1,503 |
|  |  | *mmu-miR-503-5p* | 151 |  | *bta-miR-497-5p* | 5,321 |
|  |  |  |  |  | *bta-miR-503-5p* | 19 |
| agctgc | 33,760 | *mmu-miR-22-3p* | 33,760 | 22,646 | *bta-miR-22-3p* | 22,646 |
| cgtacc | 26,967 | *mmu-miR-126a-3p* | 26,967 |  |  |  |
| tcacag | 26,232 | *mmu-9_44027-5p* | 0.3 | 23,861 | *bta-miR-27a-3p* | 7,204 |
|  |  | *mmu-miR-27a-3p* | 10,591 |  | *bta-miR-27b-3p* | 16,657 |
|  |  | *mmu-miR-27b-3p* | 15,639 |  |  |  |
|  |  | *mmu-miR-673-5p* | 2 |  |  |  |
|  |  | *mmu-miR-6985-3p* | 0.1 |  |  |  |
| ctggac | 25,774 | *mmu-miR-378a-3p* | 18,674 |  |  |  |
|  |  | *mmu-miR-378c-5p* | 7,100 |  |  |  |
| aaagtg | 25,397 | *mmu-miR-106a-5p* | 21 | 23,398 | *bta-6_34203_5p* | 4 |
|  |  | *mmu-miR-106b-5p* | 4,416 |  | *bta-miR-106a-5p* | 542 |
|  |  | *mmu-miR-17-5p* | 5,539 |  | *bta-miR-106b-5p* | 3,828 |
|  |  | *mmu-miR-20a-5p* | 11,650 |  | *bta-miR-17-5p* | 5,188 |
|  |  | *mmu-miR-20b-5p* | 36 |  | *bta-miR-20a-5p* | 11,357 |
|  |  | *mmu-miR-93-5p* | 3,735 |  | *bta-miR-20b-5p* | 293 |
|  |  |  |  |  | *bta-miR-93-5p* | 2,185 |
| tcacat | 24,294 | *mmu-3_28325-3p* | 286 | 46,655 | *bta-3_28453_3p* | 0.1 |
|  |  | *mmu-miR-23a-3p* | 16,105 |  | *bta-miR-23a-3p* | 29,625 |
|  |  | *mmu-miR-23b-3p* | 7,903 |  | *bta-miR-23b-3p* | 17,029 |
| ggctca | 18,310 | *mmu-miR-24-3p* | 18,310 | 20,208 | *bta-13_6408_5p* | 4 |
|  |  |  |  |  | *bta-18_13488_3p* | 2 |
|  |  |  |  |  | *bta-miR-24-3p* | 20,203 |
| attatt | 15,816 | *mmu-miR-126a-5p* | 15,816 | 24,590 | *bta-miR-126-5p* | 24,590 |
| agctta | 13,567 | *mmu-miR-21a-5p* | 13,567 | 52,748 | *bta-miR-21-5p* | 52,748 |
| aaccgt | 12,868 | *mmu-miR-451a-5p* | 12,868 | 1,130 | *bta-miR-451-5p* | 1,130 |
| accctg | 11,071 | *mmu-miR-10a-5p* | 3,496 | 7,678 | *bta-miR-10a-5p* | 520 |
|  |  | *mmu-miR-10b-5p* | 7,575 |  | *bta-miR-10b-5p* | 7,158 |
| gctaca | 8,630 | *mmu-miR-221-3p* | 7,871 | 1,649 | *bta-miR-221-3p* | 1,497 |
|  |  | *mmu-4_30034-5p* | 0.1 |  | *bta-miR-222-3p* | 152 |
|  |  | *mmu-miR-222-3p* | 759 |  |  |  |
| acagta | 7,649 | *mmu-miR-101a-3p* | 1,783 | 1,584 | *bta-miR-101-3p* | 711 |
|  |  | *mmu-miR-101b-3p* | 4,283 |  | *bta-miR-144-3p* | 14 |
|  |  | *mmu-miR-144-3p* | 1,583 |  | *bta-miR-199c-3p* | 858 |
| acccgt | 6,251 | *mmu-miR-100-5p* | 287 | 20,112 | *bta-miR-100-5p* | 1,449 |
|  |  | *mmu-miR-99a-5p* | 5,648 |  | *bta-miR-99a-5p* | 18,328 |
|  |  | *mmu-miR-99b-5p* | 315 |  | *bta-miR-99b-5p* | 335 |
| attgca | 5,463 | *mmu-miR-25-3p* | 3,295 | 13,683 | *bta-miR-25-3p* | 5,099 |
|  |  | *mmu-miR-32-5p* | 69 |  | *bta-miR-32-5p* | 129 |
|  |  | *mmu-miR-363-3p* | 6 |  | *bta-miR-92a-3p* | 8,518 |
|  |  | *mmu-miR-92a-1-3p* | 1,962 |  | *bta-miR-92b-3p* | 66 |
|  |  | *mmu-miR-92a-2-3p* | 122 |  |  |  |
|  |  | *mmu-miR-92b-3p* | 9 |  |  |  |
| cagtag | 5,423 | *mmu-miR-199a-3p* | 5,422 | 7,997 | *bta-miR-199a-3p* | 7,997 |
|  |  | *mmu-miR-222-5p* | 1 |  |  |  |
| tgtgcg | 4,383 | *mmu-miR-147-3p* | 8 |  |  |  |
|  |  | *mmu-miR-210-3p* | 4,375 |  |  |  |
| ttggca | 4,015 | *mmu-miR-96-5p* | 1,135 |  |  |  |
|  |  | *mmu-miR-182-5p* | 2,879 |  |  |  |
| gatatg | 3,960 | *mmu-miR-190a-5p* | 3,914 | 1,409 | *bta-miR-190a-5p* | 1,395 |
|  |  | *mmu-miR-190b-5p* | 46 |  | *bta-miR-190b-5p* | 14 |
| ccctga | 3,188 | *mmu-miR-125a-5p* | 2,107 | 9,715 | *bta-20_18290_5p* | 0.1 |
|  |  | *mmu-miR-125b-5p* | 944 |  | *bta-miR-125a-5p* | 4,934 |
|  |  | *mmu-miR-351-5p* | 137 |  | *bta-miR-125b-5p* | 4,781 |
| cgagga | 3,070 | *mmu-miR-151-5p* | 3,070 | 6,937 | *bta-2_18232_3p* | 0.1 |
|  |  |  |  |  | *bta-miR-151-5p* | 6,937 |
| tccagt | 3,018 | *mmu-miR-145a-5p* | 3,018 | 6,259 | *bta-12_5501_3p* | 14 |
|  |  | *mmu-miR-145b-5p* | 1 |  | *bta-14_8629_5p* | 1 |
|  |  |  |  |  | *bta-5_31373_3p* | 1 |
|  |  |  |  |  | *bta-miR-145-5p* | 6,243 |
| aaggtg | 3,006 | *mmu-miR-18a-5p* | 2,992 |  |  |  |
|  |  | *mmu-miR-18b-5p* | 14 |  |  |  |
| ggcagt | 2,939 | *mmu-miR-34a-5p* | 2,582 | 2,572 | *bta-miR-34a-5p* | 2,564 |
|  |  | *mmu-miR-34b-5p* | 188 |  | *bta-miR-34b-5p* | 4 |
|  |  | *mmu-miR-34c-5p* | 164 |  | *bta-miR-34c-5p* | 2 |
|  |  | *mmu-miR-449a-5p* | 5 |  | *bta-miR-449a-5p* | 2 |
|  |  |  |  |  | *bta-X_39471_3p* | 0.4 |
| aacgga | 2,909 | *mmu-miR-191-5p* | 2,909 | 4,845 | *bta-miR-191-5p* | 4,845 |
| ttgttc | 2,761 | *mmu-miR-375-3p* | 2,761 |  |  |  |
| tttcag | 2,455 | *mmu-miR-30a-3p* | 1,769 | 2,640 | *hsa-miR-30a-3p* | 1,744 |
|  |  | *mmu-miR-30d-3p* | 68 |  | *hsa-miR-30d-3p* | 15 |
|  |  | *mmu-miR-30e-3p* | 618 |  | *mmu-miR-30e-3p* | 882 |
| agtgca | 2,443 | *mmu-miR-130a-3p* | 1,852 |  |  |  |
|  |  | *mmu-miR-130b-3p* | 84 |  |  |  |
|  |  | *mmu-miR-301a-3p* | 493 |  |  |  |
|  |  | *mmu-miR-301b-3p* | 14 |  |  |  |
| tgaaat | 2,171 | *mmu-miR-203-3p* | 2,171 |  |  |  |
| aaagct | 1,551 | *mmu-miR-320-3p* | 1,551 | 3,460 | *bta-miR-320a-3p* | 3,460 |
| aaagaa | 1,327 | *mmu-miR-186-5p* | 1,327 | 7,316 | *bta-miR-186-5p* | 7,316 |
| aggagc | 1,281 | *mmu-miR-28a-5p* | 850 | 1,627 | *bta-miR-28-5p* | 1,575 |
|  |  | *mmu-miR-708-5p* | 431 |  | *bta-miR-708-5p* | 51 |
| gtaaca | 1,232 | *mmu-miR-194-5p* | 1,232 |  |  |  |
| aggtag | 1,152 | *mmu-miR-196b-5p* | 193 |  |  |  |
|  |  | *mmu-miR-196a-5p* | 185 |  |  |  |
|  |  | *mmu-miR-1839-5p* | 774 |  |  |  |
| ccttca | 1,149 | *mmu-miR-205-5p* | 1,149 | 4,275 | *bta-miR-205-5p* | 4,275 |
| gttctt | 1,110 | *mmu-8_42892-3p* | 0.3 |  |  |  |
|  |  | *mmu-miR-22-5p* | 1,109 |  |  |  |
|  |  | *mmu-miR-7092-3p* | 0.1 |  |  |  |
| atggca | 1,099 | *mmu-miR-183-5p* | 1,099 |  |  |  |
| gtagtg | 1,000 | *mmu-miR-142-3p* | 1,000 |  |  |  |
| gtaccg |  |  |  | 34,429 | *bta-miR-126-3p* | 34,429 |
| tagcac |  |  |  | 14,497 | *bta-miR-29a-3p* | 14,497 |
| ctacag |  |  |  | 2,982 | *bta-miR-139-5p* | 2,982 |
| ccagtg |  |  |  | 2,553 | *bta-miR-199a-5p* | 272 |
|  |  |  |  |  | *bta-miR-199b-5p* | 2,281 |
| gattcc |  |  |  | 2,241 | *hsa-miR-145-3p* | 2,241 |
| tataat |  |  |  | 2,090 | *bta-miR-374a-5p* | 659 |
|  |  |  |  |  | *bta-miR-374b-5p* | 1,430 |
| tttgtt |  |  |  | 1,991 | *bta-miR-375-3p* | 1,991 |
| gaaaag |  |  |  | 1,969 | *bta-10_2312_5p* | 1 |
|  |  |  |  |  | *bta-5_32642_3p* | 0.1 |
|  |  |  |  |  | *bta-miR-2284x-5p* | 1,968 |
| atggcg |  |  |  | 1,875 | *bta-miR-652-3p* | 1,875 |
| tagact |  |  |  | 1,761 | *bta-22_20612_5p* | 0.1 |
|  |  |  |  |  | *bta-miR-151-3p* | 1,761 |
| ctccca |  |  |  | 1,718 | *bta-miR-150-5p* | 1,699 |
|  |  |  |  |  | *hsa-miR-532-3p* | 19 |
| tccgtt |  |  |  | 1,717 | *bta-miR-1468-5p* | 1,717 |
| gagggg |  |  |  | 1,564 | *bta-14_8296_3p* | 0.2 |
|  |  |  |  |  | *bta-29_27116_5p* | 0.2 |
|  |  |  |  |  | *bta-miR-423-5p* | 1,564 |
