# Supplementary material for: Characterisation and Comparison of Lactating Mouse and Bovine Mammary Gland miRNomes
Source: PLoS One. 2014 Mar 21;9(3):e91938. doi: 10.1371/journal.pone.0091938 (PMC3962357; doi:10.1371/journal.pone.0091938)
Supplement: Table S4 — Tissues expression of the 24 common miRNA of the top 30 most expressed miRNA in bovine and mouse mammary glands. All the data considered for this comparison have been obtained by NGS technology on organs. References : Brain [67]; Endometrium [68]; Lung [69]; Muscle [70]; Liver [71]; Kidney [72]; Bladder [73]; Stomach [74]; Heart [75]. (DOCX) [file pone.0091938.s007.docx]

**Table S4. Tissues expression of the 24 common miRNA of the top 30 most expressed miRNA in bovine and mouse mammary glands.** All the data considered for this comparison have been obtained by NGS technology on organs.

| **miRNA** | **Epithelial** | **Non-epithelial** |
| --- | --- | --- |
| ***let-7a-5p*** | Endometrium | Brain, Muscle, Liver, Heart |
| ***let-7b-5p*** | Endometrium, Stomach | Brain, Liver, Heart |
| ***let-7c-5p**** | Endometrium, Lung | Brain, Muscle, Liver |
| ***let-7f-5p*** | Endometrium, Bladder | Brain, Muscle, Liver, Heart |
| ***let-7g-5p*** | Endometrium, Lung, Bladder | Brain, Muscle, Liver, Heart |
| ***let-7i-5p*** | Endometrium, Lung | Brain, Muscle |
| *miR-16-5p* | Endometrium |  |
| ***miR-21-5p*** | Endometrium, Lung, Stomach | Muscle, Liver |
| *miR-22-3p* |  |  |
| *miR-23a-3p* | Lung, Endometrium | Heart |
| ***miR-24-3p*** | Bladder, Stomach | Muscle, Heart |
| ***miR-26a-5p**** | Endometrium, Kidney, Stomach | Brain, Muscle, Liver, Heart |
| ***miR-27b-3p*** | Endometrium, Lung | Muscle, Liver |
| ***miR-29a-3p*** | Endometrium, Lung, Stomach | Brain, Liver, Heart |
| ***miR-30a-5p*** | Lung, Bladder | Muscle, Liver, Heart |
| ***miR-103-3p*** | Endometrium, Bladder | Brain, Muscle, Liver, Heart |
| *miR-126-3p* | Lung, Kidney | Heart |
| *miR-126-5p* | Kidney |  |
| *miR-141-3p* |  |  |
| ***miR-143-3p*** | Endometrium, Lung, Bladder | Brain, Muscle, Liver, Heart |
| ***miR-148a-3p*** | Endometrium, Lung, Stomach | Muscle, Liver |
| *miR-200a-3p** | Stomach |  |
| *miR-200b-3p* | Kidney, Stomach |  |
| *miR-200c-3p* | Kidney |  |

**Bold**: miRNA present in the top 30 of epithelial and non-epithelial tissues

***** miRNA expression in epithelial and non-epithelial tissues validated by RT-qPCR (figure S2).

**References** : Brain [67] ; Endometrium [68]; Lung [69]; Muscle [70] ; Liver [71]; Kidney [72] ; Bladder [73]; Stomach [74]; Heart [75].
