# Supplementary material for: Characterisation and Comparison of Lactating Mouse and Bovine Mammary Gland miRNomes
Source: PLoS One. 2014 Mar 21;9(3):e91938. doi: 10.1371/journal.pone.0091938 (PMC3962357; doi:10.1371/journal.pone.0091938)
Supplement: Table S5 — AGO2-RISC loading (p-value<0.05) of the 185 miRNA expressed in the mouse miRNome and AGO2-miRNome: (A) miRNA showing statistical equivalent abundances into both miRNomes, (B) miRNA significantly enriched in AGO2-RISC and (C) miRNA significantly less loaded in AGO2-RISC. (DOCX) [file pone.0091938.s008.docx]

**Table S5**. **AGO2-RISC loading (p-value < 0.05) of the 185 miRNA expressed in the mouse miRNome and AGO2-miRNome**: (**A**) miRNA showing statistical equivalent abundances into both miRNomes, (**B**) miRNA significantly enriched in AGO2-RISC and (**C**) miRNA significantly less loaded in AGO2-RISC.

**A.**

| **miRNA** | **Mouse** | **AGO2** | **Log2 Fold Change** | **Adjusted p-value** |
| --- | --- | --- | --- | --- |
| *mmu-11_6623* | 3,204 | 65 ^#^ | NA | NA |
| *mmu-3_28325* | 1,165 | 2,054 | -0.79 | 0.09 |
| *mmu-let-7b-5p* | 50,453 | 13,756 | 1.60 | 0.07 |
| *mmu-miR-100-5p* | 1,145 | 1,908 | -0.72 | 0.06 |
| *mmu-miR-101a-3p* | 7,466 | 10,308 | -0.44 | 0.47 |
| *mmu-miR-101b-3p* | 17,699 | 12,018 | 0.54 | 0.26 |
| *mmu-miR-106b-5p* | 17,606 | 18,049 | -0.04 | 0.94 |
| *mmu-miR-107-3p* | 28,224 | 12,995 | 1.05 | 0.09 |
| *mmu-miR-10a-5p* | 14,579 | 11,651 | 0.31 | 0.60 |
| *mmu-miR-10b-5p* | 30,769 | 38,783 | -0.33 | 0.21 |
| *mmu-miR-125a-5p* | 8,060 | 15,629 | -0.84 | 0.32 |
| *mmu-miR-125b-5p* | 3,625 | 6,454 | -0.75 | 0.34 |
| *mmu-miR-128-3p* | 788 | 489 ^#^ | 0.67 | 0.05 |
| *mmu-miR-130a-3p* | 7,596 | 6,094 | 0.29 | 0.69 |
| *mmu-miR-139-5p* | 3,346 | 3,442 | -0.04 | 0.97 |
| *mmu-miR-140-3p* | 3,538 | 3,819 | -0.10 | 0.90 |
| *mmu-miR-141-3p* | 200,291 | 488,137 | -1.19 | 0.07 |
| *mmu-miR-142-3p* | 4,232 | 3,573 | 0.22 | 0.78 |
| *mmu-miR-142-5p* | 2,762 | 6,357 | -1.13 | 0.06 |
| *mmu-miR-145a-5p* | 12,090 | 14,497 | -0.25 | 0.64 |
| *mmu-miR-146a-5p* | 6,423 | 3,128 | 0.98 | 0.09 |
| *mmu-miR-146b-3p* | 2,862 | 3,854 | -0.41 | 0.47 |
| *mmu-miR-146b-5p* | 560,809 | 351,748 | 0.65 | 0.13 |
| *mmu-miR-148a-3p* | 520,302 | 615,075 | -0.24 | 0.25 |
| *mmu-miR-148b-3p* | 9,114 | 6,758 | 0.42 | 0.31 |
| *mmu-miR-151-3p* | 1,953 | 1,991 | -0.03 | 0.98 |
| *mmu-miR-15a-5p* | 25,186 | 12,950 | 0.89 | 0.20 |
| *mmu-miR-15b-5p* | 8,451 | 6,455 | 0.36 | 0.59 |
| *mmu-miR-17-3p* | 1,216 | 732 | 0.65 | 0.43 |
| *mmu-miR-17-5p* | 21,831 | 14,586 | 0.56 | 0.24 |
| *mmu-miR-181a-5p* | 330,854 | 233,629 | 0.49 | 0.19 |
| *mmu-miR-181c-5p* | 3,681 | 5,627 | -0.60 | 0.15 |
| *mmu-miR-181d-5p* | 1,259 | 713 | 0.78 | 0.14 |
| *mmu-miR-18a-5p* | 12,341 | 5,268 | 1.07 | 0.21 |
| *mmu-miR-194-5p* | 5,068 | 6,582 | -0.36 | 0.44 |
| *mmu-miR-199a-3p* | 22,758 | 22,631 | 0.01 | 0.99 |
| *mmu-miR-199b-3p* | 22,758 | 22,631 | 0.01 | 0.99 |
| *mmu-miR-19b-3p* | 836 | 1,163 | -0.41 | 0.66 |
| *mmu-miR-200a-5p* | 876 | 1,494 | -0.73 | 0.18 |
| *mmu-miR-200b-5p* | 2,150 | 1,421 | 0.55 | 0.43 |
| *mmu-miR-200c-3p* | 67,118 | 19,845 | 1.53 | 0.07 |
| *mmu-miR-205-5p* | 4,575 | 5,264 | -0.20 | 0.60 |
| *mmu-miR-214-3p* | 1,155 | 442 ^#^ | 1.12 | 0.27 |
| *mmu-miR-218-5p* | 738 | 350 ^#^ | 0.86 | 0.42 |
| *mmu-miR-21a-5p* | 57,089 | 58,308 | -0.03 | 0.98 |
| *mmu-miR-223-3p* | 1,508 | 2,579 | -0.75 | 0.08 |
| *mmu-miR-23a-3p* | 64,184 | 79,217 | -0.30 | 0.43 |
| *mmu-miR-23b-3p* | 31,178 | 41,772 | -0.41 | 0.43 |
| *mmu-miR-25-3p* | 13,281 | 11,116 | 0.25 | 0.64 |
| *mmu-miR-27a-3p* | 43,782 | 90,185 | -0.99 | 0.07 |
| *mmu-miR-27b-3p* | 63,609 | 64,796 | -0.03 | 0.94 |
| *mmu-miR-28a-3p* | 1,301 | 709 | 0.80 | 0.27 |
| *mmu-miR-29a-5p* | 750 | 679 | 0.14 | 0.70 |
| *mmu-miR-29b-3p* | 52,210 | 85,543 | -0.66 | 0.35 |
| *mmu-miR-301a-3p* | 2,061 | 1,487 | 0.41 | 0.64 |
| *mmu-miR-30a-3p* | 7,172 | 5,174 | 0.45 | 0.38 |
| *mmu-miR-30a-5p* | 113,793 | 231,487 | -0.94 | 0.17 |
| *mmu-miR-30e-3p* | 2,479 | 3,238 | -0.36 | 0.57 |
| *mmu-miR-30e-5p* | 34,157 | 40,471 | -0.24 | 0.64 |
| *mmu-miR-3107-5p* | 1,741 | 4,455 | NA | NA |
| *mmu-miR-320-3p* | 5,814 | 1,612 | NA | NA |
| *mmu-miR-322-5p* | 11,240 | 10,539 | 0.09 | 0.90 |
| *mmu-miR-335-5p* | 942 | 266 ^#^ | 1.48 | 0.14 |
| *mmu-miR-338-3p* | 3,564 | 3,032 | 0.21 | 0.80 |
| *mmu-miR-340-3p* | 828 | 1,293 | -0.58 | 0.44 |
| *mmu-miR-340-5p* | 3,965 | 5,275 | -0.40 | 0.44 |
| *mmu-miR-342-3p* | 1,089 | 2,145 | -0.92 | 0.12 |
| *mmu-miR-34b-5p* | 756 | 360 ^#^ | 1.02 | 0.06 |
| *mmu-miR-362-5p* | 2,829 | 2,782 | 0.02 | 0.98 |
| *mmu-miR-365-3p* | 599 | 1,136 | -0.80 | 0.38 |
| *mmu-miR-374b-5p* | 1,334 | 2,101 | -0.57 | 0.51 |
| *mmu-miR-378c-5p* | 28,718 | 19,633 | 0.54 | 0.17 |
| *mmu-miR-411-5p* | 723 | 1,914 | -1.19 | 0.19 |
| *mmu-miR-423-5p* | 657 | 297 ^#^ | 0.93 | 0.37 |
| *mmu-miR-425-5p* | 1,061 | 1,389 | -0.35 | 0.66 |
| *mmu-miR-429-3p* | 34,444 | 21,175 | 0.67 | 0.18 |
| *mmu-miR-450a-5p* | 3,825 | 3,906 | -0.03 | 0.98 |
| *mmu-miR-451a-5p* | 50,329 | 113,642 | -1.06 | 0.16 |
| *mmu-miR-486-5p* | 1,743 | 4,450 | NA | NA |
| *mmu-miR-501-3p* | 517 ^#^ | 759 | -0.50 | 0.50 |
| *mmu-miR-503-5p* | 622 | 343 ^#^ | 0.82 | 0.12 |
| *mmu-miR-532-5p* | 2,561 | 2,264 | 0.18 | 0.57 |
| *mmu-miR-652-3p* | 2,796 | 1,021 | 1.26 | 0.15 |
| *mmu-miR-676-3p* | 859 | 344 ^#^ | 1.16 | 0.17 |
| *mmu-miR-92a-3p* | 7,549 | 7,270 | 0.05 | 0.98 |
| *mmu-miR-93-5p* | 14,846 | 9,853 | 0.57 | 0.28 |
| *mmu-miR-96-5p* | 4,732 | 3,458 | 0.43 | 0.49 |

^#^: Expression under 100 RPM.

NA: Counts between the two samples too far from the negative binomial distribution to allow the statistical test.

**B.**

| **miRNA** | **Mouse** | **AGO2** | **Log2 Fold Change** | **Adjusted p-value** |
| --- | --- | --- | --- | --- |
| *mmu-15_15267* | 17 ^#^ | 853 | -5.25 | 5.14E-21 |
| *mmu-2_24638* | 448 ^#^ | 727 | -0.69 | 2.00E-02 |
| *mmu-8_43005* | 2 ^#^ | 1,142 | -7.72 | 6.89E-26 |
| *mmu-let-7a-1-3p* | 349 ^#^ | 2,336 | -2.62 | 1.29E-07 |
| *mmu-let-7c-2-3p* | 349 ^#^ | 2,336 | -2.62 | 1.29E-07 |
| *mmu-let-7d-3p* | 247 | 44,619 | -7.24 | 3.05E-72 |
| *mmu-miR-126a-5p* | 65,906 | 142,400 | -1.06 | 4.54E-02 |
| *mmu-miR-136-3p* | 331 ^#^ | 1,940 | -2.15 | 1.81E-02 |
| *mmu-miR-144-3p* | 6,400 | 32,439 | -2.31 | 3.62E-19 |
| *mmu-miR-150-5p* | 1,530 | 4,514 | -1.55 | 2.47E-11 |
| *mmu-miR-181a-1-3p* | 3,276 | 8,612 | -1.35 | 2.08E-03 |
| *mmu-miR-186-5p* | 5,461 | 13,231 | -1.24 | 1.26E-03 |
| *mmu-miR-199a-5p* | 1,707 | 4,839 | -1.42 | 1.11E-02 |
| *mmu-miR-199b-5p* | 783 | 2,310 | -1.43 | 3.74E-02 |
| *mmu-miR-200a-3p* | 587,081 | 1,671,001 | -1.45 | 1.84E-03 |
| *mmu-miR-29a-3p* | 191,066 | 418,945 | -1.08 | 3.86E-02 |
| *mmu-miR-29c-3p* | 4,269 | 12,637 | -1.48 | 1.02E-02 |
| *mmu-miR-30b-5p* | 2,130 | 10,076 | -2.08 | 1.13E-03 |
| *mmu-miR-30c-5p* | 3,148 | 10,196 | -1.55 | 2.94E-02 |
| *mmu-miR-30d-5p* | 28,518 | 56,539 | -0.98 | 1.37E-06 |
| *mmu-miR-32-5p* | 291 ^#^ | 1,359 | -1.97 | 1.27E-02 |
| *mmu-miR-33-5p* | 380 ^#^ | 2,625 | -2.53 | 3.60E-04 |
| *mmu-miR-339-5p* | 185 ^#^ | 992 | -2.34 | 5.52E-08 |
| *mmu-miR-345-5p* | 284 ^#^ | 963 | -1.64 | 9.07E-03 |
| *mmu-miR-350-3p* | 49 ^#^ | 1,760 | -3.62 | 1.07E-03 |
| *mmu-miR-351-5p* | 540 ^#^ | 1,273 | -1.18 | 1.89E-02 |
| *mmu-miR-362-3p* | 94 ^#^ | 1,443 | -3.61 | 3.78E-08 |
| *mmu-miR-376a-3p* | 55 ^#^ | 1,423 | -4.08 | 2.99E-07 |
| *mmu-miR-378a-5p* | 2,389 | 6,939 | -1.42 | 3.34E-02 |
| *mmu-miR-455-3p* | 220 ^#^ | 986 | -2.02 | 1.13E-03 |
| *mmu-miR-484-5p* | 946 | 6,884 | -2.58 | 3.95E-04 |
| *mmu-miR-500-3p* | 1,900 | 4,962 | -1.30 | 2.75E-02 |
| *mmu-miR-532-3p* | 86 ^#^ | 1,438 | -3.41 | 1.26E-04 |
| *mmu-miR-574-3p* | 30 ^#^ | 671 | -3.84 | 3.11E-06 |
| *mmu-miR-582-5p* | 225 ^#^ | 1,281 | -2.17 | 1.18E-02 |
| *mmu-miR-7a-1-3p* | 157 ^#^ | 1,373 | -2.87 | 1.59E-05 |
| *mmu-miR-99a-5p* | 22,940 | 45,648 | -0.98 | 2.10E-04 |
| *mmu-miR-99b-5p* | 1,217 | 5,784 | -2.05 | 3.68E-03 |

^#^: Expression under 100 RPM.

**C.**

| **miRNA** | **Mouse** | **AGO2** | **Log2 Fold Change** | **Adjusted p-value** |
| --- | --- | --- | --- | --- |
| *mmu-let-7a-5p* | 103,890 | 19,304 | 2.26 | 2.10E-04 |
| *mmu-let-7c-5p* | 112,994 | 19,029 | 2.31 | 1.89E-03 |
| *mmu-let-7d-5p* | 40,755 | 4,115 | 3.04 | 3.51E-06 |
| *mmu-let-7e-5p* | 5,066 | 1,197 | 1.81 | 3.26E-02 |
| *mmu-let-7f-5p* | 114,475 | 25,730 | 2.06 | 2.69E-05 |
| *mmu-let-7g-5p* | 153,631 | 33,223 | 2.11 | 1.97E-05 |
| *mmu-let-7i-5p* | 120,241 | 22,813 | 2.31 | 9.03E-08 |
| *mmu-let-7j* | 906 | 77 ^#^ | 3.34 | 2.42E-09 |
| *mmu-miR-103-3p* | 123,506 | 26,391 | 2.07 | 1.05E-03 |
| *mmu-miR-126a-3p* | 110,650 | 43,061 | 1.33 | 9.75E-05 |
| *mmu-miR-140-5p* | 2,000 | 645 | 1.60 | 5.61E-07 |
| *mmu-miR-141-5p* | 1,865 | 579 ^#^ | 1.60 | 3.85E-03 |
| *mmu-miR-143-3p* | 341,011 | 188,338 | 0.84 | 2.73E-02 |
| *mmu-miR-145a-3p* | 2,829 | 1,707 | 0.71 | 4.23E-02 |
| *mmu-miR-151-5p* | 12,070 | 4,084 | 1.48 | 7.35E-03 |
| *mmu-miR-152-3p* | 27,430 | 13,349 | 1.03 | 4.25E-05 |
| *mmu-miR-16-5p* | 75,609 | 34,979 | 1.09 | 3.25E-03 |
| *mmu-miR-181b-5p* | 61,253 | 24,340 | 1.30 | 1.11E-03 |
| *mmu-miR-182-5p* | 11,462 | 4,079 | 1.46 | 1.84E-05 |
| *mmu-miR-183-5p* | 4,409 | 1,493 | 1.47 | 1.18E-02 |
| *mmu-miR-1839-5p* | 3,057 | 335 ^#^ | 3.02 | 1.07E-08 |
| *mmu-miR-1843a-5p* | 810 | 433 ^#^ | 0.88 | 2.94E-02 |
| *mmu-miR-1843b-5p* | 1,130 | 501 ^#^ | 1.12 | 3.91E-02 |
| *mmu-miR-185-5p* | 2,295 | 236 ^#^ | 3.19 | 1.77E-17 |
| *mmu-miR-187-3p* | 1,088 | 364 ^#^ | 1.46 | 2.95E-02 |
| *mmu-miR-190a-5p* | 16,519 | 2,770 | 2.34 | 8.97E-04 |
| *mmu-miR-191-5p* | 11,353 | 4,598 | 1.23 | 3.15E-02 |
| *mmu-miR-193a-5p* | 620 | 92 ^#^ | 2.30 | 1.25E-02 |
| *mmu-miR-195a-5p* | 7,953 | 2,901 | 1.41 | 6.06E-04 |
| *mmu-miR-196a-5p* | 750 | 39 ^#^ | 3.60 | 3.32E-05 |
| *mmu-miR-196b-5p* | 802 | 247 ^#^ | 1.58 | 1.36E-02 |
| *mmu-miR-1a-3p* | 1,105 | 151 ^#^ | 2.51 | 2.08E-03 |
| *mmu-miR-200b-3p* | 154,732 | 55,108 | 1.41 | 1.31E-02 |
| *mmu-miR-203-3p* | 8,631 | 4,610 | 0.89 | 1.29E-02 |
| *mmu-miR-20a-5p* | 46,953 | 32,010 | 0.55 | 1.17E-02 |
| *mmu-miR-210-3p* | 17,433 | 4,355 | 1.97 | 3.74E-11 |
| *mmu-miR-221-3p* | 31,854 | 15,461 | 1.03 | 3.31E-04 |
| *mmu-miR-222-3p* | 3,013 | 1,626 | 0.87 | 2.28E-02 |
| *mmu-miR-22-3p* | 134,847 | 61,593 | 1.11 | 4.20E-04 |
| *mmu-miR-22-5p* | 4,510 | 800 | 2.46 | 3.62E-19 |
| *mmu-miR-24-3p* | 74,230 | 18,653 | 1.98 | 1.72E-24 |
| *mmu-miR-26a-5p* | 155,546 | 96,940 | 0.67 | 3.81E-02 |
| *mmu-miR-26b-5p* | 46,515 | 19,882 | 1.19 | 4.15E-03 |
| *mmu-miR-28a-5p* | 3,337 | 838 | 1.87 | 1.80E-03 |
| *mmu-miR-3068-5p* | 3,915 | 976 | 1.89 | 6.43E-04 |
| *mmu-miR-3105-5p* | 814 | 291 ^#^ | 1.38 | 2.86E-02 |
| *mmu-miR-31-5p* | 2,876 | 383 ^#^ | 2.80 | 2.93E-10 |
| *mmu-miR-324-5p* | 978 | 347 ^#^ | 1.40 | 2.21E-02 |
| *mmu-miR-345-3p* | 876 | 221 ^#^ | 1.85 | 3.85E-03 |
| *mmu-miR-34a-5p* | 10,577 | 994 | 3.20 | 1.20E-08 |
| *mmu-miR-34c-5p* | 651 | 32 ^#^ | 3.52 | 1.95E-04 |
| *mmu-miR-361-5p* | 1,335 | 477 ^#^ | 1.46 | 1.35E-06 |
| *mmu-miR-375-3p* | 10,747 | 3,164 | 1.64 | 9.88E-03 |
| *mmu-miR-378a-3p* | 74,696 | 33,006 | 1.16 | 1.56E-04 |
| *mmu-miR-378d-5p* | 2,365 | 727 | 1.64 | 1.82E-04 |
| *mmu-miR-497-5p* | 22,956 | 13,415 | 0.76 | 1.48E-02 |
| *mmu-miR-674-5p* | 1,250 | 60 ^#^ | 2.78 | 1.99E-02 |
| *mmu-miR-708-5p* | 1,784 | 827 | 1.07 | 2.94E-02 |
| *mmu-miR-7a-5p* | 3,758 | 518 ^#^ | 2.77 | 1.86E-12 |
| *mmu-miR-98-5p* | 3,443 | 445 ^#^ | 2.74 | 1.21E-05 |

^#^: Expression under 100 RPM.
