# Supplementary material for: Characterisation and Comparison of Lactating Mouse and Bovine Mammary Gland miRNomes
Source: PLoS One. 2014 Mar 21;9(3):e91938. doi: 10.1371/journal.pone.0091938 (PMC3962357; doi:10.1371/journal.pone.0091938)
Supplement: Table S6 — Pathways targeted by miRNA highly expressed in mouse and bovine mammary gland. (DOCX) [file pone.0091938.s009.docx]

**Table S6. Pathways targeted by miRNA highly expressed** **in mouse and bovine mammary gland.**

|  | **24 common miRNA**  **of the top 30** | | | **6 miRNA highly expressed in lactating mammary gland** | | |
| --- | --- | --- | --- | --- | --- | --- |
| **KEGG pathway** | **p-value** | **Number of genes** | **Number of miRNA** | **p-value** | **Number of genes** | **Number of miRNA** |
| PI3K-Akt signaling pathway | 8.10E-35 | 131 | 23 | 1.48E-06 | 46 | 6 |
| Focal adhesion | 8.63E-28 | 82 | 23 | 1.31E-06 | 31 | 6 |
| Axon guidance | 3.15E-26 | 64 | 23 | 2.47E-07 | 26 | 6 |
| ErbB signaling pathway | 8.10E-23 | 41 | 23 | 1.22E-12 | 21 | 6 |
| Protein digestion and absorption | 4.78E-20 | 39 | 21 | 4.06E-02 | 11 | 6 |
| MAPK signaling pathway | 8.74E-20 | 94 | 23 | 1.31E-06 | 38 | 6 |
| mTOR signaling pathway | 1.78E-17 | 31 | 21 | 5.89E-07 | 15 | 5 |
| Regulation of actin cytoskeleton | 3.46E-17 | 80 | 23 |  |  |  |
| Transcriptional misregulation in cancer | 1.99E-14 | 66 | 24 |  |  |  |
| Ubiquitin mediated proteolysis | 4.12E-14 | 55 | 22 | 4.55E-04 | 21 | 5 |
| Insulin signaling pathway | 6.79E-14 | 52 | 23 | 5.25E-04 | 20 | 6 |
| Prostate cancer | 6.79E-14 | 37 | 23 | 3.45E-04 | 15 | 6 |
| Acute myeloid leukemia | 8.71E-14 | 26 | 23 | 1.44E-04 | 12 | 6 |
| Pathways in cancer | 2.96E-12 | 110 | 23 | 3.02E-04 | 43 | 6 |
| Long-term potentiation | 5.34E-12 | 29 | 23 |  |  |  |
| T cell receptor signaling pathway | 1.85E-11 | 41 | 23 | 2.63E-06 | 19 | 6 |
| Protein processing in endoplasmic reticulum | 2.66E-10 | 60 | 22 |  |  |  |
| Melanoma | 6.66E-10 | 30 | 22 |  |  |  |
| Circadian rhythm | 8.06E-10 | 16 | 21 | 9.92E-06 | 9 | 6 |
| Renal cell carcinoma | 8.06E-10 | 32 | 23 | 2.83E-07 | 17 | 6 |
| Neurotrophin signaling pathway | 9.34E-10 | 45 | 23 | 3.19E-06 | 22 | 6 |
| Chronic myeloid leukemia | 3.38E-09 | 30 | 23 | 1.51E-03 | 13 | 6 |
| Hedgehog signaling pathway | 6.86E-09 | 21 | 20 | 4.82E-09 | 14 | 6 |
| Endometrial cancer | 2.62E-08 | 22 | 23 | 1.45E-03 | 10 | 6 |
| Glycosaminoglycan biosynthesis - heparan sulfate / heparin | 2.62E-08 | 14 | 19 | 1.73E-02 | 5 | 5 |
| Gap junction | 3.68E-08 | 31 | 21 | 2.47E-07 | 16 | 6 |
| Wnt signaling pathway | 4.35E-08 | 54 | 23 | 3.81E-04 | 25 | 6 |
| Small cell lung cancer | 6.67E-08 | 31 | 22 |  |  |  |
| Jak-STAT signaling pathway | 8.09E-08 | 50 | 23 |  |  |  |
| Hypertrophic cardiomyopathy (HCM) | 4.46E-07 | 30 | 20 |  |  |  |
| Osteoclast differentiation | 5.92E-07 | 41 | 23 | 1.59E-02 | 16 | 6 |
| HTLV-I infection | 7.85E-07 | 83 | 23 | 7.38E-07 | 40 | 6 |
| p53 signaling pathway | 1.14E-06 | 26 | 22 | 1.76E-04 | 13 | 6 |
| Basal cell carcinoma | 1.61E-06 | 21 | 21 | 2.48E-03 | 9 | 6 |
| VEGF signaling pathway | 1.64E-06 | 24 | 23 | 1.94E-03 | 11 | 6 |
| Fc gamma R-mediated phagocytosis | 1.70E-06 | 31 | 23 | 2.63E-06 | 17 | 6 |
| Apoptosis | 4.42E-06 | 31 | 23 |  |  |  |
| Glioma | 8.01E-06 | 27 | 23 | 1.94E-03 | 12 | 6 |
| Amoebiasis | 9.18E-06 | 36 | 22 |  |  |  |
| Calcium signaling pathway | 3.09E-05 | 54 | 22 |  |  |  |
| Glutamatergic synapse | 3.09E-05 | 38 | 22 | 4.63E-02 | 14 | 6 |
| Dopaminergic synapse | 4.26E-05 | 42 | 23 | 3.41E-02 | 16 | 6 |
| Butanoate metabolism | 5.06E-05 | 12 | 15 |  |  |  |
| N-Glycan biosynthesis | 1.07E-04 | 20 | 18 | 2.77E-02 | 8 | 4 |
| Pancreatic cancer | 1.12E-04 | 25 | 22 | 1.57E-02 | 11 | 5 |
| Dilated cardiomyopathy | 1.23E-04 | 29 | 20 |  |  |  |
| Lysine degradation | 1.35E-04 | 18 | 20 | 2.50E-02 | 7 | 6 |
| Neuroactive ligand-receptor interaction | 2.01E-04 | 76 | 24 |  |  |  |
| Melanogenesis | 2.10E-04 | 32 | 22 |  |  |  |
| GnRH signaling pathway | 2.55E-04 | 28 | 21 | 5.19E-03 | 13 | 6 |
| Non-small cell lung cancer | 2.55E-04 | 20 | 23 | 7.06E-03 | 10 | 6 |
| Dorso-ventral axis formation | 5.80E-04 | 9 | 18 | 3.02E-04 | 6 | 6 |
| Adherens junction | 6.09E-04 | 27 | 21 | 8.92E-03 | 12 | 6 |
| Glycosaminoglycan biosynthesis - keratan sulfate | 6.09E-04 | 7 | 11 | 8.92E-03 | 6 | 6 |
| Chagas disease (American trypanosomiasis) | 8.36E-04 | 31 | 23 |  |  |  |
| HIF-1 signaling pathway | 8.36E-04 | 35 | 22 | 2.23E-02 | 15 | 5 |
| mRNA surveillance pathway | 8.36E-04 | 30 | 15 |  |  |  |
| Endocytosis | 8.48E-04 | 62 | 23 | 1.59E-02 | 25 | 6 |
| Colorectal cancer | 1.46E-03 | 21 | 23 |  |  |  |
| ECM-receptor interaction | 1.46E-03 | 32 | 21 |  |  |  |
| Fatty acid biosynthesis | 1.46E-03 | 1 | 1 |  |  |  |
| Fc epsilon RI signaling pathway | 1.46E-03 | 23 | 23 | 1.94E-03 | 12 | 6 |
| SNARE interactions in vesicular transport | 1.46E-03 | 13 | 18 |  |  |  |
| GABAergic synapse | 1.70E-03 | 31 | 21 | 3.76E-03 | 14 | 5 |
| Hepatitis B | 3.97E-03 | 46 | 23 | 1.92E-03 | 22 | 6 |
| Alanine, aspartate and glutamate metabolism | 4.17E-03 | 12 | 10 |  |  |  |
| B cell receptor signaling pathway | 6.59E-03 | 23 | 22 | 5.31E-03 | 12 | 6 |
| Regulation of autophagy | 6.74E-03 | 12 | 16 |  |  |  |
| Progesterone-mediated oocyte maturation | 1.19E-02 | 25 | 22 |  |  |  |
| Adipocytokine signaling pathway | 1.28E-02 | 20 | 21 |  |  |  |
| Inositol phosphate metabolism | 1.28E-02 | 18 | 13 |  |  |  |
| Amphetamine addiction | 1.35E-02 | 27 | 22 |  |  |  |
| Amyotrophic lateral sclerosis (ALS) | 1.35E-02 | 17 | 18 |  |  |  |
| Cocaine addiction | 1.54E-02 | 18 | 13 |  |  |  |
| Type II diabetes mellitus | 1.92E-02 | 16 | 20 |  |  |  |
| Biotin metabolism | 2.47E-02 | 1 | 1 |  |  |  |
| Nicotine addiction | 2.49E-02 | 15 | 15 |  |  |  |
| Phosphatidylinositol signaling system | 2.51E-02 | 26 | 22 |  |  |  |
| Cytokine-cytokine receptor interaction | 2.65E-02 | 67 | 23 |  |  |  |
| Thyroid cancer | 2.65E-02 | 9 | 15 |  |  |  |
| TGF-beta signaling pathway | 2.67E-02 | 26 | 21 | 5.67E-03 | 13 | 6 |
| RNA degradation | 3.73E-02 | 22 | 20 |  |  |  |
| Long-term depression | 4.49E-02 | 20 | 22 | 4.83E-02 | 9 | 5 |
| Pentose and glucuronate interconversions |  |  |  | 3.59E-03 | 7 | 2 |
| Ascorbate and aldarate metabolism |  |  |  | 3.24E-03 | 6 | 1 |
| Viral carcinogenesis |  |  |  | 2.89E-03 | 31 | **6** |
| Oocyte meiosis |  |  |  | 2.46E-03 | 17 | **6** |
| Natural killer cell mediated cytotoxicity |  |  |  | 4.27E-02 | 16 | **6** |
| RIG-I-like receptor signaling pathway |  |  |  | 4.05E-02 | 9 | **6** |
| Porphyrin and chlorophyll metabolism |  |  |  | 2.24E-02 | 7 | 3 |
| Maturity onset diabetes of the young |  |  |  | 2.18E-02 | 5 | 4 |
| Tight junction |  |  |  | 1.16E-02 | 19 | **6** |
