# Supplementary material for: Characterisation and Comparison of Lactating Mouse and Bovine Mammary Gland miRNomes
Source: PLoS One. 2014 Mar 21;9(3):e91938. doi: 10.1371/journal.pone.0091938 (PMC3962357; doi:10.1371/journal.pone.0091938)
Supplement: Table S7 — Major pathways targeted by the predicted novel miRNA highly expressed in mouse and bovine mammary gland. (DOCX) [file pone.0091938.s010.docx]

**Table S7.** **Major pathways targeted by the predicted novel miRNA highly expressed in mouse and bovine mammary gland**

| **Mouse KEGG Pathway** | **p-value** | **Bovine KEGG Pathway** | **p-value** |
| --- | --- | --- | --- |
| Apoptosis | 8.6E-5 | Axon guidance | 2.2E-1 |
| BTB/POZ | 3.9E-4 | Cell fate specification | 6.2E-4 |
| Calmodulin-binding | 1.0E-3 | Cell-cell signaling | 3.1E-3 |
| Cell migration | 1.2E-1 | Cellular component morphogenesis | 1.1E-1 |
| Chromatin modification | 3.7E-1 | Cellular protein catabolic process | 1.7E-2 |
| Cytoskeletal protein binding | 8.0E-2 | Chromatin modification | 9.4E-5 |
| DNA-binding | 6.5E-10 | Embryonic appendage morphogenesis | 4.3E-3 |
| Embryonic morphogenesis | 6.6E-6 | **Epithelium development** | 9.2E-6 |
| Endocytosis | 6.1E-2 | Forebrain neuron differentiation | 8.6E-4 |
| ErbB signaling pathway | 1.2E-3 | Gtpase regulator activity | 6.5E-2 |
| Focal adhesion | 9.6E-4 | **Mammary gland epithelial cell proliferation** | 3.3E-2 |
| Gene silencing | 8.0E-3 | **Mammary gland morphogenesis** | 9.1E-2 |
| Hemopoietic or lymphoid organ development | 2.1E-5 | Metal ion binding | 2.7E-3 |
| Homeostasis of number of cells | 2.4E-2 | Methylation | 4.1E-3 |
| Insulin signaling pathway | 4.1E-1 | Negative regulation of cellular protein metabolic process | 3.0E-2 |
| Intracellular transport | 1.9E-3 | Negative regulation of transcription | 4.1E-13 |
| Kinase regulator activity | 9.3E-3 | Neuron differentiation | 2.8E-4 |
| **Lactation** | 1.5E-2 | Nucleotide binding | 1.6E-4 |
| Lipid binding | 5.7E-1 | Phosphate metabolic process | 3.7E-2 |
| **Mammary gland development** | 5.0E-2 | Positive regulation of cell differentiation | 1.8E-3 |
| MAPKKK cascade | 2.0E-4 | Positive regulation of gene expression | 2.4E-11 |
| Mesenchymal cell differentiation | 6.4E-2 | Positive regulation of signal transduction | 1.1E-2 |
| Metal ion binding | 1.8E-2 | Posttranscriptional regulation of gene expression | 2.3E-2 |
| mRNA transport | 1.1E-1 | Protein dimerization activity | 2.7E-3 |
| mTOR signaling pathway | 4.9E-1 | Protein domain specific binding | 5.3E-7 |
| Myosin | 4.1E-1 | Protein kinase binding | 3.2E-2 |
| Negative regulation of gene expression | 1.4E-6 | Protein serine/threonine kinase activity | 4.5E-3 |
| Nucleotide binding | 7.9E-3 | Regionalization | 2.3E-2 |
| Pattern specification process | 2.0E-4 | Regulation of apoptosis | 4.5E-3 |
| Phosphate metabolic process | 1.6E-8 | Regulation of cell development | 7.1E-4 |
| Phosphorylation | 7.4E-9 | Regulation of cytokine biosynthetic process | 5.7E-2 |
| Positive regulation of gene expression | 1.7E-9 | **Regulation of epithelial cell differentiation** | 5.1E-3 |
| Post-transcriptional regulation of gene expression | 8.8E-2 | **Regulation of epithelial cell proliferation** | 1.5E-1 |
| Protein catabolic process | 1.1E-3 | Regulation of neurological system process | 1.2E-2 |
| Protein dimerization activity | 3.0E-4 | Regulation of RNA metabolic process | 2.4E-15 |
| Protein domain specific binding | 1.6E-5 | Regulation of transcription | 5.5E-23 |
| Regulation of cell development | 3.7E-1 | Reproductive developmental process | 4.2E-4 |
| Regulation of RNA metabolic process | 9.7E-8 | Rhythmic process | 1.5E-2 |
| Sensory organ development | 1.4E-2 | RNA binding | 2.2E-3 |
| Sequence-specific DNA binding | 6.2E-5 | Small GTPase mediated signal transduction | 2.0E-3 |
| Transcription regulation | 2.6E-10 | Stem cell development | 3.8E-2 |
| Transforming growth factor beta receptor activity | 7.4E-2 | Stem cell maintenance | 3.3E-2 |
| Tube development | 1.4E-3 | Steroid hormone receptor activity | 2.4E-2 |
| Ubiquitin mediated proteolysis | 2.8E-3 | Transcription factor binding | 7.8E-4 |
| Vasculature development | 2.0E-3 | Ubiquitin mediated proteolysis | 3.1E-4 |
| Wnt signaling pathway | 5.6E-3 | Wnt signaling pathway | 7.4E-3 |
